# Anion-driven encapsulation of cationic guests inside pyridine[4]arene dimers

**DOI:** 10.3762/bjoc.15.241

**Published:** 2019-10-21

**Authors:** Anniina Kiesilä, Jani O Moilanen, Anneli Kruve, Christoph A Schalley, Perdita Barran, Elina Kalenius

**Affiliations:** 1 Department of Chemistry, Nanoscience Center, University of Jyväskylä, P.O. Box 35, University of Jyväskylä, Finland; 2 Institut für Chemie und Biochemie, Freie Universität Berlin, Takustrasse 3, 14195 Berlin, Germany; 3 Michael Barber Centre for Collaborative Mass Spectrometry, Manchester Institute of Biotechnology, School of Chemistry, The University of Manchester, Princess Street, Manchester, UK

**Keywords:** cation binding, DFT calculations, ion mobility mass spectrometry, macrocycles, pyridinearenes, resorcinarenes

## Abstract

Pyridine[4]arenes have previously been considered as anion binding hosts due to the electron-poor nature of the pyridine ring. Herein, we demonstrate the encapsulation of Me_4_N^+^ cations inside a dimeric hydrogen-bonded pyridine[4]arene capsule, which contradicts with earlier assumptions. The complexation of a cationic guest inside the pyridine[4]arene dimer has been detected and studied by multiple gas-phase techniques, ESI-QTOF-MS, IRMPD, and DT-IMMS experiments, as well as DFT calculations. The comparison of classical resorcinarenes with pyridinearenes by MS and NMR experiments reveals clear differences in their host–guest chemistry and implies that cation encapsulation in pyridine[4]arene is an anion-driven process.

## Introduction

**Scheme 1:**
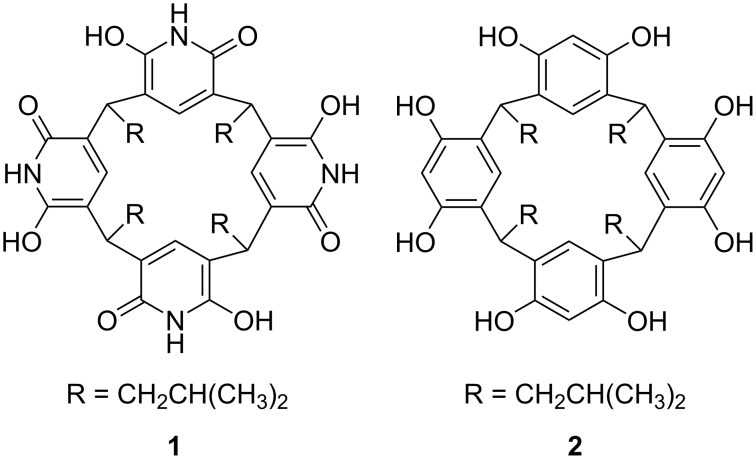
Structures of tetraisobutylpyridine[4]arene **1** and tetraisobutylresorcin[4]arene **2**.

Resorcinarenes and their derivatives are known for the molecular recognition properties of their self-assembled dimeric and hexameric capsules, which can encapsulate cationic and neutral guests [1–3]. Pyridine[4]arenes [4] are analogous macrocycles to resorcin[4]arenes. Whereas resorcinarenes are cyclic tetramers of resorcinol, pyridinearenes are formed from 2,6-dihydroxypyridine (see Scheme 1). Although the synthesis of pyridine[4]arenes dates back to 2001 [4], their host–guest chemistry is still under-explored. Both macrocycles are concave and are known to form capsular assemblies via intermolecular hydrogen bonding [5–6]. Pyridine is significantly less electron-rich than benzene. Consequently, pyridinearene capsules were originally assumed to encapsulate anionic guests inside their cavity due to the π-acidic character of the aromatic walls [7–8]. Mattey et al. detected 1:1 complex formation with PF_6_^−^ and BF_4_^−^ by mass spectrometry, however, without ion mobility mass spectrometry, the location of the anion could not be verified and the anions were assumed to interact with the pyridinearene cavity. Inclusion complexes of anions within pyridinearene dimers were also theoretically studied by DFT calculations, but using a truncated pyridinearene dimer model [8]. However, previous studies have also shown that the π-acidic character of pyridine rings, such as 2-oxo-6-oxypyridine, is rather weak [9–10]. Thus, pyridine[4]arenes may be expected to show a dual binding behavior towards anions and cations [8]. In addition to anion complexes also 2:1 complex formation with neutral carboxylic acids and amides have been previously detected by ESI-MS [7]. Very recently, with the help of ion mobility mass spectrometry (IM-MS), we showed that pyridine[4]arenes favor encapsulation of neutral molecules over anionic species and anions are in fact complexed in an *exo*-position (exclusion complexation) between the lower-rim alkyl chains [11]. A PF_6_^−^ anion was bound to the lower rim of the pyridine[4]arene dimer via CH–anion and CH–F interactions, while the neutral guest was hosted inside the dimer. Calculated electrostatic potential (ESP) surfaces revealed that the cavity does not possess any significant partial positive potential on the surface of the cavity, except on the N–H hydrogen atoms. They are, however, on tangential positions along the capsules surface, and therefore do not significantly contribute to anion binding [8]. More recently, we were also able to demonstrate that cationic transition metal complexes can template the formation of pyridinearene hexameric capsules in the gas phase [6].

Here, we report our novel findings on the ability of dimeric tetraisobutylpyridine[4]arene (compound **1** in Scheme 1) to encapsulate cationic guests. Despite of the obvious structural similarities between the dimeric resorcin[4]arene and pyridine[4]arene capsules, we highlight here unique host–guest properties of pyridinearene capsules. In marked contrast to the corresponding resorcin[4]arene capsules, cation binding is clearly feasible, when anions bind in an *exo*-site and support cation encapsulation by "through-wall" electrostatic interactions.

## Results and Discussion

**Figure 1:**
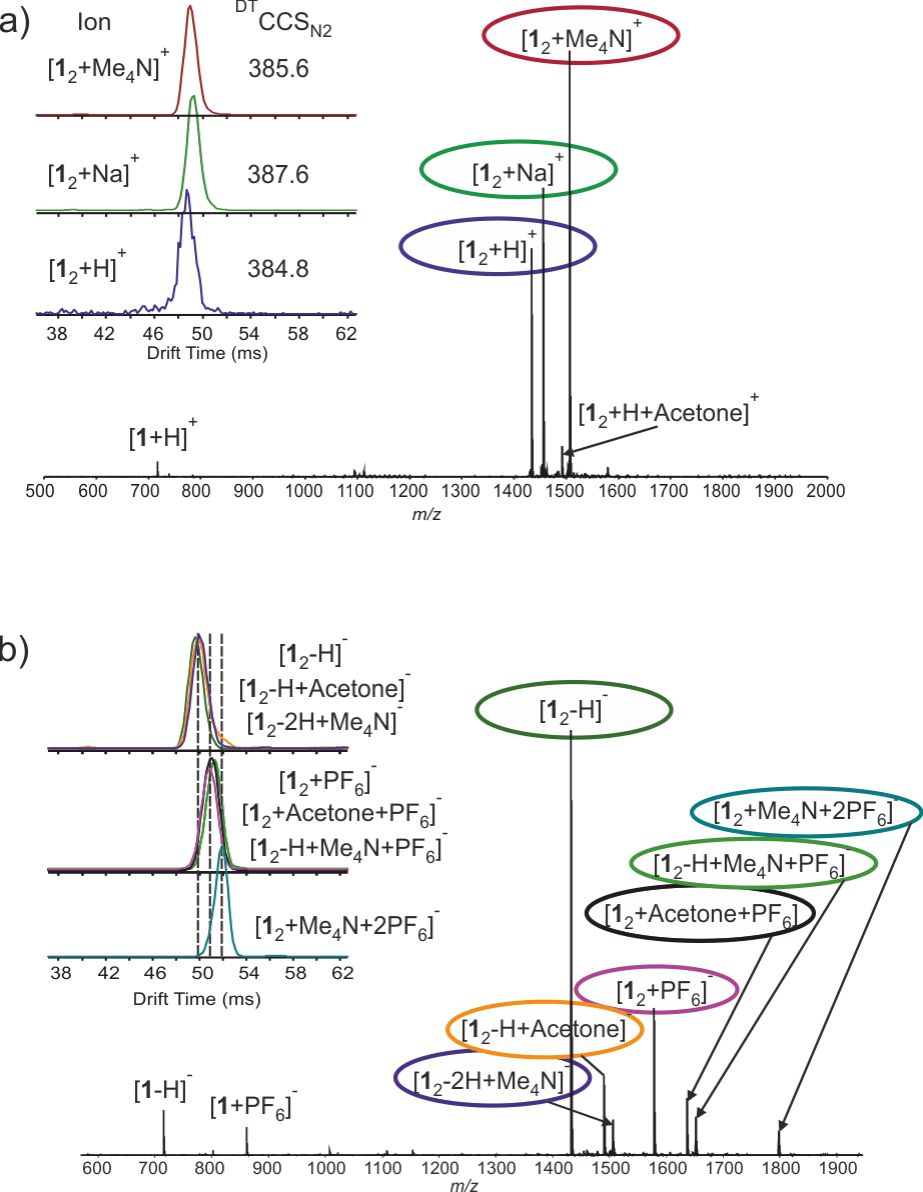
Spectra of **1** + Me_4_NPF_6_ 1:3 in acetone in a) (+)ESI-MS and b) (−)ESI-MS. Insets showing arrival time distributions for selected ions. Identical drift times demonstrate the same structural diameters and CCS values for these ions. Separate arrival time distributions (ATDs) for ions in Figure 1b are shown in Supporting Information File 1 (Figure S2). *m*/*z* values, mass accuracies and CCS values are listed in Table S1 (Supporting Information File 1).

We have previously shown that tetraisobutylpyridine[4]arene forms hydrogen-bonded dimers with eight intermolecular N–H···O(amide) hydrogen bonds in the solid state, in solution and in the gas phase [11]. Resorcinarene capsules of similar size are well-known for their ability to encapsulate small alkylammonium cations inside the dimer, especially quaternary ammonium cations [12–14]. As the cavity sizes of both pyridinearene and resorcinarene dimers are comparable, alkylammonium cations were chosen as the guests for complexation studies with ESI-Q-TOF mass spectrometry. Complex formation was tested with the following series of cationic guests: MeNH_3_^+^, Me_2_NH_2_^+^, Me_3_NH^+^, Me_4_N^+^, EtNH_3_^+^, Et_2_NH_2_^+^, Et_4_N^+^ and Pr_4_N^+^, which were used as the corresponding Cl^−^ or Br^−^ salts. None of the cations MeNH_3_^+^, Me_2_NH_2_^+^, Me_3_NH^+^, EtNH_3_^+^, Et_2_NH_2_^+^ or Pr_4_N^+^ formed complexes with **1** and **1**_2_, neither using electrospray ionization (ESI) in the positive nor the negative mode. Pr_4_N^+^ is certainly too large to fit inside the dimeric capsule, whereas the other non-complexing cations are protic and – as rather strong hydrogen-bond donors – may interfere with intermolecular hydrogen bonds of the dimer. Smaller quaternary cations Me_4_N^+^ and Et_4_N^+^ were observed to form 1:1 and 2:1 host–guest complexes. With Me_4_N^+^, [**1**_2_ + Me_4_N]^+^ was observed in the positive mode, and ions [**1** − 2H + Me_4_N]^−^ and [**1**_2_ − 2H + Me_4_N]^−^ in the negative mode (Figure 1). Complexation of Et_4_N^+^ was observed only in (−)ESIMS as [**1** − 2H + Et_4_N]^−^ and [**1**_2_ − 2H + Et_4_N]^−^ ions. With the chloride and bromide salts, the abundance of cation complexes was modest, but it significantly increased, in both the positive and negative modes, when the counterion was changed to PF_6_^−^, BF_4_^−^ or I^−^, which have previously been observed to form *exo* complexes with pyridinearenes [8]. In addition, ternary ion pair complexes such as [**1**_2_ + Me_4_N + 2A]^−^ and [**1**_2_ – H + Me_4_N + A]^−^ (A = anion, i.e., PF_6_^−^, BF_4_^−^, or I^−^) were detected in the negative mode.

The location (*endo* or *exo*) of the cation is of structural interest and of vital importance to understand the supramolecular chemistry of pyridinearenes. Interestingly, ternary complexes of **1_2_** containing both cation and solvent are not observed in either of the ESI-MS modes, even though in earlier studies such complexes were observed with solvent and anion [11]. This points to an *endo* location of the cation. Ion mobility mass spectrometry (IM-MS) is a powerful tool to study structural features such as the *endo*/*exo* complexation of supramolecular complexes [11,15–18]. Drift tube ion mobility mass spectrometry (DT-IMMS) enables the determination of structure-related collision cross section (CCS) values without complicated calibration. As depicted in Figure 1, DT-IM-MS revealed that drift times for the ions [**1**_2_ + H]^+^, [**1**_2_ + Me_4_N]^+^ and [**1**_2_ + Na]^+^ are very similar. In fact, the obtained ^DT^CCS_N2_ [19] value for [**1**_2_ + Me_4_N]^+^ (385.6 ± 0.40 Å^2^) is, within the error, the same as for [**1**_2_ + H]^+^ (384.8 ± 0.41 Å^2^) and smaller than for the [**1**_2_ + Na]^+^ complex, thus indicating inclusion complexation. This clearly indicates that the Me_4_N^+^ complex of **1**_2_ has the same rotation average diameter as the protonated dimer and thus the cation is encapsulated inside the cavity. Calculated [20–21] theoretical ^DTM^CCS_N2_ [22] values indicate that an *exo*-complexation of the cation should result in a ca. 10 Å^2^ larger CCS (415.6 Å^2^ vs 405.7 Å^2^) value as compared to the *endo*-complex. Ions [**1**_2_ − H]^−^ (^DT^CCS_N2_ = 387.0 ± 0.44 Å^2^) and [**1**_2_ − 2H + Me_4_N]^−^ (^DT^CCS_N2_ = 389.9 ± 0.41 Å^2^) also exhibit effectively the same ^DT^CCS_N2_ values and show similar drift times (Figure 1 and Figure S2, Table S1, Supporting Information File 1) in their arrival time distributions (ATDs). Ions [**1**_2_ + PF_6_]^−^ and [**1**_2_ – H + Me_4_N + PF_6_]^−^ that presumably carry one *exo*-complexed anion result in ca. 9 Å^2^ larger ^DT^CCS_N2_ values of 395.9 ± 0.50 Å^2^ and 399.6 ± 0.49 Å^2^. For [**1**_2_ + Me_4_N + 2PF_6_]^−^ with two attached counter ions, the ^DT^CCS_N2_ value is even larger (5–8 Å^2^ larger compared to ions with one anion). This clearly demonstrates the anions to be located in the outer periphery, while the cation is encapsulated inside the dimer.

The difference in *endo/exo* complexation is clear in IM-MS, when the larger PF_6_^−^ anion is used in the experiment. For complexes with smaller anions BF_4_^−^ and I^−^ the structural conclusions are more difficult to draw based only on CCS values, as the difference between the *endo* and *exo* complexes is smaller. To obtain complementary information, the ternary complex ions [**1**_2_ + Me_4_N + 2A]^−^ (A = PF_6_^−^, BF_4_^−^, I^−^) were further investigated by infrared multiphoton dissociation (IRMPD). IRMPD is an MS/MS technique, which can be utilized to study the fragmentation of supramolecular complexes [23]. In these experiments, the [**1**_2_ + Me_4_N + 2A]^−^ ions were mass-selected and irradiated for 20 to 250 ms with a laser power of 95% of 25 W. At short irradiation times, the main dissociation product was [**1**_2_ + Me_4_N − 2H]^−^, which is produced after elimination of two molecules of HA (HA = H^+^A^−^, Schematic presentation of dissociation in Figure S4, Supporting Information File 1). This ion further dissociates to a neutral monomer and to the [**1** − 2H + Me_4_N]^−^ ion, which can only result from two HA eliminations and dissociation of the H-bonded capsule. Elimination of two HA molecules and formation of [**1** + Me_4_N − 2H]^−^ would be unexpected from a complex with *exo*-complexed cation. Minor fragments resulting from a direct elimination of an ion pair are observed and [**1**_2_ + A]^−^ appears in spectra. This likely results from a relocation of the anion to a position close to the hydrogen-bonded seam of the dimer, which is then partially opened to release the ion pair (pathway 2 in Figure S4, Supporting Information File 1). As all studied ternary complexes behaved similarly in the experiments, it can be stated that Me_4_N^+^ is located inside the cavity, while the PF_6_^−^, BF_4_^−^ and I^−^ anions reside in the *exo*-binding site.

**Figure 2:**
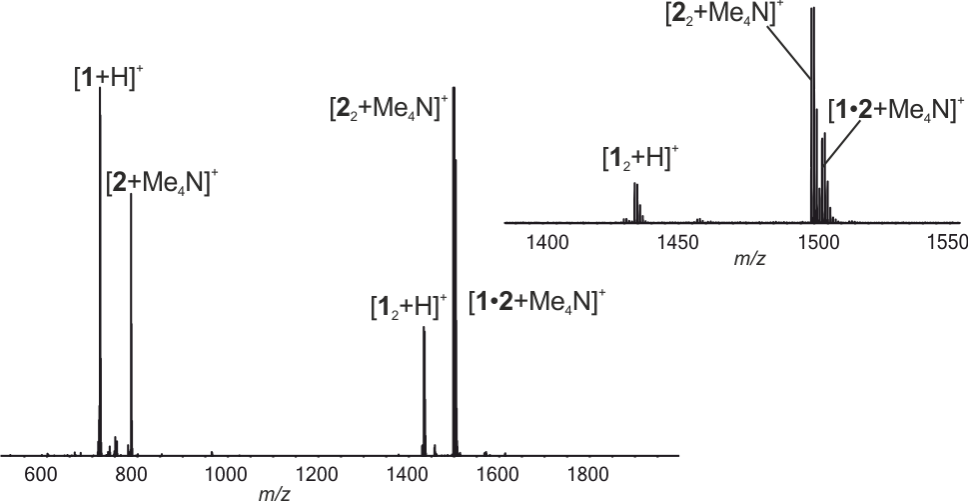
(+)ESI-MS profile spectrum of the mixture of **1**, **2** and TMAPF_6_ in acetonitrile (20 µM, 1:1:1). Inset shows a zoom in for region showing dimeric ions.

The binding efficiency of pyridine[4]arene towards the Me_4_N^+^ cation was compared to that of resorcin[4]arene, which is known to bind small cations with high affinity. **1**, **2** and Me_4_NPF_6_ were mixed in 1:1:1 ratio and measured by ESI-QTOF-MS. As seen in Figure 2, the resorcin[4]arene dimer [**2**_2_ + Me_4_N]^+^ was detected as the base peak in the positive mode, while [**1**_2_ + Me_4_N]^+^ was hardly observed. In addition to the pure dimers, the formation of the heterodimer [**1**·**2** + Me_4_N]^+^ was also observed. In the negative mode, heterodimers [**1**·**2** − H]^−^ and [**1**·**2** − 2H + Me_4_N]^−^ were observed with higher intensity than the corresponding homodimeric capsules of **1** or **2** (Figure S1, Supporting Information File 1). This is surprising due to differences in H-bonding geometry between dimers of **1** or **2**. Previously in case of hexameric capsules heterohexamers of pyridinearene and resorcinarene were not observed [5]. The PF_6_^−^ anion was observed to be bound to the pyridine[4]arene dimer in [**1**_2_ + PF_6_]^−^ and the heterodimer [**1**·**2** + PF_6_]^−^, but interestingly the abundance of [**1**·**2** + PF_6_]^−^ was half of [**1**_2_ + PF_6_]^−^, and PF_6_^−^ complexes with **2** or **2**_2_ had even lower abundances. This clearly shows that the anion has a higher affinity to pyridinearene than to resorcinarene. Inclusion complexation of solvent was also only detected with the pyridinearene homodimer (for example as the [**1**_2_ + MeCN + PF_6_]^−^ ion). This mixed host experiment shows the resorcin[4]arene affinity towards Me_4_N^+^ to be higher than that of pyridine[4]arene. Also, the lack of anion and/or solvent complex formation with **2** or **2**_2_ in the gas phase indicates that this binding feature is unique to pyridinearenes and it does not take place with resorcinarene. The high abundance of ternary ion pair complexes with pyridinearene, but not with resorcinarene indicates that the cation complexation process to pyridinearene is anion driven at least to some extent. The reduced electron density in the pyridine rings is compensated by favorable "through-wall" electrostatic interactions between the *exo*-anion(s) and the *endo*-cation.

In solution, by ^1^H NMR, complexation of Me_4_N^+^ cation was observed with **2**, but not with **1** (Figures S5 and S6, Supporting Information File 1). In a sample of 10 mM **2** and Me_4_NPF_6_ (1:3 ratio in CDCl_3_/CD_3_CN 4:3 v:v mixture) a 0.35 ppm upfield shift was observed for the Me_4_N^+^ signal. In a sample of **1** and Me_4_NPF_6_ no shift is observed. This clearly indicates that also in solution **1** does not possess a similar affinity towards the Me_4_N^+^ cation as **2**. Also, all our attempts to obtain a solid-state single crystal structure of **1** with a cationic guest were unsuccessful. It is possible that the observation of such complexes requires special conditions present in the ESI source.

To obtain more detailed insights into the observed gas-phase structures, DFT calculations were carried out for **1**, **1_OH_**, **1**_2_, [**1**_2_ + Me_4_N_endo_]^+^, [**1**_2_ + Me_4_N_exo1_]^+^, [**1**_2_ + Me_4_N_exo2_]^+^, [**1**_2_ + Me_4_N_endo_ + I_endo_]_,_ [**1**_2_ + Me_4_N_endo_ + I_exo_]_,_ [**1**_2_ + Me_4_N_endo_ + 2I_exo_]^−^_,_ [**1**_2_ + I_exo_]^−^_,_ and [**1**_2_ + 2I_exo_]^2−^(Figure S7, Supporting Information File 1) without (PBE0/def2-TZVP) and with (PBE0-D3/def2-TZVP) dispersion correction because in the gas phase dispersion plays an important role in the formation of supramolecular complexes [24–30]. Conformational analysis was first performed for both tautomers of the monomer to ensure that further calculations were carried out for the most stable tautomer. The conformational analysis revealed that the pyridone tautomer (**1**) is ≈150 kJ/mol lower in energy than the dihydroxy tautomer (**1_OH_**). The geometry optimizations showed that the geometry of [**1**_2_ + Me_4_N_endo_]^+^ is similar without and with dispersion correction (hydrogen bond lengths are listed in Table S2, Supporting Information File 1). This is expected, because the standard hybrid functionals are able to describe strong hydrogen bonds such as NH···O and OH···O in a reasonable manner [31–32]. Due to the encapsulated guest molecule, the [**1**_2_ + Me_4_N_endo_]^+^ complex has slightly longer NH···O and OH···O bonds than empty **1**_2_. The addition of I^−^ anion(s) at the lower rim(s) of [**1**_2_ + Me_4_N_endo_]^+^ has hardly any influence on the length of NH···O and OH···O bonds as exemplified by the hydrogen bond lengths of [**1**_2_ + Me_4_N_endo_ + I_exo_] and [**1**_2_ + Me_4_N_endo_ + 2I_exo_]^−^. The same trend is also observed for cation-free complexes ([**1**_2_ + I_exo_]^−^ and [**1**_2_ + 2I_exo_]^2−^) if their NH···O and OH···O bond lengths are compared to those in **1**_2_.

Calculations were also carried out for complexes, where *exo* binding of the cation was considered. The geometry optimization yields two different geometries, [**1**_2_ + Me_4_N_exo1_]^+^ and [**1**_2_ + Me_4_N_exo2_]^+^ (Figure S7, Supporting Information File 1). In [**1**_2_ + Me_4_N_exo1_]^+^, the Me_4_N^+^ cation interacts with the lower rim isobutyl groups only when the dispersion interaction is included in calculations, whereas at the PBE0/def2-TZVP level [**1**_2_ + Me_4_N_exo1_]^+^ is not a stable minimum on the potential energy surface and results in an additional *exo* complex, namely [**1**_2_ + Me_4_N_exo2_]^+^, in which the Me_4_N^+^ cation resides in proximity to the seam of hydrogen bonds. The NH···O and OH···O bond distances for [**1**_2_ + Me_4_N_exo1_]^+^ are almost identical with **1**_2_ indicating that the coordination of the Me_4_N^+^ cation at the lower rim has only a minor effect on the hydrogen-bond network of **1**_2_. To verify complexation of separate ion pairs, the geometry optimization was carried out also for [**1**_2_ + Me_4_N_endo_ + I_endo_] _,_ which showed that the cavity of **1**_2_ is too small for the simultaneous complexation of anionic and cationic guests, resulting in a partial rupture of the hydrogen-bonding seam and a ≈100 kJ/mol weaker interaction energy compared to [**1**_2_ + Me_4_N_endo_ + I_exo_] (Figure S7 and Table S2, Supporting Information File 1).

**Figure 3:**
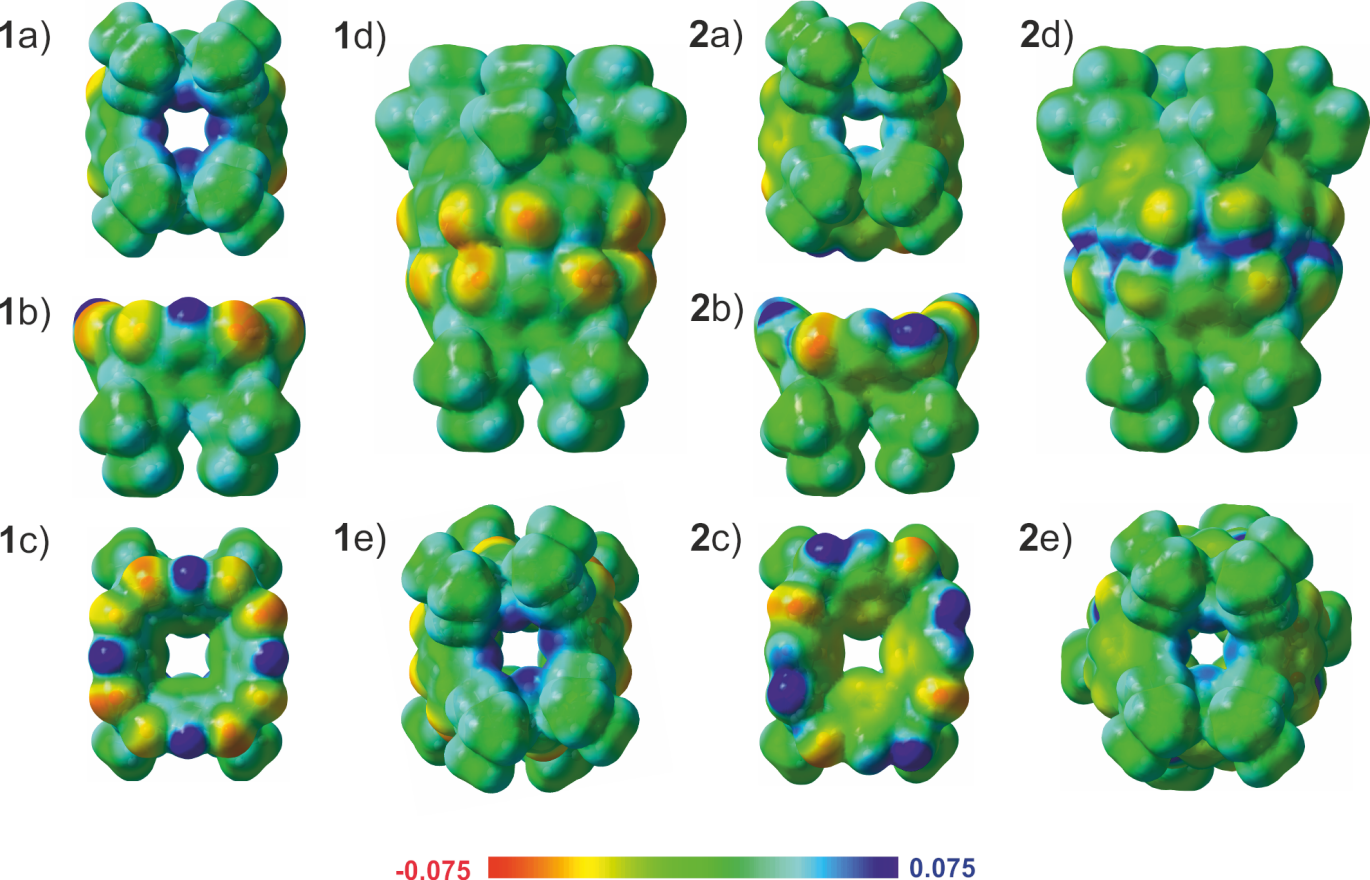
Calculated ESP surfaces (in au) superimposed on the total electron density (0.004 au) for **1** and **2**: a) monomer, bottom view b) monomer, side view c) monomer, top view, d) dimer, side view and e) dimer, bottom view. Red, blue and green surfaces indicate negative, positive and neutral ESP, respectively.

To illustrate the unsuitability of the lower rim for binding cationic guests, we mapped the ESP surfaces for **1**, **1**_2_, [**1**_2_ + Me_4_N_endo_]^+^, [**1**_2_ + Me_4_N_exo1_]^+^, and [**1**_2_ + Me_4_N_exo2_]^+^. Although the ESP surface of **1** show some π-acidic character for the cavity, as depicted in Figure 3, the electron-poor areas of **1**_2_, [**1**_2_ + Me_4_N_endo_]^+^, [**1**_2_ + Me_4_N_exo1_]^+^, and [**1**_2_ + Me_4_N_exo2_]^+^ (see also Figures S9 and S10, Supporting Information File 1) are more concentrated on the lower rim isobutyl groups than on the seam of the hydrogen bonds that actually contain electron-rich oxygen atoms able to interact with the Me_4_N^+^ cation through ion–dipole interactions. In the gas phase and in the absence of the dispersion force, the positively charged Me_4_N^+^ cation, therefore, prefers interactions with the electron-rich oxygen atoms of the hydrogen-bond network to the positively charged lower rim isobutyl groups.

The calculated interaction energies are listed in Table S2 (Supporting Information File 1). When the dispersion correction and/or the counterion(s) are taken into account, the energy analysis supports the findings obtained from ESI-MS studies, geometry optimizations and ESP surfaces: The Me_4_N^+^ cation is most likely bound inside the cavity of **1**_2_. It is also important to note that the calculated dispersion-corrected interaction energy of [**1**_2_ + Me_4_N_exo1_]^+^ (−37 kJ/mol) is roughly two fifth of the interaction energy of [**1**_2_ + Me_4_N_exo2_]^+^ (−98 kJ/mol) because there is no favorable ion–dipole interaction between **1**_2_ and Me_4_N^+^ in [**1**_2_ + Me_4_N_exo1_]^+^. Even though the encapsulation of the Me_4_N^+^ cation is a favorable process already even without anions, the calculated interaction energies show that the complexation of cationic guest can be enhanced by the additional *exo*-complexation of anions to the lower rims. The interaction energies of [**1**_2_ + Me_4_N_endo_]^+^, [**1**_2_ + Me_4_N_endo_ + I_exo_]_,_ and [**1**_2_ + Me_4_N_endo_ + 2I_exo_]^−^_,_ increases from −152 kJ/mol to −342 kJ/mol, and −521 kJ/mol, respectively, when the amount of I^−^ anions is increased in the complex. Moreover, the calculated interaction energies illustrate well that the dispersion interaction has considerable influence on the interaction energies of [**1**_2_ + Me_4_N_endo_]^+^ (−126.4 kJ/mol), [**1**_2_ + Me_4_N_exo1_]^+^ (−37.5 kJ/mol), [**1**_2_ + Me_4_N_exo2_]^+^ (−23.2 kJ/mol), [**1**_2_ + Me_4_N_endo_ + I_exo_] (−130.5 kJ/mol), and [**1**_2_ + Me_4_N_endo_ + 2I_exo_]^−^ (−133.7 kJ/mol), although their geometries are similar at both levels of theory. This means that pyridinearene dimers and their complexes are already sufficiently large supramolecular systems in which the omnipresent dispersion interaction can add up to a substantial force due to multiple interaction sites.

Calculations were also carried out for **2**, **2**_2_, [**2**_2_ + Me_4_N_endo_]^+^, [**2**_2_ + I_exo_]^−^_,_ and [**2**_2_ + 2I_exo_]^2−^, at the PBE0-D3/def2-TZVP level of theory (Figure S8, Supporting Information File 1) to explain the observed differences between **1** and **2** in the experimental ESI-MS studies. If the interaction energies of [**2**_2_ + Me_4_N_endo_]^+^ (−220 kJ/mol) and [**1**_2_ + Me_4_N_endo_]^+^ (−152 kJ/mol) are compared it is evident that **2**_2_ is a better host for the Me_4_N^+^ cation than **1**_2_. On the other hand, when comparing the interaction energies of complexes [**2**_2_ + I_exo_]^−^ (−149 kJ/mol), [**2**_2_ + 2I_exo_]^2−^ (−170 kJ/mol), [**1**_2_ + I_exo_]^−^ (−175 kJ/mol), and [**1**_2_ + 2I_exo_]^2−^ (−223 kJ/mol), it can be stated that **1**_2_ has a stronger affinity towards anions than **2**_2_. These results are fully in line with the MS studies and calculated ESP surfaces. By comparing the calculated ESP surfaces of **1** and **2** in Figure 3, it is a clear that the π-basic character of the cavity is more pronounced in **2** than in **1**. The investigation of ESP surfaces of **1**, **1**_2_, **2**, and **2**_2_ also reveal that the lower rims of pyridine[4]arenes **1** and **1**_2_ are more electron deficient than **2** and **2**_2_, explaining the weaker affinity of **2** and **2**_2_ towards anions. This result is also underpinned by the calculated interaction energies of anionic complexes; −149 kJ/mol ([**2**_2_ + I_exo_]^−^) vs −175 kJ/mol ([**1**_2_ + I_exo_]^−^) and −170 kJ/mol ([**2**_2_ + 2I_exo_]^2−^) vs −223 kJ/mol ([**1**_2_ + 2I_exo_]^2−^) that show a stronger affinity of **1**_2_ towards anions compared to **2**_2_.

## Conclusion

In conclusion, we have shown that the pyridine[4]arene dimer, which until recently was considered to be an anion receptor, can bind also cationic guests. The Me_4_N^+^ cation was observed to bind to the pyridine[4]arene dimer, and all mass spectrometric data and theoretical calculations show undoubtedly that the cation is located inside the cavity of the dimer in the gas phase. It is an interesting fact, that the binding properties of pyridine[4]arene differ from earlier reports [7–8]. However, cation binding to pyridinearene is clearly not as strong as with resorcin[4]arene, which is known for its excellent cation receptor properties. A comparison of these two macrocyclic hosts reveals significant differences in their binding properties. Pyridine[4]arene appears to have a better affinity towards neutral guests and has also a higher affinity to complex anions at the *exo*-binding sites at the lower rim, whereas resorcin[4]arene clearly has a higher affinity towards cations. However, the pyridinearenes' ability to form *exo*-complexes with anions can assist its ability to bind cations in *endo*-positions.

## Experimental

Compounds **1** and **2** have been prepared according to the reported procedures [11,33–34]. Salts were commercially available (From Aldrich, Fluka and TCI) and used as received. Mass spectrometric experiments have been performed with an ABSciex QSTAR Elite ESI-Q-TOF mass spectrometer. Ion mobility mass spectrometry (IM-MS) experiments were conducted with a Waters Synapt G2 equipped with a linear drift cell and Agilent 6560 Ion mobility Q-TOF mass spectrometer. All CCS values were obtained using nitrogen as a drift gas and stepped field methods. IRMPD experiments were performed with an Ionspec QFT-7 ESI-FT-ICR with a 7 T superconducting magnet. Samples for all mass spectrometric experiments were prepared with 10 or 20 µM concentration and 1:3 host–guest ratio in acetone. Theoretical CCS values were calculated using IMoS [20–21]. NMR experiments were performed with a Bruker Avance III HD 300 NMR spectrometer. Samples were prepared at 10 mM concentration and 1:3 host–guest ratio in CDCl_3_/CD_3_CN (4:3, v:v) mixture. DFT calculations were performed by Spartan’ 16 and Gaussian 09 (D01) software packages. More detailed information of the experiments and parametrization can be found in Supporting Information File 1.

## Supporting Information

File 1 Experimental details and supplementary information.
