# Supplementary material for: Anion-driven encapsulation of cationic guests inside pyridine[4]arene dimers
Source: Beilstein J Org Chem. 2019 Oct 21;15:2486–92. doi: 10.3762/bjoc.15.241 (PMC6839556; doi:10.3762/bjoc.15.241)
Supplement: File 1 — Experimental details and supplementary information. [file Beilstein_J_Org_Chem-15-2486-s001.pdf]

BEILSTEIN JOURNAL OF ORGANIC CHEMISTRY

Supporting Information

for

Anion-driven encapsulation of cationic guests inside pyridine[4]arene dimers

Anniina Kiesilä, Jani O. Moilanen, Anneli Kruve, Christoph A. Schalley, Perdita Barran and Elina Kalenius

Beilstein J. Org. Chem. **2019,** *15,* 2486–2492. doi:10.3762/bjoc.15.241

Experimental details and supplementary information

License and Terms: This is a supporting information file under the terms of the Creative Commons Attribution License (http://creativecommons.org/licenses/by/4.0). Please note that the reuse, redistribution and reproduction in particular requires that the authors and source are credited.
The license is subject to the *Beilstein Journal of Organic Chemistry* terms and conditions: (https://www.beilstein-journals.org/bjoc)

Table of Contents

1 Mass spectrometry S2

1.1 ESI-Q-TOF mass spectrometry S2

1.2 Ion mobility mass spectrometry S2

1.3 IRMPD experiments S4

2 NMR S6

3 Computational details S7

References S12

XYZ Coordinates S13

1 Mass spectrometry

1.1 ESI-Q-TOF mass spectrometry

Mass spectrometric experiments were performed with an ABSciex QSTAR Elite ESI-Q-TOF mass spectrometer, equipped with an API 200 TurboIonSpray ESI Source from AB Sciex. Nitrogen was used as the drying and nebulization gas. The parameters were optimized to get obtain the maximum abundance of the ions of interest. The measurements and data handling were done with Analyst® QS 2.0 software.

Complex formation was tested with a series of alkyl ammonium cations ($MeNH_3^+$, $Me_2NH_2^+$, Me_3NH^+, Me_4N^+, $EtNH_3^+$, $Et_2NH_2^+$, Et_4N^+ and Pr_4N^+) with Cl^-, Br^-, I^-, BF_4^- or PF_6^- as the counterions. All salts were commercially available and used as received. Stock solutions of salts were prepared at 5 mM in MeOH or MeCN. A 5 mM stock solution of host **1** was prepared in $CHCl_3$. Samples for profile spectra measurements were prepared with 20 µM host concentration and 1:3 host–guest ratio in acetone. From host **2**, a 5 mM stock solution was prepared in acetone. Mixed host experiments were carried out with 1:1:1 $host_1$/$host_2$/guest ratio (20 µM each) diluted in MeCN.

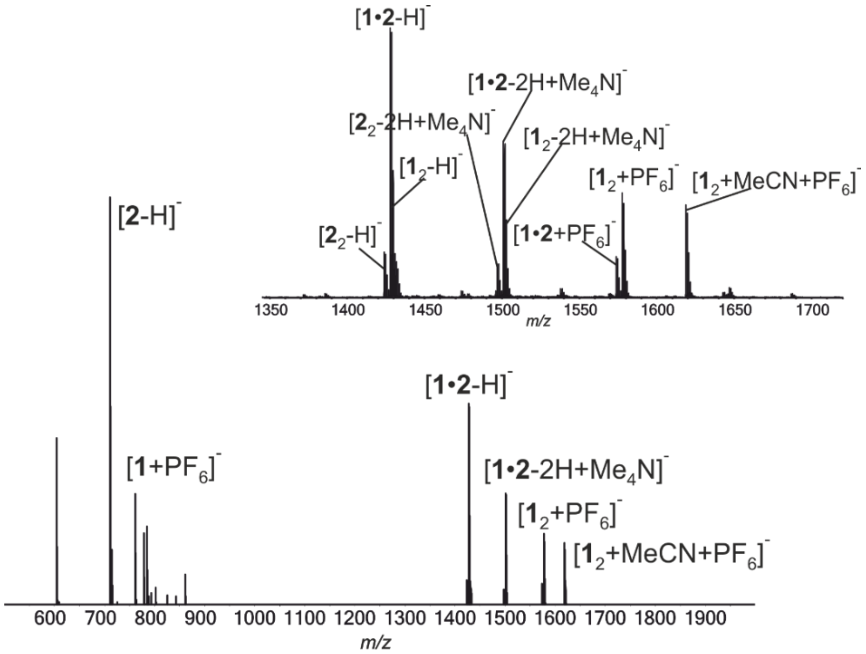

Figure S1. (−)ESIMS spectrum of the 1:1:1 mixture of **1**, **2**, and $TMAPF_6$ in acetonitrile. Inset shows a zoom in for the region showing dimeric ions.

1.2 Ion mobility mass spectrometry

Ion mobility mass spectrometry experiments were performed with Waters Synapt G2 and Agilent 6560 Ion mobility Q-TOF mass spectrometers. All CCS values were obtained using nitrogen as drift gas and stepped -field methods. Samples were prepared with 10 or 20 µM concentration and 1:3 host–guest ratio in acetone.

The modified Waters Synapt G2 was equipped with a linear drift cell, which was filled with ~2 Torr nitrogen (298 K). Samples were ionized with a nanoESI ion source. In-house pulled capillary tips were used with inserted platinum wire for nanoESI. Parameters were optimized as follows: Capillary voltage 2.01 kV, cone 50 V and source temperature 40 ºC. Data was analyzed using MassLynx v4.1 (Waters Corporation, USA) and Microsoft Excel 2016 (Microsoft, USA).

The Agilent 6560 IM Q-TOF instrument was equipped with an ESI ion source and samples were injected with a flowrate of 5 µL/min. The drift tube pressure was set at 3.95 Torr. The drift tube entrance and exit voltages were set to 1700 V and 224 V, respectively. The trap filling time was set to 20 000 µs and the trap release time to 150 µs. Other parameters were: gas temperature 275 ºC, dry gas flow rate 5 L/min, nebulizer pressure 10 psi, and capillary voltage of −5000 V or 5000 V. Before introducing the samples, ES tuning mix (Agilent Technologies) was measured as a reference for CCS values and to ensure stabile conditions for CCS determination [1,2]. For stepped-field measurements, the drift tube entrance voltage

was varied from 1100 V to 1700 V with 100 V increments. The data were analyzed using the MassHunter IM-MS Browser (Version B.08.00, Agilent Technologies, USA).

Theoretical CCS values were calculated with IMoS [3]. Theoretical $^{DTM}CCS_{N2}$ values were obtained using the trajectory method with diffuse scattering in nitrogen gas. Coordinates for calculations were obtained from DFT-calculated structures optimized at the PBE0-D3/def2-TZVP level of theory (for DFT calculations see section 3). The number of rotations was 3 with 300000 gas molecules per rotations. Calculations were carried out using experimental parameters (gas, temperature and pressure).

Table S1. Observed ions, their mass accuracies and CCS values. Theoretical $^{DTM}CCS_{N2}$ values shown for ions: $[1_2+Me_4N_{endo}]^+$, $[1_2+PF_{6exo}]^-$, $[1_2+Acetone_{endo}+PF_{6exo}]^-$. * Obtained theoretical $^{DTM}CCS_{N2}$ value for $[1_2+Me_4N_{exo1}]^+$ and $[1_2+Me_4N_{exo2}]^+$ are 415.6 Å^2 and 429.2 Å^2, respectively.

m/z	ion	Δ*m/z* (mDa)	experimental $^{DT}CCS_{N2}$ (Å^2)	theoretical $^{DTM}CCS_{N2}$ (Å^2)
717	$[1+H]^+$	0.39	268.9 ± 0.24	
739	$[1+Na]^+$	1.08	276.8 ± 0.25	
1433	$[1_2+H]^+$	-0.39	384.8 ± 0.41	
1455	$[1_2+Na]^+$	-0.65	387.6 ± 0.36	
1506	$[1_2+Me_4N]^+$	0.06	385.6 ± 0.40	405.7 *
715	$[1-H]^-$	-0.06	270.9 ± 0.32	
788	$[1-2H+Me_4N]^-$	-0.41	278.6 ± 0.22	
861	$[1+PF_6]^-$	-0.46	275.2 ± 0.35	
1431	$[1_2-H]^-$	-0.45	387.0 ± 0.44	
1489	$[1_2-H+Acetone]^-$	2.42	388.5 ± 0.47	
1504	$[1_2-2H+Me_4N]^-$	1.8	389.9 ± 0.41	
1577	$[1_2+PF_6]^-$	0.36	395.9 ± 0.50	415.4
1635	$[1_2+Acetone+PF_6]^-$	0.42	397.3 ± 0.49	421.5
1650	$[1_2-H+Me_4N+PF_6]^-$	0.41	399.6 ± 0.49	
1796	$[1_2+Me_4N+2PF_6]^-$	0.51	404.1 ± 0.52	

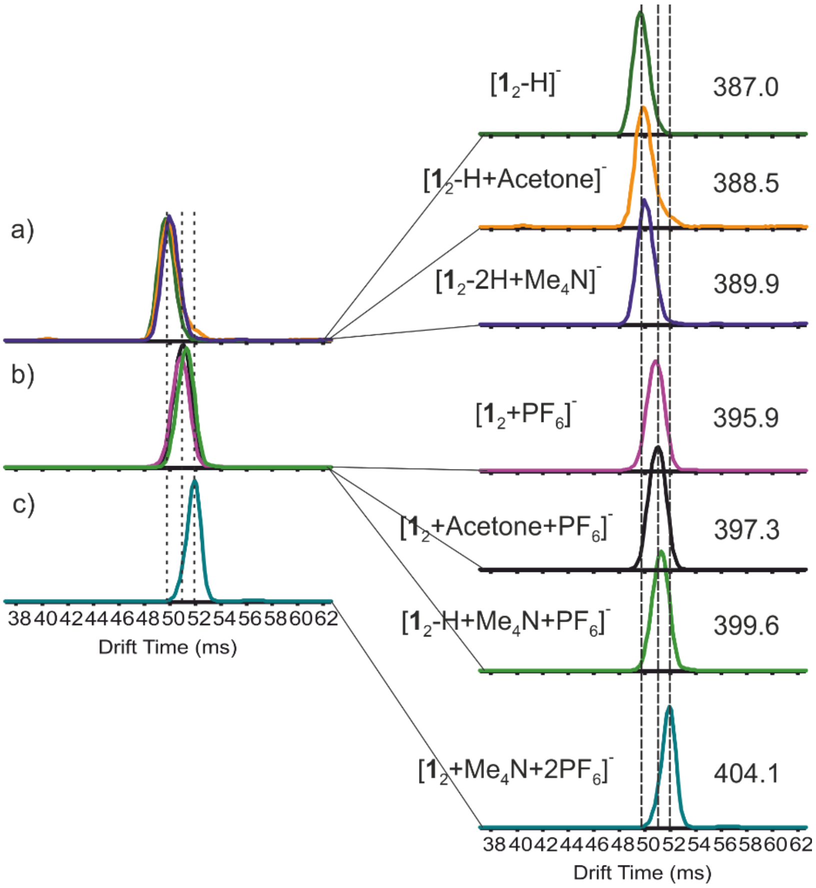

Figure S2. Arrival time distributions of the observed complexes in IM-MS experiments. In a) all the ions (empty dimer or dimers with *endo*-complexed guests) have the same drift times clearly evidencing that acetone and Me_4N^+ are located inside the cavity of the dimeric pyridine[4]arene capsules. Ions carrying one *exo*-complexed anion (b) exhibit longer drift times. The drift time increases again, when a second anion is attached to the periphery of the capsule (c).

1.3 IRMPD experiments

IRMPD experiments were performed with Ionspec QFT-7 ESI-FT-ICR (Varian Inc., Lake Forest, CA, USA), equipped with a 7 T superconducting magnet. Samples were prepared in a same way as in ESI-Q-TOF experiments. Samples were injected with a flow rate of 2 or 5 µL/min. A constant spray and highest intensities were achieved with a capillary voltage of 3500 V. The parameters for sample cone and extractor cone voltages were optimized for maximum abundances of the desired ion and were −60 and −10, respectively. The source temperature was 40 °C.

The ternary complexes [$\mathbf{1}_2$+Me_4N+2PF_6]$^-$, [$\mathbf{1}_2$+Me_4N+2BF_4]$^-$ and [$\mathbf{1}_2$+Me_4N+2I]$^-$ were mass-selected and irradiated with a laser power of 95% (from a CO_2 laser with 25 W maximum power) from 20 to 250 ms reaction intervals. All complexes showed similar dissociation behaviour as that of the hexafluorophosphate complex shown in Figure S3.

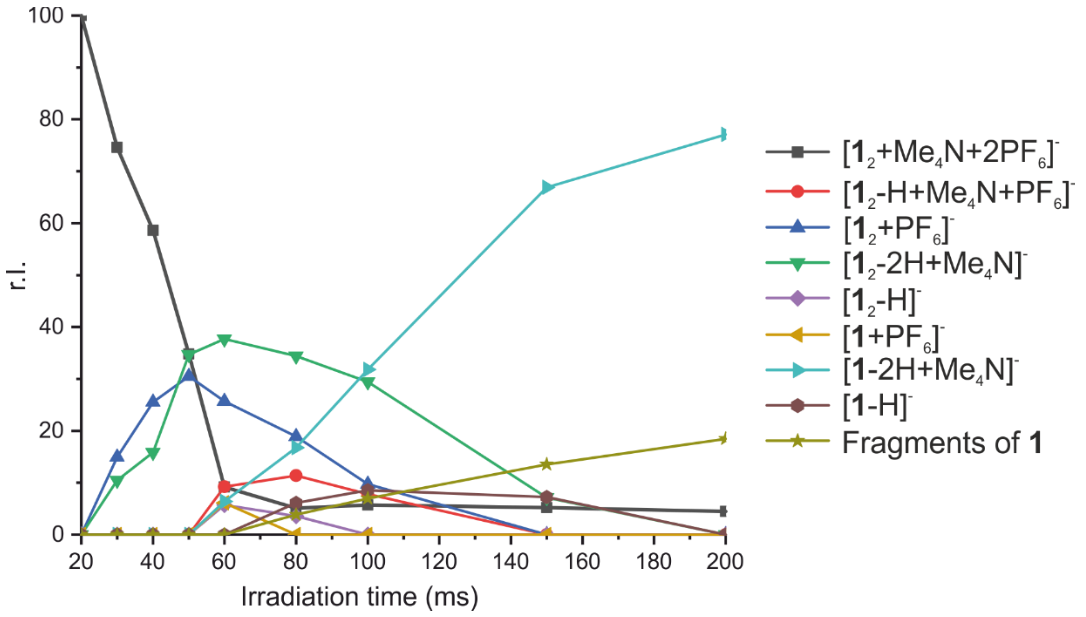

Figure S3. Dissociation of the ternary complex [$\mathbf{1}_2$+TMA+2PF$_6$]$^-$ in the IRMPD experiment with the intensity of the fragments plotted over the irradiation time.

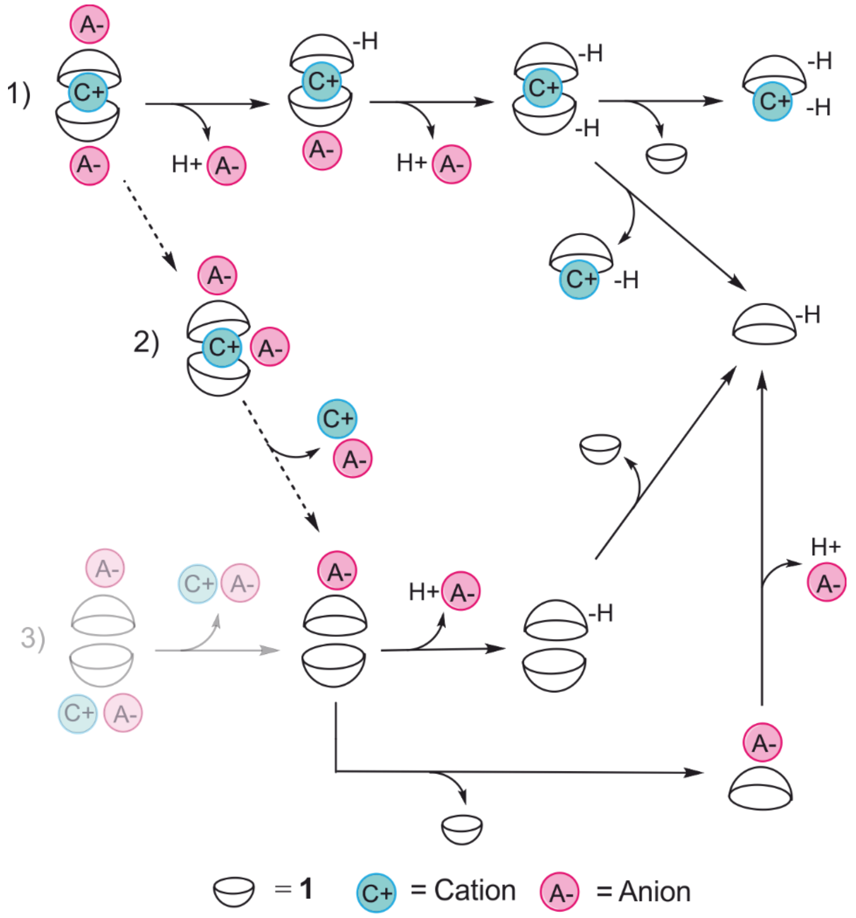

Figure S4. Schematic representation of the observed fragmentation pathways of mass-selected [$\mathbf{1}_2$+Me$_4$N+2A]$^-$ complexes in the IRMPD experiment. Pathway 1) was observed as the main route in line with the assignment of a cation-filled capsules carrying two external counterions.

2 NMR

^{1}H NMR spectra were measured with a Bruker Avance III HD 300 NMR spectrometer. Samples were prepared with 10 mM concentration and 1:3 host–guest ratio in $CDCl_3/CD_3CN$ (4:3, v:v) mixture and calibrated using CD_3CN signal at 1.95. ppm. Data was recorded at 298 K.

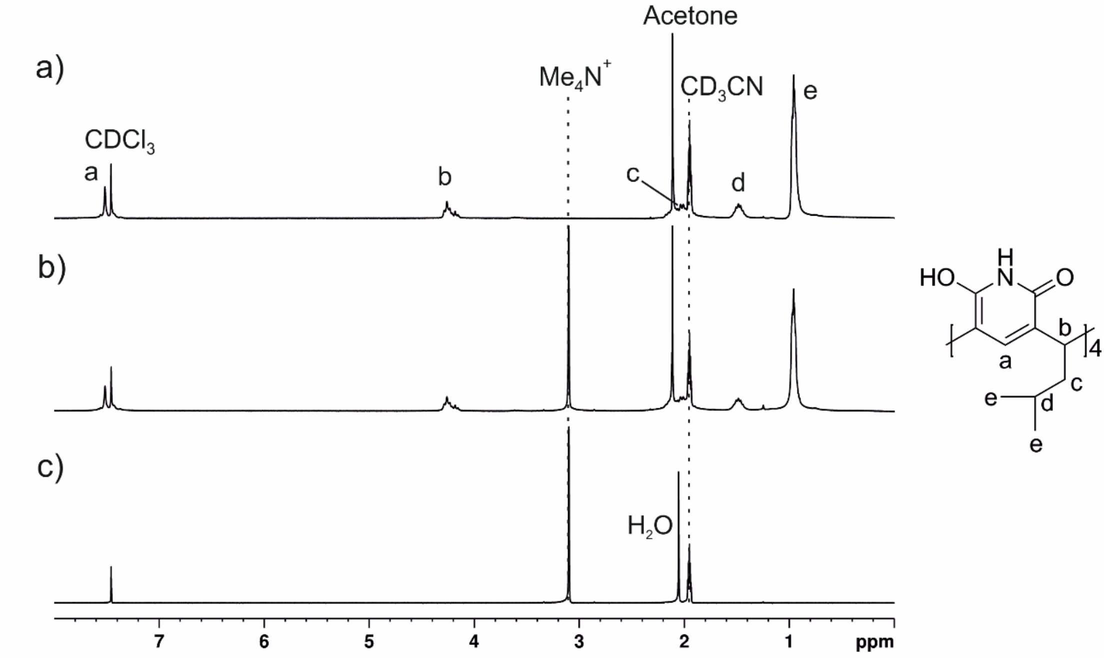

Figure S5. ^{1}H NMR spectrum of a) **1** b) **1** + Me_4NPF_6 (1:3 ratio) and c) Me_4NPF_6 in $CDCl_3/CD_3CN$ (4:3 v:v mixture) at 298 K. In b) no shift is observed in Me_4N signal, which indicates that **1** does not form a complex with Me_4N^+ in solution.

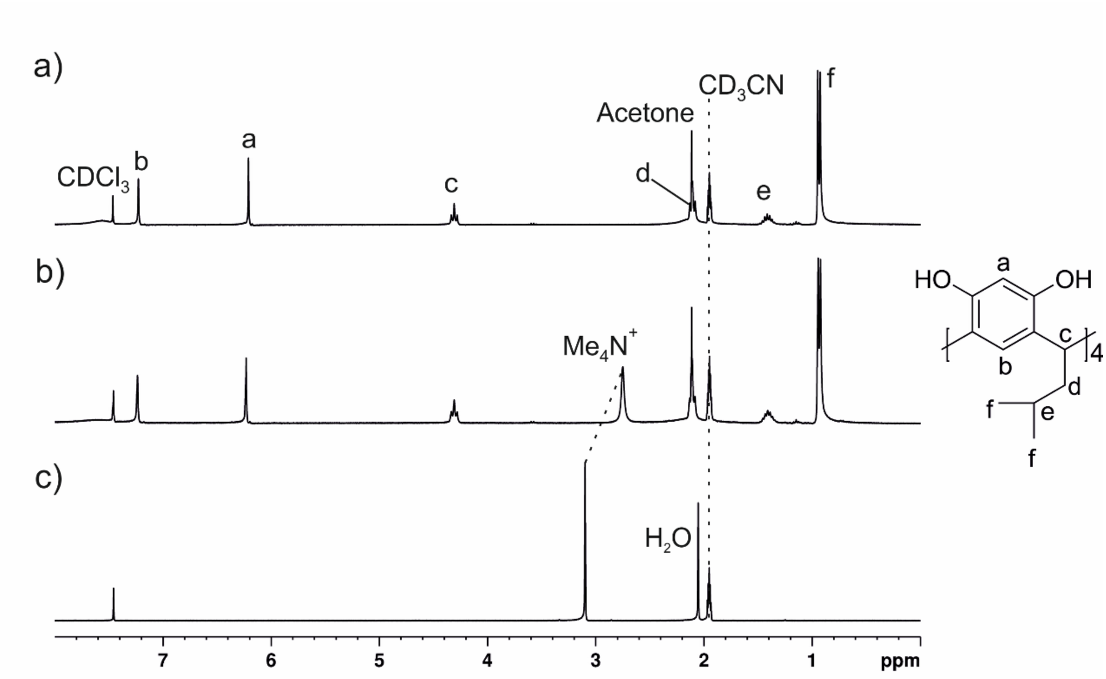

Figure S6. ^{1}H NMR spectrum of a) **2** b) **2** + Me_4NPF_6 (1:3 ratio) and c) Me_4NPF_6 in $CDCl_3/CD_3CN$ (4:3 v:v mixture) at 298 K. In b) the signal of Me_4N^+ shifts by 0.35 ppm, showing that **2** forms complex with Me_4N^+ in solution.

3 Computational details

The following computational procedure was employed to acquire the starting geometries for **1**$_2$, [**1**$_2$+Me_4N_{endo}]$^+$, [**1**$_2$+Me_4N_{exo1}]$^+$, [**1**$_2$+Me_4N_{exo2}]$^+$, [**1**$_2$+Me_4N_{endo}+I_{exo}], [**1**$_2$+Me_4N_{endo}+2I_{exo}]$^-$, [**1**$_2$+I_{exo}]$^-$, and [**1**$_2$+2I_{exo}]$^{2-}$ complexes. A systematic conformational sampling was first conducted for **1** and its hydroxy tautomer **1**$_{\mathbf{OH}}$ by using the Merck Molecular Force Field (MMFF) and rotating over all dihedral bonds [4]. PBE-D3/6-31+G** single-point energy calculations were performed for all unique structures (535 and 1248 in case of **1** and **1**$_{\mathbf{OH}}$, respectively) generated in the conformational sampling [5-9]. For **1** (**1**$_{\mathbf{OH}}$) single point energy calculations were followed by full geometry optimizations at the same level of theory for the 60 (53) lowest energy structures using the cut-off energy of 15 kJ/mol (20 kJ/mol). Of all the optimized structures, the 26 (29) lowest-energy structures were chosen employing the cut-off energy 10 kJ/mol (13 kJ/mol) for the final geometry optimizations that were carried out at the PBE0-D3/def2-TZVP level of theory [10-14]. The minimum structures of **1** and **1**$_{\mathbf{OH}}$ obtained from the above procedure are presented in Figure S10. As the hydroxy tautomer **1**$_{\mathbf{OH}}$ is 150 kJ/mol higher in energy than **1**, it can safely be assumed that the monomer exists mainly in the pyridone tautomer **1** in the gas phase. Thus, no further computational analysis was carried out for **1**$_{\mathbf{OH}}$ and its complexes. Assuming that **1**$_2$ maximizes intermolecular hydrogen bonds between two monomers in the gas phase and that the orientation of isobutyl groups is retained during the formation of **1**$_2$, good starting geometries for dimers **1**$_2$, [**1**$_2$+Me_4N_{endo}]$^+$, [**1**$_2$+Me_4N_{exo1}]$^+$, [**1**$_2$+Me_4N_{exo2}]$^+$, [**1**$_2$+Me_4N_{endo}+I_{exo}], [**1**$_2$+Me_4N_{endo}+2I_{exo}]$^-$, [**1**$_2$+I_{endo}]$^-$, and [**1**$_2$+2I_{exo}]$^{2-}$ complexes can be constructed from the minimum structures of **1**, Me_4N^+ and I^-. The geometries of these complexes were fully optimized at the PBE0-D3/def2-TZVP [10-14] and PBE0/def2-TZVP [10-12] levels of theory to investigate the influence of dispersion forces on the geometries and energies of the capsules under study. The starting geometries for **2** and **2**$_2$ was adapted from previous studies [15], whereas the starting geometries of [**2**$_2$+Me_4N_{endo}]$^+$, [**2**$_2$+I_{exo}]$^-$, and [**2**$_2$+2I_{exo}]$^{2-}$ where constructed in the similar manner than the starting geometries of complexes **1**$_2$. The geometries of **2** systems were only optimized at the PBE0-D3/def2-TZVP level of theory [10-14]. Due to the size of the investigated complexes, frequency analyses were only performed for the PBE0-D3/def2-SVP [10-12] optimized structures to ensure that the final structures correspond to local minima (no imaginary frequencies) on the potential energy hypersurface. For **1**$_{\mathbf{OH}}$ the PBE0-D3/def2-SVP level showed one small imaginary frequency (≈ -5 cm^{-1}) due the dispersion correction that was confirmed as an artefact at the PBE0/def2-SVP level of theory that showed no imaginary frequencies for **1**$_{\mathbf{OH}}$.

All calculations were performed by Spartan' 16 [16] and Gaussian 09 (D01) [17] software packages.

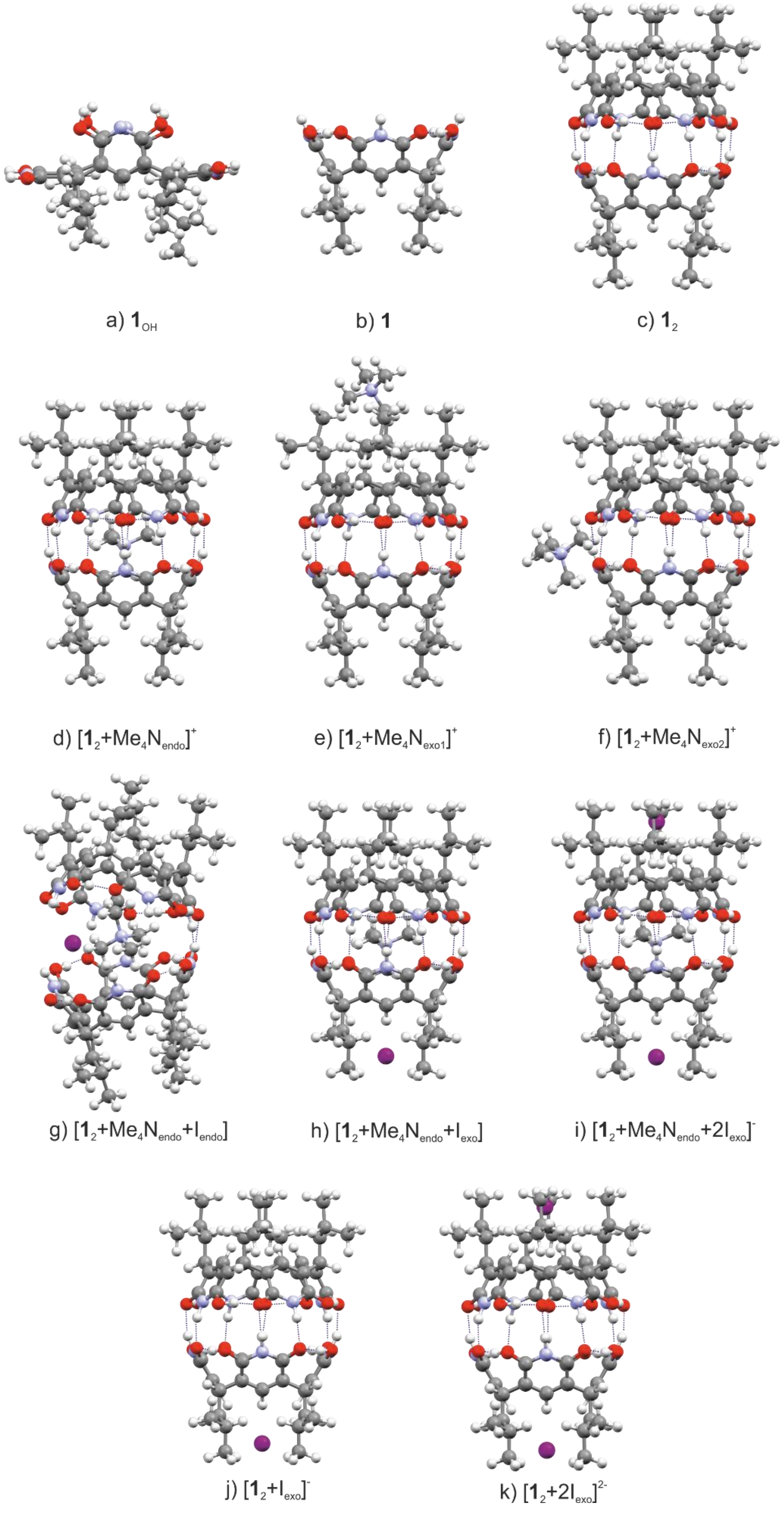

Figure S7. Optimized geometries for **1** at the PBE0-D3/def2-TZVP level of theory. Blue dotted lines represent H bonds. The PBE0/def2-TZVP optimized geometries are very similar.

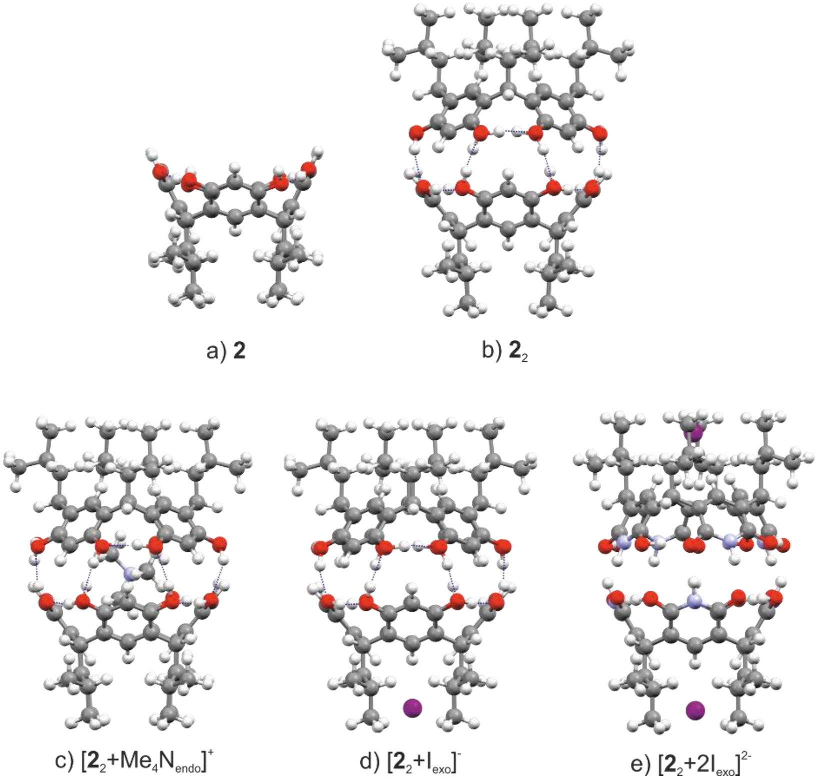

Figure S8. Optimized geometries for **2** at the PBE0-D3/def2-TZVP level of theory. Blue dotted lines represent H bonds. The PBE0/def2-TZVP optimized geometries are very similar.

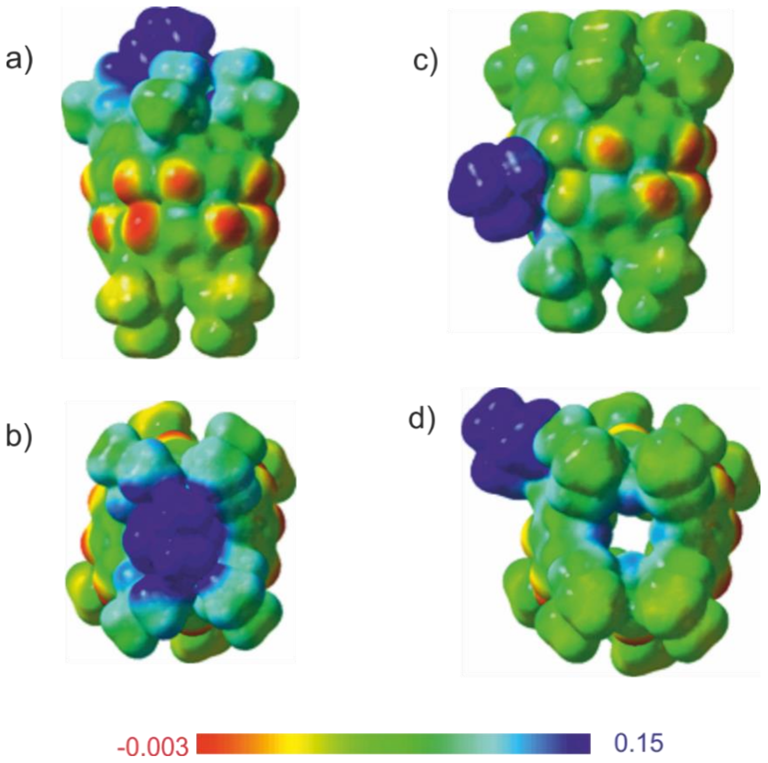

Figure S9. Calculated ESP surfaces (in au) superimposed on the total electron density (0.004 au) for a) [$\mathbf{1}_2$+Me_4N_{exo1}]$^+$ side view and b) [$\mathbf{1}_2$+Me_4N_{exo1}]$^+$ top view c) [$\mathbf{1}_2$+Me_4N_{exo2}]$^+$ side view and d) [$\mathbf{1}_2$+Me_4N_{exo2}]$^+$ top view. Red, blue and green surfaces indicate negative, positive and neutral ESP, respectively.

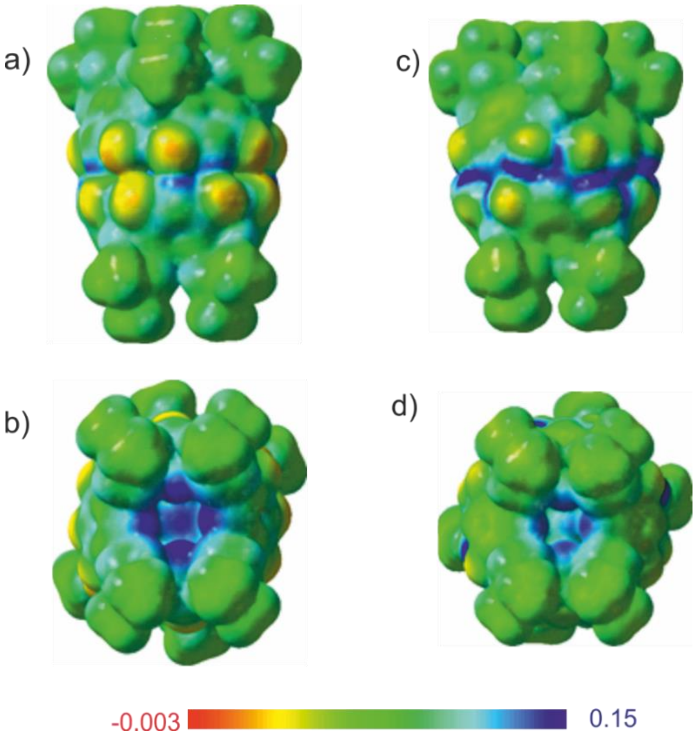

Figure S10. Calculated ESP surfaces (in au) superimposed on the total electron density (0.004 au) for a) [$\mathbf{1}_2$+Me_4N_{endo}]$^+$ side view b) [$\mathbf{1}_2$+Me_4N_{endo}]$^+$ bottom view c) [$\mathbf{2}_2$+Me_4N_{endo}]$^+$ side view and d) [$\mathbf{2}_2$+Me_4N_{endo}]$^+$ bottom view. Red, blue and green surfaces indicate negative, positive and neutral ESP, respectively.

Table S2. Optimized NH···O and NH···O bond lengths (Å) and calculated interaction energies (kJ/mol).

	NH···O		OH···O		E_{int}	
**Bond**	**PBE0-D3**	**PBE0**	**PBE0-D3**	**PBE0**	**PBE0-D3**	**PBE0**
$\mathbf{1}_2$	1.88-1.90	1.95-1.98	1.57-1.57	1.56-1.57	-200.15[b]	-125.82[b]
$[\mathbf{1}_2+Me_4N_{endo}]^+$	1.92-2.01	2.06-2.16	1.55-1.61	1.55-1.60	-152.14[c]	-25.70[c]
$[\mathbf{1}_2+Me_4N_{exo1}]^+$	1.85-1.93	-[a]	1.56-1.58	-[a]	-37.48[d]	-[a]
$[\mathbf{1}_2+Me_4N_{exo2}]^+$	1.85-1.92	1.93-1.99	1.46-1.64	1.46-1.63	-98.02[e]	-74.83[e]
$[\mathbf{1}_2+Me_4N_{endo}+I_{exo}]$	1.90-2.01	2.05-2.15	1.56-1.60	1.56-1.59	-342.05[f]	-211.54[f]
$[\mathbf{1}_2+Me_4N_{endo}+2I_{exo}]^-$	1.90-1.99	2.03-2.13	1.55-1.61	1.55-1.60	-521.33[g]	-387.64[g]
$[\mathbf{1}_2+Me_4N_{endo}+I_{endo}]$	1.76-5.64	1.82-6.33	1.42-1.72	1.43-1.69	-241.14[h]	-192.43[h]
$[\mathbf{1}_2+I_{exo}]^-$	1.83-1.93	1.89-2.01	1.57-1.58	1.56-1.57	-174.75[i]	-
$[\mathbf{1}_2+2I_{exo}]^{2-}$	1.88-2.00	1.95-1.98	1.57	1.56	-223.14[j]	-
$\mathbf{2}_2$	-	-	1.86-2.08	-	-206.91[b]	-
$[\mathbf{2}_2+Me_4N_{endo}]^+$	-	-	1.83-2.26	-	-219.52[c]	-
$[\mathbf{2}_2+I_{exo}]^-$	-	-	1.83-2.18	-	-148.85[i]	-
$[\mathbf{2}_2+2I_{exo}]^{2-}$	-	-	1.84-2.07	-	-170.19[j]	-

a) Not a stable minimum on the potential energy hypersurface.
b) $E_{int} = E[\mathbf{X}_2] - (2 \times E[\mathbf{X}])$, **X** = **1** or **2**
c) $E_{int} = E[\mathbf{X}_2+Me_4N_{endo}]^+ - (E[\mathbf{X}_2] + E[Me_4N]^+)$
d) $E_{int} = E[\mathbf{X}_2+Me_4N_{exo1}]^+ - (E[\mathbf{X}_2] + E[Me_4N]^+)$
e) $E_{int} = E[\mathbf{X}_2+Me_4N_{exo2}]^+ - (E[\mathbf{X}_2] + E[Me_4N]^+)$
f) $E_{int} = E[\mathbf{X}_2+Me_4N_{endo}+I_{exo}] - (E[\mathbf{X}_2+I_{exo}]^- + E[Me_4N]^+)$
g) $E_{int} = E[\mathbf{X}_2+Me_4N_{endo}+2I_{exo}]^- - (E[\mathbf{X}_2+2I_{exo}]^{2-} + E[Me_4N]^+)$
h) $E_{int} = [\mathbf{X}_2+Me_4N_{endo}+I_{endo}] - (E[\mathbf{X}_2+I_{exo}]^- + E[Me_4N]^+)$
i) $E_{int} = [\mathbf{X}_2+I_{exo}]^- - (E[\mathbf{X}_2] + I^-)$
j) $E_{int} = [\mathbf{X}_2+2I_{exo}]^{2-} - (E[\mathbf{X}_2] + 2I^-)$

References

1. Stow, S. M.; Causon, T. J.; Zheng, X.; Kurulugama, R. T.; Mairinger, T.; May, J. C.; Rennie, E. E.; Baker, E.S.; Smith, R. D.; McLean, J. A.; Hann S.; Fjeldsted, J. C. *Anal. Chem*. **2017**, *89*, 9048-9055.
2. Gabelica V. *et al*. *Mass Spectrom. Rev*. **2019**, *38*, 291-320.
3. Larriba C.; Hogan, C. J. *J. Comput. Phys*. **2013**, *251*, 344-36
4. Halgren, T. A. *J. Comput. Chem.* **1999**, *20*, 730-748.
5. Perdew, J. J.; Burke K.; Ernzerhof, M. *Phys. Rev. Lett.* **1996**, *77*, 3865-3868.
6. Perdew J. P.; Wang, Y. *Phys. Rev. B* **1992**, *45*, 13244-13249.
7. Clark, T.; Chandrasekhar, J.; Spitznagel G. W.; Schleyer, P. V. R. *J. Comput. Chem.* **1983**, *4*, 294-301.
8. Frisch, M. J.; Pople J. A.; Binkley, J. S. *J. Chem. Phys.* **1984**, *80*, 3265-3269.
9. Hehre, W. J.; Ditchfield R.; Pople, J. A. *J. Chem. Phys.* **1972**, *56*, 2257-2261.
10. Ernzerhof M.; Scuseria, G. E. *J. Chem. Phys.* **1999**, *110*, 5029-5036.
11. Adamo C.; Barone, V. *J. Chem. Phys.* **1999**, *110*, 6158-6170.
12. Weigend F.; Ahlrichs, R. *Phys. Chem. Chem. Phys.* **2005**, *7*, 3297-3305.
13. Grimme, S.; Ehrlich S.; Goerigk, L. *J. Comput. Chem.* **2011**, *32*, 1456-1465.
14. Grimme, S.; Antony, J.; Ehrlich S.; Krieg, H. *J. Chem. Phys.* **2010**, *132*, 154104.
15. Qi, Z.; Heinrich, T.; Moorthy, S.; Schalley, C. A. *Chem. Soc. Rev.* **2015**, *44*, 515-531.
16. Spartan 16; Wavefunction Inc.: Irvivine CA, **2016**.
17. Gaussian 09, Revision D.01, M. J. Frisch, G. W. Trucks, H. B. Schlegel, G. E. Scuseria, M. A. Robb, J. R. Cheeseman, G. Scalmani, V. Barone, B. Mennucci, G. A. Petersson, H. Nakatsuji, M. Caricato, X. Li, H. P. Hratchian, A. F. Izmaylov, J. Bloino, G. Zheng, J. L. Sonnenberg, M. Hada, M. Ehara, K. Toyota, R. Fukuda, J. Hasegawa, M. Ishida, T. Nakajima, Y. Honda, O. Kitao, H. Nakai, T. Vreven, J. Montgomery, J. A., J. E. Peralta, F. Ogliaro, M. Bearpark, J. J. Heyd, E. Brothers, K. N. Kudin, V. N. Staroverov, R. Kobayashi, J. Normand, K. Raghavachari, A. Rendell, J. C. Burant, S. S. Iyengar, J. Tomasi, M. Cossi, N. Rega, J. M. Millam, M. Klene, J. E. Knox, J. B. Cross, V. Bakken, C. Adamo, J. Jaramillo, R. Gomperts, R. E. Stratmann, O. Yazyev, A. J. Austin, R. Cammi, C. Pomelli, J. W. Ochterski, R. L. Martin, K. Morokuma, V. G. Zakrzewski, G. A. Voth, P. Salvador, J. J. Dannenberg, S. Dapprich, A. D. Daniels, Ö. Farkas, J. B. Foresman, J. V. Ortiz, J. Cioslowski and D. J. Fox, Gaussian, Inc., Wallingford CT, **2016** .

XYZ Coordinates

All systems calculated at the PBE0-D3/def2-TZVP level of theory

TMA

Energy = -213.980103 hartree

Number of imaginary frequencies (PBE0-D3/def2-SVP) = 0

XYZ			
N	-0.471912	0.567059	0.000008
C	1.017374	0.567067	-0.000006
C	-0.968348	1.187193	1.259753
C	-0.968351	-0.833982	-0.092801
C	-0.968335	1.347941	-1.166938
H	-0.600767	-1.396533	0.763518
H	-0.600793	-1.278645	-1.015866
H	-2.056822	-0.823918	-0.092123
H	1.370693	-0.005635	0.855591
H	1.370688	1.594386	0.068141
H	1.370682	0.112450	-0.923778
H	-0.600748	2.369660	-1.090525
H	-2.056801	1.342382	-1.158561
H	-0.600770	0.887582	-2.082268
H	-0.600762	0.610137	2.106365
H	-2.056814	1.182720	1.250706
H	-0.600793	2.210076	1.318784

1

Energy = -2374.8697198 hartree

Number of imaginary frequencies (PBE0-D3/def2-SVP) = 0

XYZ			
N	0.029319	-3.981359	-2.195615
C	1.231359	-3.629899	-1.671931
C	1.262682	-2.961441	-0.463485
C	0.022696	-2.658469	0.113361
C	-1.191080	-2.979861	-0.438720
C	-1.203967	-3.663894	-1.684077

C	-2.527177	-2.572729	0.133639
C	-2.548734	-2.553796	1.659951
C	-3.900901	-2.214650	2.281390
C	-3.772321	-2.148619	3.795736
C	2.580658	-2.519423	0.133194
C	2.577332	-2.529423	1.659708
C	3.920453	-2.202031	2.307307
C	3.766900	-2.159619	3.820255
O	2.256786	-3.982213	-2.410583
O	-2.219264	-3.978868	-2.345402
C	-2.970260	-1.262497	-0.479797
C	-3.682223	-1.232461	-1.661956
N	-4.049203	-0.030401	-2.176334
C	-3.719836	1.203368	-1.671898
C	-2.990343	1.189815	-0.453486
C	-2.640856	-0.022894	0.080279
O	-4.059153	-2.263713	-2.380212
O	-4.059496	2.222045	-2.315400
C	-2.580651	2.519400	0.133689
C	-2.577234	2.529196	1.660203
C	-3.920318	2.201727	2.307840
C	-3.766678	2.159127	3.820774
C	-1.262710	2.961499	-0.463010
C	-1.231460	3.630118	-1.671367
N	-0.029451	3.981647	-2.195078
C	1.203866	3.664115	-1.683657
C	1.191053	2.979914	-0.438390
C	-0.022689	2.658448	0.113720
O	-2.256932	3.982532	-2.409909
O	2.219122	3.979179	-2.344999
C	2.527185	2.572708	0.133833
C	2.548836	2.553572	1.660141
C	3.901044	2.214354	2.281452
C	3.772558	2.148127	3.795798
C	2.970233	1.262558	-0.479805
C	3.682124	1.232680	-1.662011
N	4.049072	0.030688	-2.176574
C	3.719735	-1.203147	-1.672284

C	2.990315	-1.189758	-0.453826
C	2.640862	0.022879	0.080124
O	4.059011	2.264028	-2.380153
O	4.059353	-2.221739	-2.315943
C	-4.987375	-3.196656	1.869236
C	-5.012964	3.178766	1.899763
C	4.987487	3.196418	1.869357
C	5.013079	-3.179015	1.899047
H	-3.238792	-3.337388	-0.190686
H	-3.330620	3.245661	-0.192051
H	3.238779	3.337411	-0.190435
H	3.330609	-3.245639	-0.192687
H	0.027008	-2.127238	1.058376
H	2.073163	0.026884	1.005026
H	-0.026944	2.127090	1.058664
H	-2.073099	-0.027023	1.005146
H	-2.228857	-3.541026	2.012616
H	-1.806260	-1.847737	2.047619
H	-4.197549	-1.220605	1.925785
H	-4.710673	-4.220084	2.142500
H	-5.175082	-3.175002	0.793792
H	-5.929620	-2.962580	2.370170
H	-4.717582	-1.858704	4.260453
H	-3.013629	-1.422340	4.100492
H	-3.485006	-3.122089	4.205456
H	-1.831863	1.826579	2.048442
H	-2.250813	3.521497	1.992624
H	-4.222414	1.202643	1.972092
H	-5.945944	2.953939	2.421865
H	-5.221247	3.141209	0.828567
H	-4.730070	4.205849	2.152463
H	-3.472171	3.138677	4.210480
H	-3.003489	1.437109	4.124419
H	-4.704346	1.877383	4.305454
H	2.228975	3.540754	2.012957
H	1.806390	1.847458	2.047761
H	4.197674	1.220356	1.925701
H	4.710796	4.219809	2.142769

H	5.175125	3.174903	0.793898
H	5.929764	2.962282	2.370200
H	4.717850	1.858154	4.260419
H	3.013888	1.421806	4.100508
H	3.485267	3.121542	4.205662
H	1.831979	-1.826864	2.048085
H	2.250938	-3.521771	1.992015
H	4.222526	-1.202903	1.971666
H	4.730204	-4.206130	2.151637
H	5.946088	-2.954248	2.421121
H	5.221299	-3.141326	0.827843
H	3.003728	-1.437640	4.124034
H	4.704596	-1.877935	4.304916
H	3.472415	-3.139218	4.209856
H	3.376283	3.030071	-2.302625
H	3.046747	-3.334646	-2.302275
H	-4.538668	-0.037650	-3.061167
H	-0.036323	4.443114	-3.094845
H	4.538483	0.038058	-3.061436
H	0.036136	-4.442703	-3.095445
H	-3.376421	-3.029767	-2.302827
H	-3.046887	3.334950	-2.301638

1$_{OH}$

Energy = -2374.81247955 hartree

Number of imaginary frequencies (PBE0-D3/def2-SVP) = 1, no imaginary frequencies at the PBE0/def2-SVP level.

XYZ

scf done: -2374.812480

N	3.886775	-3.425337	-0.658682
C	4.136918	-2.129092	-0.625363
C	3.170321	-1.155338	-0.382673
C	1.893223	-1.633707	-0.145356
C	1.587727	-2.987018	-0.151139
C	2.650056	-3.838947	-0.432478
C	0.190733	-3.495709	0.122392
C	-0.096026	-3.533221	1.625838
C	0.768460	-4.496747	2.432098

C	0.436731	-5.949822	2.124587
C	3.535380	0.309610	-0.375864
C	4.032163	0.726917	1.011022
C	4.624707	2.130338	1.104704
C	4.969907	2.451216	2.551169
O	5.410379	-1.749552	-0.847207
C	-0.832621	-2.687739	-0.638307
C	-0.868974	-2.728336	-2.033279
N	-1.730913	-2.051717	-2.770507
C	-2.631479	-1.295768	-2.162385
C	-2.734271	-1.168489	-0.780584
C	-1.792685	-1.880797	-0.048922
O	0.012078	-3.506161	-2.683698
C	-3.814007	-0.324471	-0.158006
C	-4.260037	-0.832343	1.218728
C	-4.811350	-2.255081	1.253084
C	-5.137765	-2.645537	2.687074
C	-3.438867	1.136584	-0.071335
C	-4.432392	2.111405	-0.008610
N	-4.194023	3.406515	0.098307
C	-2.937612	3.813518	0.148200
C	-1.839856	2.960729	0.080892
C	-2.136401	1.610642	-0.029763
O	-5.726082	1.736016	-0.063163
C	-0.437476	3.518896	0.140359
C	-0.019260	3.741119	1.595892
C	1.249716	4.563888	1.806144
C	1.632672	4.555617	3.278477
C	0.535531	2.694812	-0.662511
C	0.486045	2.727392	-2.056206
N	1.301062	2.047538	-2.843328
C	2.236202	1.296311	-2.287072
C	2.421780	1.171135	-0.911912
C	1.526885	1.885514	-0.126529
O	-0.437025	3.498226	-2.655673
C	0.605520	-4.215446	3.918792
C	-6.030978	-2.415117	0.357186
C	1.092294	5.990463	1.299978

C	5.843996	2.295555	0.209247
H	0.142335	-4.517055	-0.265017
H	-4.680264	-0.374846	-0.822645
H	-0.489826	4.499607	-0.340769
H	4.373331	0.411357	-1.069176
H	1.091965	-0.920612	0.023175
H	1.620360	1.822872	0.950681
H	-1.319161	0.901577	-0.112177
H	-1.824384	-1.814009	1.030875
H	0.035098	-2.522213	2.029774
H	-1.148364	-3.800453	1.784617
H	1.817379	-4.318803	2.166896
H	-0.436023	-4.353529	4.227553
H	0.892851	-3.189704	4.164325
H	1.220058	-4.890575	4.519478
H	-0.606591	-6.165881	2.377779
H	1.066051	-6.623799	2.711335
H	0.591828	-6.189476	1.072065
H	-3.426377	-0.744071	1.924766
H	-5.033378	-0.150330	1.586882
H	-4.035309	-2.937179	0.886362
H	-6.814688	-1.705038	0.639748
H	-5.791486	-2.245986	-0.694604
H	-6.444919	-3.423004	0.442305
H	-4.259168	-2.561374	3.332703
H	-5.500492	-3.674858	2.745206
H	-5.915822	-1.994851	3.098911
H	-0.846628	4.249024	2.102980
H	0.079574	2.769709	2.094624
H	2.066174	4.098695	1.242387
H	0.258508	6.489005	1.804043
H	0.900513	6.027517	0.225932
H	1.997398	6.571805	1.492899
H	1.774768	3.536544	3.648295
H	2.560099	5.107148	3.451190
H	0.849097	5.021746	3.884370
H	3.217333	0.620499	1.736757
H	4.796198	0.004004	1.318005

H	3.866624	2.848295	0.770975
H	6.611807	1.557122	0.460964
H	6.281903	3.289341	0.331491
H	5.597592	2.172866	-0.847218
H	4.094769	2.361519	3.199920
H	5.356901	3.468478	2.650232
H	5.735557	1.765465	2.927828
H	-0.310944	3.388426	-3.605763
H	5.907226	-2.557786	-1.021667
H	-0.171634	-3.400961	-3.624934
H	-6.244672	2.548755	-0.030815
O	-3.493572	-0.628420	-2.950372
H	-3.268293	-0.861721	-3.858845
O	2.442970	-5.168995	-0.489448
H	3.292971	-5.564641	-0.715752
O	3.052207	0.627035	-3.120446
H	2.772854	0.853718	-4.015530
O	-2.733829	5.140779	0.266760
H	-3.607472	5.549613	0.266628

1_2

Energy = -4749.8156745 hartree

Number of imaginary frequencies (PBE0-D3/def2-SVP) = 0

XYZ

N	-0.943037	1.506230	-3.895703
C	-2.027075	2.064150	-3.309232
C	-1.895448	3.246626	-2.608095
C	-0.614796	3.804316	-2.573555
C	0.489582	3.243738	-3.170159
C	0.331045	1.997681	-3.831948
C	1.881609	3.825137	-3.072129
C	1.896606	5.351857	-3.064710
C	3.287942	5.979222	-3.044965
C	3.171174	7.493610	-2.960795
C	-3.084937	3.822308	-1.874335
C	-3.098339	5.349308	-1.868515
C	-4.329254	5.981269	-1.223876

C	-4.186245	7.495749	-1.212912
O	-3.138628	1.387697	-3.483217
O	1.261932	1.308778	-4.335859
C	2.610671	3.223615	-1.892125
C	3.346100	2.064809	-2.035746
N	3.943028	1.509685	-0.954792
C	3.874129	1.994010	0.322639
C	3.179550	3.220085	0.490171
C	2.557198	3.764511	-0.606186
O	3.541429	1.412876	-3.158932
O	4.400349	1.317070	1.249787
C	3.084938	3.822308	1.874334
C	3.098339	5.349309	1.868514
C	4.329256	5.981270	1.223878
C	4.186247	7.495751	1.212919
C	1.895448	3.246627	2.608094
C	2.027074	2.064151	3.309231
N	0.943036	1.506231	3.895702
C	-0.331045	1.997684	3.831948
C	-0.489581	3.243740	3.170159
C	0.614797	3.804318	2.573556
O	3.138626	1.387697	3.483217
O	-1.261935	1.308782	4.335859
C	-1.881608	3.825140	3.072128
C	-1.896605	5.351859	3.064709
C	-3.287941	5.979225	3.044965
C	-3.171173	7.493613	2.960796
C	-2.610668	3.223617	1.892124
C	-3.346095	2.064809	2.035745
N	-3.943025	1.509685	0.954791
C	-3.874128	1.994011	-0.322639
C	-3.179549	3.220086	-0.490172
C	-2.557196	3.764513	0.606184
O	-3.541419	1.412873	3.158930
O	-4.400348	1.317071	-1.249787
C	4.126670	5.562983	-4.244177
C	5.620516	5.561380	1.910364
C	-4.126668	5.562985	4.244177

C	-5.620516	5.561383	-1.910362
H	2.408649	3.502856	-3.974338
H	3.975411	3.496027	2.418537
H	-2.408649	3.502860	3.974336
H	-3.975411	3.496026	-2.418537
H	-0.486147	4.739409	-2.040175
H	-1.996951	4.683774	0.468950
H	0.486149	4.739411	2.040176
H	1.996952	4.683772	-0.468951
H	1.357027	5.699721	-3.953046
H	1.335324	5.738889	-2.207300
H	3.802572	5.631199	-2.141293
H	3.623227	5.824725	-5.180536
H	4.323186	4.488972	-4.256403
H	5.093550	6.071512	-4.233897
H	4.155488	7.962704	-2.892937
H	2.593295	7.801295	-2.084798
H	2.671335	7.895939	-3.847562
H	2.208326	5.740573	1.363708
H	3.022637	5.690210	2.907588
H	4.378304	5.640735	0.182920
H	6.477660	6.072116	1.465375
H	5.801456	4.487732	1.827344
H	5.596386	5.817739	2.974527
H	4.141320	7.890644	2.232794
H	3.274520	7.806757	0.695234
H	5.033194	7.968980	0.710652
H	-1.357025	5.699723	3.953043
H	-1.335324	5.738891	2.207298
H	-3.802572	5.631203	2.141293
H	-3.623222	5.824724	5.180536
H	-4.323186	4.488974	4.256402
H	-5.093546	6.071516	4.233900
H	-4.155486	7.962707	2.892936
H	-2.593291	7.801299	2.084800
H	-2.671335	7.895941	3.847565
H	-2.208325	5.740573	-1.363712
H	-3.022639	5.690211	-2.907590

H	-4.378302	5.640729	-0.182920
H	-5.596388	5.817747	-2.974524
H	-6.477659	6.072116	-1.465369
H	-5.801456	4.487733	-1.827348
H	-3.274519	7.806754	-0.695223
H	-5.033193	7.968977	-0.710647
H	-4.141315	7.890646	-2.232786
H	-2.702207	1.434225	3.724609
H	-3.726853	1.433117	-2.660700
H	4.354168	0.575936	-1.086652
H	1.071897	0.563081	4.288275
H	-4.354167	0.575938	1.086653
H	-1.071895	0.563079	-4.288274
H	2.702220	1.434228	-3.724615
H	3.726850	1.433114	2.660699
N	0.943037	-1.506230	-3.895703
C	2.027075	-2.064150	-3.309232
C	1.895448	-3.246626	-2.608095
C	0.614796	-3.804316	-2.573555
C	-0.489582	-3.243738	-3.170159
C	-0.331045	-1.997681	-3.831948
C	-1.881609	-3.825137	-3.072129
C	-1.896606	-5.351857	-3.064710
C	-3.287942	-5.979222	-3.044965
C	-3.171174	-7.493610	-2.960795
C	3.084937	-3.822308	-1.874335
C	3.098339	-5.349308	-1.868515
C	4.329254	-5.981269	-1.223876
C	4.186245	-7.495749	-1.212912
O	3.138628	-1.387697	-3.483217
O	-1.261932	-1.308778	-4.335859
C	-2.610671	-3.223615	-1.892125
C	-3.346100	-2.064809	-2.035746
N	-3.943028	-1.509685	-0.954792
C	-3.874129	-1.994010	0.322639
C	-3.179550	-3.220085	0.490171
C	-2.557198	-3.764511	-0.606186
O	-3.541429	-1.412876	-3.158932

O	-4.400349	-1.317070	1.249787
C	-3.084938	-3.822308	1.874334
C	-3.098339	-5.349309	1.868514
C	-4.329256	-5.981270	1.223878
C	-4.186247	-7.495751	1.212919
C	-1.895448	-3.246627	2.608094
C	-2.027074	-2.064151	3.309231
N	-0.943036	-1.506231	3.895702
C	0.331045	-1.997684	3.831948
C	0.489581	-3.243740	3.170159
C	-0.614797	-3.804318	2.573556
O	-3.138626	-1.387697	3.483217
O	1.261935	-1.308782	4.335859
C	1.881608	-3.825140	3.072128
C	1.896605	-5.351859	3.064709
C	3.287941	-5.979225	3.044965
C	3.171173	-7.493613	2.960796
C	2.610668	-3.223617	1.892124
C	3.346095	-2.064809	2.035745
N	3.943025	-1.509685	0.954791
C	3.874128	-1.994011	-0.322639
C	3.179549	-3.220086	-0.490172
C	2.557196	-3.764513	0.606184
O	3.541419	-1.412873	3.158930
O	4.400348	-1.317071	-1.249787
C	-4.126670	-5.562983	-4.244177
C	-5.620516	-5.561380	1.910364
C	4.126668	-5.562985	4.244177
C	5.620516	-5.561383	-1.910362
H	-2.408649	-3.502856	-3.974338
H	-3.975411	-3.496027	2.418537
H	2.408649	-3.502860	3.974336
H	3.975411	-3.496026	-2.418537
H	0.486147	-4.739409	-2.040175
H	1.996951	-4.683774	0.468950
H	-0.486149	-4.739411	2.040176
H	-1.996952	-4.683772	-0.468951
H	-1.357027	-5.699721	-3.953046

H	-1.335324	-5.738889	-2.207300
H	-3.802572	-5.631199	-2.141293
H	-3.623227	-5.824725	-5.180536
H	-4.323186	-4.488972	-4.256403
H	-5.093550	-6.071512	-4.233897
H	-4.155488	-7.962704	-2.892937
H	-2.593295	-7.801295	-2.084798
H	-2.671335	-7.895939	-3.847562
H	-2.208326	-5.740573	1.363708
H	-3.022637	-5.690210	2.907588
H	-4.378304	-5.640735	0.182920
H	-6.477660	-6.072116	1.465375
H	-5.801456	-4.487732	1.827344
H	-5.596386	-5.817739	2.974527
H	-4.141320	-7.890644	2.232794
H	-3.274520	-7.806757	0.695234
H	-5.033194	-7.968980	0.710652
H	1.357025	-5.699723	3.953043
H	1.335324	-5.738891	2.207298
H	3.802572	-5.631203	2.141293
H	3.623222	-5.824724	5.180536
H	4.323186	-4.488974	4.256402
H	5.093546	-6.071516	4.233900
H	4.155486	-7.962707	2.892936
H	2.593291	-7.801299	2.084800
H	2.671335	-7.895941	3.847565
H	2.208325	-5.740573	-1.363712
H	3.022639	-5.690211	-2.907590
H	4.378302	-5.640729	-0.182920
H	5.596388	-5.817747	-2.974524
H	6.477659	-6.072116	-1.465369
H	5.801456	-4.487733	-1.827348
H	3.274519	-7.806754	-0.695223
H	5.033193	-7.968977	-0.710647
H	4.141315	-7.890646	-2.232786
H	2.702207	-1.434225	3.724609
H	3.726853	-1.433117	-2.660700
H	-4.354168	-0.575936	-1.086652

H	-1.071897	-0.563081	4.288275
H	4.354167	-0.575938	1.086653
H	1.071895	-0.563079	-4.288274
H	-2.702220	-1.434228	-3.724615
H	-3.726850	-1.433114	2.660699

[**1**$_2$+Me$_4$N$_{endo}$]$^+$

Energy = -4963.853726 hartree

Number of imaginary frequencies (PBE0-D3/def2-SVP) = 0

XYZ

N	1.55696900	-0.94304600	3.91879000
C	2.12013500	-2.02760000	3.33223700
C	3.29691600	-1.88723400	2.62071400
C	3.85028900	-0.60123000	2.59385100
C	3.27615700	0.50602500	3.17400100
C	2.02611400	0.33995800	3.83053900
C	3.84744500	1.90502300	3.07178000
C	5.37410200	1.92953200	3.05819600
C	5.99316100	3.32506900	3.04363900
C	7.50782600	3.21396100	2.95664400
C	3.88648800	-3.07284300	1.88442800
C	5.41371000	-3.06483000	1.87420300
C	6.06145600	-4.29496200	1.24279000
C	7.57343300	-4.12747700	1.22861100
O	1.44173300	-3.13610900	3.50613300
O	1.30673400	1.26538000	4.29328400
C	3.23784400	2.63486900	1.89266200
C	2.07376200	3.36531000	2.03581900
N	1.51915700	3.96038400	0.95026900
C	2.00019100	3.88725600	-0.33065500
C	3.23243200	3.19969100	-0.49742300
C	3.78703900	2.59791500	0.60618300
O	1.40587800	3.54895700	3.14845000
O	1.30510600	4.38871200	-1.25407200
C	3.83455700	3.09999300	-1.88445000
C	5.36147700	3.11624800	-1.87822800
C	5.99064900	4.35710800	-1.24906900

C	7.50521900	4.21481700	-1.23948100
C	3.26062500	1.90482400	-2.61664500
C	2.06384200	2.02320700	-3.29905600
N	1.50733200	0.92769400	-3.87168300
C	2.01030900	-0.34474400	-3.81059700
C	3.27177200	-0.48968600	-3.17201100
C	3.83883700	0.62985200	-2.60478700
O	1.35585000	3.11453800	-3.45187800
O	1.30539500	-1.28021700	-4.27600000
C	3.86551000	-1.87978800	-3.07471300
C	5.39243600	-1.88035400	-3.06794900
C	6.03342100	-3.26600200	-3.05944900
C	7.54656300	-3.13108500	-2.97983800
C	3.27339200	-2.62257400	-1.89403100
C	2.12625000	-3.37900200	-2.03310900
N	1.58867300	-3.98782500	-0.94677200
C	2.06026000	-3.88757000	0.33639200
C	3.28466800	-3.18443800	0.49780500
C	3.82567500	-2.57509100	-0.60976900
O	1.45154500	-3.57213200	-3.14120400
O	1.36390300	-4.37693900	1.26496900
C	5.57492300	4.15472300	4.24852000
C	5.56789300	5.63823300	-1.95267200
C	5.62217400	-4.09974800	-4.26390200
C	5.66237100	-5.58300100	1.94757800
H	3.52626900	2.43062200	3.97520700
H	3.50742300	3.98872600	-2.43111800
H	3.54887200	-2.40847700	-3.97797000
H	3.57526400	-3.96706000	2.43127800
H	4.79057400	-0.47034100	2.07016600
H	4.74530200	-2.01364700	-0.48028200
H	4.79063400	0.51542500	-2.09848300
H	4.71642800	2.05357200	0.47390300
H	5.72692200	1.38911600	3.94353600
H	5.76212200	1.37279900	2.19788500
H	5.64309600	3.84369100	2.14246900
H	5.84191000	3.64856600	5.18150600
H	4.50038600	4.35000200	4.26626800

H	6.07945700	5.12304500	4.24222500
H	7.97167100	4.20034600	2.89242700
H	7.81795600	2.64144200	2.07800200
H	7.91378100	2.71364300	3.84094500
H	5.75523100	2.23261700	-1.36359500
H	5.70177900	3.03003500	-2.91628100
H	5.65320100	4.41947200	-0.20738600
H	6.07922400	6.50066900	-1.52025900
H	4.49462800	5.82394600	-1.87041800
H	5.82350900	5.60109100	-3.01627700
H	7.89867200	4.15981500	-2.25896000
H	7.81944900	3.31007400	-0.71160500
H	7.97734900	5.06805800	-0.74827300
H	5.73322600	-1.33239900	-3.95342400
H	5.77539800	-1.32009300	-2.20765200
H	5.69612800	-3.79174700	-2.15754800
H	5.87545600	-3.58721400	-5.19723300
H	4.55095800	-4.31292100	-4.27604400
H	6.14272100	-5.05956700	-4.26264600
H	8.02620400	-4.11015000	-2.92002500
H	7.85202000	-2.55559700	-2.10150100
H	7.94014900	-2.62263500	-3.86509800
H	5.79177500	-2.17451800	1.35934900
H	5.75488400	-2.97352800	2.91148300
H	5.72207000	-4.36301000	0.20205900
H	5.92116100	-5.54179800	3.01025300
H	6.18613400	-6.43692000	1.51313600
H	4.59198600	-5.78625500	1.86930200
H	7.87092100	-3.21776500	0.69955700
H	8.05830100	-4.97291700	0.73630400
H	7.96891100	-4.06547500	2.24689600
H	1.47707800	-2.74035100	-3.71492200
H	1.50494500	-3.73559600	2.69453400
H	0.59764800	4.39357200	1.08278300
H	0.56999500	1.05090400	-4.27823500
H	0.67093500	-4.42770200	-1.07883600
H	0.63160700	-1.07637600	4.34243400
H	1.44807500	2.71658000	3.72294000

H	1.43266200	3.72786500	-2.64569400
N	-1.59407200	0.98059100	3.98547100
C	-2.12978300	2.05203300	3.35098700
C	-3.28786800	1.90148100	2.61303100
C	-3.84130900	0.61634600	2.58937000
C	-3.28914700	-0.48044100	3.20902400
C	-2.05974500	-0.30568900	3.90137000
C	-3.85537000	-1.87926400	3.09464000
C	-5.38227000	-1.90681600	3.07967400
C	-5.99855300	-3.30342200	3.05691800
C	-7.51335600	-3.19494100	2.96913200
C	-3.87197200	3.07937700	1.86409200
C	-5.40004900	3.07710700	1.86111200
C	-6.04631400	4.30362900	1.22134500
C	-7.55901800	4.14221500	1.21734500
O	-1.44517200	3.16068000	3.50668000
O	-1.34794800	-1.22685000	4.38299300
C	-3.24121700	-2.59649100	1.91077000
C	-2.07326200	-3.31800400	2.05234600
N	-1.51377000	-3.91113200	0.96846300
C	-2.00057200	-3.84965200	-0.31135700
C	-3.23922800	-3.17390200	-0.47567500
C	-3.78987600	-2.56063800	0.62449100
O	-1.39927500	-3.49185300	3.16463200
O	-1.30419500	-4.34226600	-1.23869500
C	-3.85280300	-3.10782900	-1.85892400
C	-5.37996400	-3.12890000	-1.84215300
C	-6.00098800	-4.35954300	-1.18551700
C	-7.51577400	-4.22065800	-1.16572500
C	-3.29036700	-1.92845100	-2.62239800
C	-2.13397100	-2.06434200	-3.36422600
N	-1.58916900	-0.97642600	-3.96306500
C	-2.04928700	0.30843700	-3.84475800
C	-3.28823900	0.46722700	-3.16404500
C	-3.84311900	-0.64216800	-2.57135900
O	-1.44240700	-3.16785700	-3.53540300
O	-1.33909800	1.24167000	-4.30427000
C	-3.88332400	1.85663200	-3.07809600

C	-5.41082900	1.85135300	-3.06430100
C	-6.05585900	3.23503200	-3.06343400
C	-7.56834500	3.09642800	-2.97801800
C	-3.29065500	2.61603900	-1.91216700
C	-2.15830400	3.38841300	-2.06916500
N	-1.61278500	4.00596400	-0.99084900
C	-2.06340700	3.89434900	0.29953800
C	-3.27835400	3.17859000	0.47584100
C	-3.82431300	2.55731700	-0.62009400
O	-1.50060500	3.57884400	-3.18993700
O	-1.35847300	4.37865000	1.22362600
C	-5.58002600	-4.13881200	4.25772800
C	-5.58145900	-5.65217300	-1.86979500
C	-5.65081400	4.06180900	-4.27479600
C	-5.63805100	5.59694600	1.91107000
H	-3.53534400	-2.41127800	3.99452500
H	-3.52726800	-4.00796500	-2.38681500
H	-3.57410900	2.37412300	-3.99020500
H	-3.55688200	3.97813800	2.40106400
H	-4.76294100	0.47621000	2.03588400
H	-4.73381300	1.98294300	-0.47829900
H	-4.76984700	-0.51638500	-2.02311000
H	-4.72375200	-2.02423300	0.49196100
H	-5.73631400	-1.37235400	3.96812500
H	-5.77082100	-1.34537000	2.22273900
H	-5.64639400	-3.81649600	2.15349900
H	-5.84801400	-3.63781900	5.19321300
H	-4.50528300	-4.33293100	4.27498400
H	-6.08341300	-5.10770500	4.24613100
H	-7.97515900	-4.18182400	2.89785000
H	-7.82379700	-2.61719200	2.09407400
H	-7.92115600	-2.70129700	3.85633700
H	-5.77334800	-2.23638300	-1.34291700
H	-5.72655900	-3.06396800	-2.87965400
H	-5.65456400	-4.40279000	-0.14587200
H	-6.08708800	-6.50789100	-1.41771700
H	-4.50711800	-5.83414000	-1.79360700
H	-5.84625500	-5.63444000	-2.93165300

H	-7.91776100	-4.18522000	-2.18274700
H	-7.82777600	-3.30708800	-0.65200700
H	-7.98178800	-5.06580600	-0.65500300
H	-5.75360700	1.29497200	-3.94369800
H	-5.78762100	1.29664500	-2.19785800
H	-5.71708100	3.76764200	-2.16609400
H	-5.90528500	3.54207700	-5.20382900
H	-4.58027500	4.27812100	-4.29160100
H	-6.17435500	5.02001200	-4.27860900
H	-8.05055500	4.07452800	-2.92286400
H	-7.86924600	2.52565500	-2.09506200
H	-7.96336100	2.58120600	-3.85871700
H	-5.78388300	2.18333200	1.35683500
H	-5.73627700	2.99701300	2.90093500
H	-5.71266900	4.35988800	0.17808700
H	-5.89032200	5.56706600	2.97570100
H	-6.16130500	6.44861500	1.47159800
H	-4.56739500	5.79537300	1.82426700
H	-7.86305900	3.22864200	0.69881000
H	-8.04327100	4.98481300	0.71955500
H	-7.94898700	4.09165700	2.23839100
H	-1.51761700	2.74029300	-3.74762000
H	-1.50161000	3.74128500	2.68463200
H	-0.59190200	-4.34173900	1.10427900
H	-0.66405700	-1.10804800	-4.38544400
H	-0.69867700	4.44835800	-1.12922300
H	-0.66925500	1.11497500	4.40737800
H	-1.46951000	-2.67657600	3.75706900
H	-1.47685600	-3.74491700	-2.70837700
C	-0.27945900	-1.11071300	-0.99459400
H	0.18567000	-0.93595100	-1.96080900
H	0.05050900	-2.06427500	-0.59578300
H	-1.36354000	-1.10235800	-1.08614000
C	-0.46684800	-0.29783200	1.26765300
H	-0.12211900	-1.26477500	1.62679300
H	-0.14352200	0.48804400	1.94491500
H	-1.55126400	-0.29675900	1.18111500
C	-0.35783600	1.26396900	-0.56136000

H	-0.05407900	2.03322900	0.14297900
H	0.07919800	1.45622300	-1.53832300
H	-1.44259700	1.22855700	-0.63831600
C	1.59969500	-0.01123900	0.06012300
H	1.94004300	-0.97160600	0.43898500
H	2.03213900	0.17688300	-0.91889500
H	1.87909400	0.78000300	0.75102800
N	0.12660000	-0.04038000	-0.06131700

[**1**$_2$+Me$_4$N$_{exo}$]$^+$

Energy = -4963.810052 hartree

Number of imaginary frequencies (PBE0-D3/def2-SVP) = 0

XYZ

N	-0.91307100	0.97605900	3.96283600
C	-1.47598400	2.06220800	3.38562700
C	-2.67737100	1.93778200	2.71217400
C	-3.24312100	0.66169100	2.69386500
C	-2.68381400	-0.44319500	3.28635300
C	-1.42109900	-0.29327700	3.92147700
C	-3.27926100	-1.83002000	3.20745200
C	-4.80571600	-1.82139200	3.23796200
C	-5.46796000	-3.19584600	3.20667600
C	-6.98154800	-3.03696800	3.22163800
C	-3.26854800	3.12514300	1.98708100
C	-4.79518800	3.13466500	2.02636300
C	-5.46259500	4.33776300	1.36597100
C	-6.97536500	4.20363200	1.46630300
O	-0.78983600	3.16797800	3.53843500
O	-0.73948900	-1.23038600	4.41476400
C	-2.71429600	-2.57143500	2.01770600
C	-1.55284400	-3.31288100	2.13315600
N	-1.03080800	-3.91780600	1.04053100
C	-1.55380100	-3.85590300	-0.22240200
C	-2.77892300	-3.14929100	-0.36035600
C	-3.28534500	-2.51614200	0.74689200
O	-0.87519700	-3.50325600	3.23738500
O	-0.91801900	-4.39347300	-1.16713700

C	-3.40892500	-3.05685700	-1.73293500
C	-4.93406400	-3.07752300	-1.67939800
C	-5.51386400	-4.37684200	-1.12347900
C	-6.96406700	-4.18104100	-0.71148100
C	-2.85104000	-1.87406700	-2.48770700
C	-1.68187100	-2.01590700	-3.21399500
N	-1.13118100	-0.93977800	-3.81968600
C	-1.61876400	0.33594100	-3.75566400
C	-2.84890200	0.50518000	-3.06147200
C	-3.40070500	-0.59056300	-2.44378400
O	-1.01925300	-3.13098800	-3.39323200
O	-0.94980200	1.26284100	-4.28220900
C	-3.40106400	1.90605800	-2.95434500
C	-4.92554500	1.96159900	-2.90759000
C	-5.49241700	3.37687800	-2.99876900
C	-6.94437800	3.40077000	-2.54891300
C	-2.76421500	2.63322000	-1.79060800
C	-1.60221800	3.36102000	-1.96387700
N	-1.01825800	3.95899400	-0.89957200
C	-1.47831300	3.90496300	0.38862600
C	-2.70513300	3.21381600	0.58733700
C	-3.27236100	2.58564000	-0.49114200
O	-0.97876900	3.54629900	-3.10183800
O	-0.78962500	4.43341900	1.29901400
C	-5.00746800	-4.08688100	4.35014800
C	-5.38211000	-5.52423800	-2.11377100
C	-5.34826800	3.95556600	-4.39832900
C	-4.99747800	5.65771700	1.96166700
H	-2.94514900	-2.35664500	4.10564500
H	-3.09871300	-3.95257000	-2.27850500
H	-3.09148500	2.42402800	-3.86639100
H	-2.93068100	4.01929400	2.51810500
H	-4.18769400	0.53755800	2.17621900
H	-4.18548300	2.02285000	-0.32330800
H	-4.31824300	-0.45165100	-1.87973600
H	-4.19877400	-1.94010000	0.63533500
H	-5.12018800	-1.28946100	4.14312400
H	-5.19870600	-1.23364500	2.39877200

H	-5.18769400	-3.68714900	2.26693900
H	-5.19440500	-3.60937500	5.31697600
H	-3.94144600	-4.31197400	4.28662500
H	-5.54299200	-5.03868300	4.34024100
H	-7.48588900	-4.00109200	3.12608000
H	-7.32151800	-2.39831500	2.39806100
H	-7.31749000	-2.57643300	4.15563300
H	-5.29356100	-2.23754400	-1.07062600
H	-5.32126800	-2.90828700	-2.69146700
H	-4.94681300	-4.63606300	-0.22222900
H	-5.77352700	-6.45157500	-1.69054600
H	-4.34247300	-5.70722400	-2.39010100
H	-5.94041700	-5.31279700	-3.03118700
H	-7.57396500	-3.85588100	-1.56312800
H	-7.03684300	-3.43519900	0.08803200
H	-7.40402500	-5.10563900	-0.33188800
H	-5.32267200	1.35385200	-3.72953800
H	-5.28387400	1.49403500	-1.98106500
H	-4.92243800	4.00901000	-2.30820400
H	-5.90510600	3.35734100	-5.12642900
H	-4.30621900	3.98903600	-4.72021900
H	-5.73364400	4.97636400	-4.43986000
H	-7.37419900	4.40165500	-2.62638600
H	-7.02429500	3.09430200	-1.49983300
H	-7.55856300	2.73378800	-3.16599000
H	-5.19204900	2.22465000	1.55892300
H	-5.10276500	3.08417600	3.07723000
H	-5.19144900	4.33651200	0.30348100
H	-5.17651000	5.68375600	3.04103500
H	-5.53617300	6.49619800	1.51493200
H	-3.93255700	5.82769900	1.79361200
H	-7.31897700	3.25589300	1.03560300
H	-7.48446700	5.01359900	0.93922100
H	-7.30219900	4.22834400	2.51026000
H	-1.02373600	2.71059300	-3.66858800
H	-0.85788400	3.76046200	2.72310400
H	-0.08737900	-4.32512100	1.14671800
H	-0.18854500	-1.07440000	-4.22369500

H	-0.07570500	4.35278000	-1.05296700
H	0.04783300	1.09684500	4.32377900
H	-0.87969400	-2.66475900	3.80691600
H	-1.04777000	-3.71959300	-2.56891300
N	2.08256000	-0.93318100	3.90025700
C	2.61481100	-2.02452600	3.30346000
C	3.77529700	-1.90370200	2.56665800
C	4.34039400	-0.62657100	2.51233900
C	3.80275800	0.48600800	3.11607600
C	2.57343600	0.33898100	3.80922100
C	4.39608800	1.87177200	2.99729500
C	5.92216800	1.87085900	2.94776000
C	6.56298600	3.25589900	2.91173300
C	8.07280000	3.12232700	2.78256400
C	4.32254000	-3.10046300	1.82302900
C	5.84881500	-3.12408500	1.77491600
C	6.45541300	-4.36318000	1.12136700
C	7.96976200	-4.22835700	1.06864800
O	1.93273700	-3.12934500	3.50313200
O	1.89364600	1.27569200	4.31815400
C	3.76972800	2.60180000	1.83089900
C	2.62409100	3.35000900	2.00309700
N	2.04680100	3.94719400	0.93371400
C	2.48828400	3.86073900	-0.35756400
C	3.70456900	3.15932500	-0.55508300
C	4.27505200	2.54037600	0.53080700
O	2.00084400	3.55704900	3.14092500
O	1.77859000	4.37889400	-1.26709500
C	4.27351400	3.05479600	-1.95290600
C	5.80043200	3.05459000	-1.98341700
C	6.45873900	4.28274400	-1.35982100
C	7.97164100	4.12632300	-1.38850800
C	3.66968900	1.86737900	-2.66713400
C	2.47438100	2.00752200	-3.34222500
N	1.89416600	0.92486900	-3.90968500
C	2.36777400	-0.35529400	-3.84313700
C	3.62920900	-0.52251800	-3.21594300
C	4.21603200	0.58136500	-2.64235600

O	1.80085700	3.12317600	-3.50621200
O	1.64567900	-1.28041700	-4.31460500
C	4.20647100	-1.91737900	-3.12786200
C	5.73292700	-1.93996500	-3.15846700
C	6.35360800	-3.33458100	-3.15311100
C	7.87013100	-3.22384400	-3.10897000
C	3.63093200	-2.63852100	-1.93027000
C	2.46715600	-3.36926000	-2.04267300
N	1.93702200	-3.95820300	-0.94489200
C	2.44511600	-3.87837600	0.32211700
C	3.68099700	-3.19586400	0.45630100
C	4.20400600	-2.58556100	-0.65787800
O	1.78262300	-3.56661100	-3.14708600
O	1.77468100	-4.38499500	1.26677100
C	6.19200000	4.09618700	4.12469700
C	6.03188600	5.57486400	-2.04042800
C	5.90247100	-4.17256200	-4.34021100
C	6.04754600	-5.64708800	1.82866000
H	4.10517400	2.40621400	3.90577400
H	3.94341400	3.94635100	-2.49301500
H	3.86027800	-2.44688100	-4.01969800
H	4.00667000	-3.98681400	2.37993400
H	5.26321600	-0.50748000	1.95622200
H	5.13226500	-2.03489800	-0.54672200
H	5.16483600	0.44711700	-2.13562300
H	5.18762500	1.97548800	0.37163100
H	6.28845100	1.32745200	3.82607500
H	6.27977900	1.30598900	2.07999400
H	6.19442900	3.77645200	2.01942700
H	6.47617300	3.58793700	5.05168900
H	5.12136900	4.30668800	4.17029100
H	6.71154000	5.05667600	4.10127400
H	8.55017700	4.10142200	2.70242700
H	8.34856900	2.54284600	1.89709300
H	8.49582700	2.61711000	3.65630300
H	6.19622100	2.16285000	-1.48519700
H	6.11493000	2.97222500	-3.03011100
H	6.14676400	4.33875900	-0.31012900

H	6.56194800	6.42872100	-1.61269400
H	4.96251100	5.76721900	-1.92981700
H	6.26032400	5.54490100	-3.11064100
H	8.33929600	4.07570400	-2.41803900
H	8.28886800	3.21359000	-0.87650500
H	8.46487100	4.97041100	-0.90149700
H	6.06086300	-1.40338100	-4.05594500
H	6.14432400	-1.37959900	-2.31188900
H	6.02778900	-3.84686200	-2.23964000
H	6.14256500	-3.67180400	-5.28362000
H	4.82769500	-4.36578400	-4.32513200
H	6.40720000	-5.14119200	-4.34193800
H	8.33629200	-4.20993100	-3.05183900
H	8.20375400	-2.64610500	-2.24248800
H	8.25131400	-2.72777600	-4.00687500
H	6.23201800	-2.23928700	1.25499800
H	6.21791000	-3.04483800	2.80386000
H	6.08700100	-4.41765800	0.09003200
H	6.33401500	-5.61728500	2.88481300
H	6.54138600	-6.50978700	1.37602300
H	4.97111100	-5.82426400	1.77691700
H	8.27208900	-3.32201300	0.53665700
H	8.42433700	-5.08113100	0.55963500
H	8.39249300	-4.17948400	2.07689200
H	1.79612400	-2.73079700	-3.71580200
H	1.95246800	-3.72050500	2.68331600
H	1.13291100	4.38391300	1.09235900
H	0.95429100	1.06356600	-4.29664300
H	1.00928900	-4.38006900	-1.05572800
H	1.16080500	-1.05698000	4.33418000
H	2.03233100	2.72140800	3.71000900
H	1.87215900	3.71334700	-2.68746100
C	-7.28781800	0.09423100	0.35978800
H	-7.57253400	0.33794700	1.38170000
H	-6.70917500	-0.82777300	0.34462300
H	-6.70764700	0.90935500	-0.06936900
C	-8.12216300	-0.43411700	-1.85490200
H	-7.53269100	-1.34944600	-1.84316000

H	-9.02296000	-0.57570700	-2.44940200
H	-7.53146900	0.38665100	-2.25837400
C	-9.32198300	1.15145100	-0.45105100
H	-10.22330700	0.99034800	-1.03973900
H	-9.58606100	1.39488100	0.57655500
H	-8.73148800	1.95481900	-0.88643600
C	-9.32426500	-1.21141900	0.11346900
H	-8.73575800	-2.12611100	0.08746600
H	-9.58745200	-0.96407700	1.14036300
H	-10.22604900	-1.33151700	-0.48426100
N	-8.51596500	-0.09976800	-0.45763200

[$\mathbf{1}_2$+Me_4N_{exo2}]$^+$

Energy = -4963.8331111 hartree

Number of imaginary frequencies (PBE0-D3/def2-SVP) = 0

XYZ			
N	-1.36744500	-2.80966300	-2.30329600
C	-1.93212900	-3.34052800	-1.19174200
C	-3.15553000	-2.87947300	-0.76131400
C	-3.74937500	-1.88195200	-1.54455200
C	-3.18562000	-1.34366400	-2.67723300
C	-1.89778500	-1.80162400	-3.06354600
C	-3.80848300	-0.21666800	-3.47003600
C	-5.33404100	-0.27310700	-3.50578400
C	-5.99244500	0.80884000	-4.35788600
C	-7.50591100	0.69581000	-4.25598800
C	-3.72952100	-3.39562400	0.53873500
C	-5.25477600	-3.46355900	0.53264200
C	-5.87929600	-4.06965800	1.78688100
C	-7.39603300	-4.01027700	1.68759600
O	-1.21595500	-4.28614300	-0.62038400
O	-1.20203300	-1.35541200	-4.01673700
C	-3.28010400	1.10945200	-2.97134600
C	-2.15437200	1.67565500	-3.52666800
N	-1.66644300	2.83431600	-3.02673800
C	-2.19301200	3.52311900	-1.96802900
C	-3.39878100	3.00444100	-1.42249100

C	-3.87116900	1.81646000	-1.91854600
O	-1.46685000	1.19397800	-4.54332600
O	-1.57100000	4.53255700	-1.54193600
C	-4.05855000	3.74290900	-0.27875100
C	-5.58349100	3.66501100	-0.31402000
C	-6.22925200	4.21279300	-1.58431100
C	-7.73549800	4.00762900	-1.52553100
C	-3.47551900	3.28553100	1.03883000
C	-2.33402000	3.88546100	1.53438700
N	-1.77052500	3.42083600	2.67308000
C	-2.21569500	2.34937800	3.39443400
C	-3.42443800	1.75404600	2.95067200
C	-3.99134500	2.22760100	1.79083300
O	-1.70211300	4.90581200	1.00362300
O	-1.51676800	1.95620500	4.37165500
C	-3.96059500	0.56858300	3.72050900
C	-5.48453300	0.47895900	3.70689600
C	-6.06834900	-0.65659600	4.54365100
C	-7.58080700	-0.69006400	4.38368000
C	-3.29010400	-0.69705300	3.23668700
C	-2.10095700	-1.12173300	3.80102500
N	-1.48449800	-2.22264000	3.30912800
C	-1.92523400	-2.96871700	2.25542700
C	-3.16902800	-2.59853800	1.69700700
C	-3.78542100	-1.47025200	2.19075900
O	-1.47697500	-0.55088500	4.79901300
O	-1.17732300	-3.91259300	1.84278200
C	-5.53770400	0.75882500	-5.80895000
C	-5.88827500	5.67668100	-1.82050000
C	-5.67805600	-0.55287900	6.01062600
C	-5.40527600	-5.49354000	2.03758600
H	-3.45901300	-0.33757400	-4.49893300
H	-3.78978100	4.79666900	-0.39186600
H	-3.65653700	0.71750100	4.76011200
H	-3.36273300	-4.41923600	0.65671700
H	-4.71907900	-1.50990400	-1.23466400
H	-4.72589200	-1.16704200	1.74314700
H	-4.89829100	1.75167200	1.43550300

H	-4.77333600	1.40483600	-1.47793100
H	-5.62961500	-1.25862200	-3.88395000
H	-5.74663100	-0.20762500	-2.49326800
H	-5.70019600	1.78380400	-3.94979700
H	-5.74256100	-0.22324600	-6.24764900
H	-4.46969800	0.96100700	-5.91512900
H	-6.06952500	1.50381600	-6.40463900
H	-7.99915700	1.49334200	-4.81569500
H	-7.84261800	0.75985600	-3.21760900
H	-7.85454800	-0.25900600	-4.66169800
H	-5.92041200	2.63163700	-0.17586300
H	-5.96733400	4.21876100	0.55031100
H	-5.84487200	3.63802700	-2.43550400
H	-6.40994400	6.05648000	-2.70170400
H	-4.81965000	5.83231000	-1.98324900
H	-6.19161100	6.28964800	-0.96582500
H	-8.17381000	4.57099800	-0.69608400
H	-7.99046000	2.95363700	-1.38315800
H	-8.21608000	4.34600000	-2.44611400
H	-5.88059700	1.43370400	4.07030300
H	-5.85674400	0.37656600	2.68155100
H	-5.66887900	-1.60279800	4.15792100
H	-5.99530600	0.40692800	6.43037700
H	-4.60025700	-0.64552300	6.16015500
H	-6.15640200	-1.34305000	6.59350600
H	-8.01758200	-1.52668700	4.93345700
H	-7.87010000	-0.79199000	3.33390500
H	-8.03354800	0.23007700	4.76541000
H	-5.68430200	-2.46733600	0.38227200
H	-5.56260400	-4.05165100	-0.33989400
H	-5.57465700	-3.46046500	2.64585200
H	-5.62717400	-6.13416600	1.17764700
H	-5.91110000	-5.92038800	2.90627100
H	-4.33127000	-5.54400400	2.23011700
H	-7.74668200	-2.98577800	1.53631000
H	-7.86594700	-4.39197200	2.59649400
H	-7.75682600	-4.61335700	0.84857000
H	-1.56666300	0.46343600	4.74346000

H	-1.26118700	-4.21152400	0.41682900
H	-0.74934800	3.13582900	-3.37696000
H	-0.85518500	3.81864900	2.92372500
H	-0.54439800	-2.41971800	3.67661800
H	-0.39240300	-3.06774700	-2.48837600
H	-1.42336100	0.19719500	-4.48114000
H	-1.73592400	4.87201600	-0.00401400
N	1.57809800	-1.19566400	-3.42368500
C	2.06332400	0.05559800	-3.60372800
C	3.23689900	0.42983000	-2.96352600
C	3.83638200	-0.53462600	-2.16378400
C	3.33628300	-1.80980100	-1.97232800
C	2.13274800	-2.14549900	-2.62013900
C	3.96513800	-2.80590600	-1.02305700
C	5.49003500	-2.74614100	-0.99697100
C	6.15084100	-3.81517800	-0.13019900
C	7.66109500	-3.63648800	-0.14800300
C	3.74432400	1.84641100	-3.10754900
C	5.26831900	1.93760400	-3.07894900
C	5.83327200	3.33533100	-3.31906600
C	7.34888600	3.30593300	-3.19219600
O	1.34669700	0.80250300	-4.39375500
O	1.50430800	-3.25891300	-2.49800900
C	3.34480700	-2.68125300	0.35066300
C	2.21722100	-3.39753600	0.66781600
N	1.62218600	-3.24003300	1.87007600
C	2.04345800	-2.37575000	2.84658200
C	3.24755400	-1.66320500	2.57529500
C	3.82951400	-1.82035400	1.34511900
O	1.62117200	-4.27001700	-0.13413000
O	1.34008800	-2.25530600	3.88025000
C	3.77589900	-0.71042700	3.62499100
C	5.30106900	-0.63622400	3.65660400
C	6.00891400	-1.96336600	3.91859800
C	7.51558300	-1.76781100	3.83917000
C	3.12304000	0.64432100	3.46576700
C	1.92701700	0.91762100	4.09767700
N	1.30853400	2.10185500	3.88705700

C	1.75419200	3.08797300	3.05162200
C	3.02026500	2.86921000	2.44429400
C	3.63750000	1.66057900	2.65343600
O	1.28696000	0.12228800	4.92677100
O	1.01368000	4.09059300	2.86151800
C	3.55992300	3.93939600	1.52342100
C	5.08418200	4.02935600	1.53147900
C	5.66467700	5.14995600	0.67264900
C	7.18433600	5.08678300	0.70058300
C	2.98754200	3.75610600	0.13645300
C	1.79730300	4.36014000	-0.22291900
N	1.27552200	4.13561400	-1.45190700
C	1.82230700	3.32221800	-2.40319700
C	3.07194700	2.73762200	-2.08740400
C	3.58796500	2.95415900	-0.83156500
O	1.08608800	5.16223600	0.53036800
O	1.17090800	3.12856300	-3.47267600
C	5.76368000	-5.22464000	-0.55641800
C	5.60955800	-2.57690300	5.25250600
C	5.16811100	6.52277900	1.10133400
C	5.40962400	3.90656500	-4.66409000
H	3.69854700	-3.79701600	-1.39873900
H	3.45397500	-1.10323100	4.59310400
H	3.18038200	4.89021700	1.90694300
H	3.42016700	2.19519300	-4.09201100
H	4.75474100	-0.26771000	-1.65386200
H	4.53233000	2.48298600	-0.57951300
H	4.58686800	1.48231700	2.16106900
H	4.72887800	-1.25255700	1.13068700
H	5.85350800	-2.84111400	-2.02680300
H	5.83311700	-1.76555700	-0.65198500
H	5.80672300	-3.67506500	0.90140300
H	6.00391700	-5.39036100	-1.61266000
H	4.69763300	-5.41664000	-0.40881500
H	6.31108700	-5.96824100	0.02671400
H	8.15217100	-4.35623100	0.51029700
H	7.94659500	-2.63452900	0.18259600
H	8.06080800	-3.78049300	-1.15655100

H	5.68646600	-0.21907000	2.71977000
H	5.58422600	0.08026900	4.43571300
H	5.71908800	-2.66550500	3.12709300
H	6.17290200	-3.49379400	5.43957600
H	4.54856900	-2.83300000	5.29014900
H	5.81804000	-1.88643500	6.07571600
H	7.86070700	-1.07846500	4.61562400
H	7.81514200	-1.35451600	2.87182500
H	8.04468100	-2.71331200	3.97767000
H	5.40804000	4.16670300	2.56924300
H	5.52912700	3.08238600	1.20602400
H	5.34076600	4.98610700	-0.36248700
H	5.40487200	6.71129400	2.15327600
H	4.08888300	6.63082600	0.97443000
H	5.64491500	7.30696100	0.50926500
H	7.62289100	5.84755900	0.05115600
H	7.55045800	4.11148700	0.36738700
H	7.56319400	5.25651600	1.71306800
H	5.65943600	1.56978400	-2.12390000
H	5.66189000	1.25709200	-3.84306400
H	5.44330400	3.99692300	-2.53677900
H	5.71560700	3.24622800	-5.48199200
H	5.87646400	4.87929800	-4.83269300
H	4.32908000	4.04964700	-4.73198100
H	7.66141400	2.92285100	-2.21673600
H	7.77325500	4.30537000	-3.30860600
H	7.79292800	2.66548900	-3.96064700
H	1.11925800	4.85914400	1.49612700
H	1.35442800	1.79445000	-4.10379700
H	0.70192500	-3.68274700	1.99649700
H	0.35763500	2.19480500	4.27008700
H	0.32521200	4.49420000	-1.61893900
H	0.63761600	-1.37255700	-3.81251000
H	1.61964400	-3.89832400	-1.07213700
H	1.38107100	-0.83519100	4.64633500
C	1.10087300	-8.07241000	-3.94899000
H	0.07637300	-8.32200100	-4.21956500
H	1.73960400	-8.12045100	-4.82920200

H	1.46202900	-8.76378900	-3.18951400
C	2.52914000	-6.33239800	-3.03104000
H	3.16192300	-6.44285600	-3.91034800
H	2.53193500	-5.29733400	-2.69423400
H	2.86831000	-6.99730900	-2.23895000
C	0.26310400	-6.62096200	-2.19295900
H	0.25618000	-5.60893100	-1.79827300
H	-0.74790700	-6.90821900	-2.47616900
H	0.65026300	-7.30621900	-1.44116400
C	0.63924400	-5.73044400	-4.42360600
H	1.24993800	-5.83012900	-5.31968400
H	-0.39897600	-5.96393800	-4.65294700
H	0.73245100	-4.72405700	-4.01607800
N	1.13295100	-6.69404800	-3.40051800

[**1**$_2$+Me$_4$N$_{endo}$+I$_{endo}$]

Energy = -5261.76350626 hartree

Number of imaginary frequencies (PBE0-D3/def2-SVP) = 0

XYZ

N	-2.91760500	-1.97249600	-3.71286800
C	-3.56258100	-2.80995300	-2.86585700
C	-4.55108900	-2.31130400	-2.03438600
C	-4.85644800	-0.95407400	-2.17435400
C	-4.21507000	-0.11182100	-3.04819800
C	-3.12961600	-0.62570600	-3.80699000
C	-4.51515300	1.35952000	-3.18880500
C	-6.00907000	1.66974500	-3.12480100
C	-6.37739300	3.13009200	-3.37312600
C	-7.87460900	3.32526400	-3.18743800
C	-5.16942600	-3.19221500	-0.97172900
C	-6.64662700	-2.89940300	-0.72899500
C	-7.33529100	-3.82999000	0.26620700
C	-8.77407700	-3.38409100	0.47948400
O	-3.15922600	-4.05630400	-2.92466300
O	-2.35455100	0.06356600	-4.51661200
C	-3.69995600	2.16705400	-2.20479000
C	-2.40623600	2.56583200	-2.51008500

N	-1.72366700	3.32258100	-1.60824800
C	-2.17901800	3.68581300	-0.36538100
C	-3.47624700	3.24392300	-0.01360800
C	-4.17751800	2.51106000	-0.94262200
O	-1.74019700	2.27508600	-3.58990200
O	-1.40584600	4.34470000	0.38806100
C	-3.98791200	3.52215100	1.38336400
C	-5.50743600	3.63289600	1.47502200
C	-6.13135300	4.73090100	0.61719800
C	-7.64595500	4.69282600	0.75434300
C	-3.41759100	2.49050700	2.33229600
C	-2.22508000	2.73164300	2.98170800
N	-1.64201300	1.75432500	3.71594300
C	-2.11608100	0.47816000	3.83338200
C	-3.41470900	0.24469400	3.31007900
C	-3.99339300	1.23371700	2.55153800
O	-1.54516200	3.85954400	2.94435500
O	-1.36572400	-0.40313500	4.33865600
C	-4.03103800	-1.11290600	3.53924800
C	-5.52809800	-1.02758500	3.83804100
C	-6.19311100	-2.35799100	4.17999600
C	-7.68916200	-2.15497800	4.36606000
C	-3.71112500	-2.06789200	2.41408200
C	-2.57388900	-2.84070500	2.44167300
N	-2.35572100	-3.73872500	1.44747000
C	-3.16118600	-3.94141400	0.34587800
C	-4.31734700	-3.10193500	0.27370200
C	-4.54395000	-2.22036600	1.29183900
O	-1.62251600	-2.81345200	3.35461100
O	-2.82721400	-4.79775900	-0.48689600
C	-5.93250400	3.60956100	-4.74689000
C	-5.58891500	6.11019500	0.96100400
C	-5.57461400	-3.01173600	5.40674900
C	-7.27712000	-5.28667000	-0.16933600
H	-4.17089800	1.63606800	-4.18929100
H	-3.57734700	4.48826300	1.68713100
H	-3.55113800	-1.50629900	4.43955700
H	-5.09368500	-4.22169200	-1.33107200

H	-5.64805700	-0.54571500	-1.55477400
H	-5.43173700	-1.59870000	1.23989100
H	-4.95185200	1.02684700	2.08724000
H	-5.17543500	2.17810300	-0.67882800
H	-6.51001600	1.04263900	-3.87068000
H	-6.42633500	1.36445400	-2.15871800
H	-5.86308700	3.74403300	-2.62387500
H	-6.36998800	2.98893900	-5.53540500
H	-4.84707300	3.58177200	-4.86219000
H	-6.25282700	4.63979300	-4.91872300
H	-8.15596500	4.37287200	-3.31789800
H	-8.19741000	3.01404200	-2.19006500
H	-8.43742700	2.73650800	-3.91849200
H	-5.98026500	2.67938700	1.21487000
H	-5.76510100	3.81190500	2.52522200
H	-5.88362600	4.52773000	-0.43102500
H	-6.09647700	6.88026700	0.37562400
H	-4.52004500	6.19638500	0.75497000
H	-5.74821100	6.33856700	2.01981700
H	-7.94942600	4.90925100	1.78343500
H	-8.04672300	3.71043900	0.48939100
H	-8.11999200	5.43255500	0.10529900
H	-5.66286000	-0.33345400	4.67533700
H	-6.06282200	-0.57811700	2.99431900
H	-6.04923700	-3.03769300	3.33188600
H	-5.63183500	-2.34559700	6.27371600
H	-4.52556400	-3.27195400	5.25073700
H	-6.10366500	-3.93306300	5.66068800
H	-8.19361500	-3.10269000	4.56784400
H	-8.14633800	-1.71689300	3.47461800
H	-7.89032700	-1.48526400	5.20809000
H	-6.78485800	-1.86763500	-0.38664500
H	-7.16326200	-2.96126900	-1.69362000
H	-6.81193900	-3.74754700	1.22616800
H	-7.72987700	-5.41485300	-1.15779000
H	-7.82511200	-5.92091500	0.53142800
H	-6.25321900	-5.66249200	-0.21806700
H	-8.82476900	-2.34464200	0.81610500

H	-9.27311500	-4.00283300	1.22891100
H	-9.34705200	-3.46030100	-0.44995900
H	-1.58966400	-1.92712100	3.82849300
H	-3.09282500	-4.45474600	-2.00031900
H	-0.75854900	3.58009800	-1.85907200
H	-0.67032100	1.92375300	4.02311600
H	-1.50322400	-4.28426800	1.48390900
H	-2.04257600	-2.33407700	-4.10461100
H	-2.06600600	1.37609900	-4.04062600
H	-1.53752800	4.21005700	2.00374500
N	2.81008300	-0.21957300	-4.03673500
C	2.83290400	1.04930500	-3.56206800
C	3.86503400	1.44933800	-2.74423300
C	4.77050300	0.45105800	-2.34214300
C	4.71716200	-0.84556700	-2.76755600
C	3.71538600	-1.20870200	-3.72120800
C	5.56166500	-1.97167500	-2.21850400
C	6.96803700	-1.54466500	-1.81433600
C	7.85686200	-2.66875100	-1.28870800
C	9.20017500	-2.10536400	-0.85020600
C	3.96087100	2.87914000	-2.27194600
C	5.40652500	3.37494900	-2.20174700
C	5.56397200	4.84959000	-1.84053800
C	7.03997600	5.19341800	-1.70746100
O	1.80683800	1.78920900	-3.95005000
O	3.59598900	-2.32683700	-4.24695100
C	4.77800800	-2.62118500	-1.09667800
C	3.82661500	-3.59389300	-1.35722700
N	3.03501000	-4.02880600	-0.34180100
C	3.08486800	-3.59997900	0.96046000
C	4.14909800	-2.71597400	1.26859100
C	4.91115300	-2.22901500	0.23523900
O	3.59934800	-4.17409900	-2.50504600
O	2.19888800	-3.98323300	1.76575400
C	4.34474200	-2.31627500	2.71012700
C	5.81601700	-2.14654000	3.08692200
C	6.67744700	-3.38998700	2.88178700
C	8.13225100	-3.06550600	3.18599400

C	3.51625200	-1.10259000	3.05625000
C	2.21440800	-1.24674200	3.50167400
N	1.50480600	-0.13982700	3.83982700
C	1.92982800	1.15330100	3.67915200
C	3.23648400	1.32962800	3.15665900
C	3.97677200	0.20351600	2.88576400
O	1.55956000	-2.37495000	3.61964900
O	1.12691200	2.08690900	3.96334700
C	3.69204100	2.73231200	2.81809500
C	5.20376200	2.92264000	2.89704800
C	5.68466900	4.33976200	2.59568400
C	7.20447000	4.38962200	2.63780700
C	3.11912000	3.11206800	1.46958900
C	1.88867200	3.72307800	1.37404900
N	1.32562000	3.93377500	0.16139100
C	1.87775400	3.56945300	-1.03634800
C	3.21929600	3.10090200	-0.97738600
C	3.76249900	2.84297800	0.25542500
O	1.15453100	4.13367600	2.38888700
O	1.17790300	3.63669400	-2.08037500
C	8.03966700	-3.78325900	-2.30805900
C	6.19673200	-4.56871300	3.71516300
C	5.07852600	5.36965700	3.53729000
C	4.87935200	5.76644200	-2.84350900
H	5.65467400	-2.70434000	-3.02442300
H	3.95300800	-3.13981300	3.31344700
H	3.24263400	3.39459400	3.56257400
H	3.45316300	3.49184400	-3.02152100
H	5.55197900	0.73153600	-1.64435400
H	4.76278000	2.42316900	0.29378000
H	4.98060600	0.33408300	2.49872200
H	5.68521000	-1.50571300	0.47073500
H	7.44522000	-1.09000300	-2.69001900
H	6.92946200	-0.75362300	-1.05662200
H	7.37022700	-3.10211200	-0.40664700
H	8.46336200	-3.39315500	-3.23908000
H	7.09677300	-4.27782900	-2.54929900
H	8.72180600	-4.54684500	-1.92691000

H	9.83404900	-2.88387600	-0.41928900
H	9.07800000	-1.31952000	-0.09911000
H	9.73686500	-1.67172600	-1.69989500
H	6.26507000	-1.32284100	2.52146500
H	5.86496200	-1.84557900	4.13994500
H	6.60987900	-3.67670400	1.82588100
H	6.86179400	-5.42620700	3.58961300
H	5.19267900	-4.89202500	3.43287700
H	6.18209800	-4.31233800	4.77954500
H	8.25581300	-2.77045500	4.23285400
H	8.49668200	-2.24441300	2.56243900
H	8.77497100	-3.93063800	3.00732200
H	5.53113600	2.63599700	3.90301500
H	5.71491100	2.23772500	2.21183900
H	5.36909600	4.59369500	1.57637600
H	5.30343600	5.12371100	4.58016800
H	3.99356100	5.43549600	3.43266300
H	5.48625300	6.36287400	3.33571100
H	7.57426200	5.38194800	2.36981300
H	7.64716300	3.66919300	1.94414200
H	7.57491500	4.15613200	3.64089100
H	5.97812800	2.78260000	-1.47928200
H	5.87472600	3.18903100	-3.17506000
H	5.09268900	5.00997500	-0.86330100
H	5.26998200	5.59904800	-3.85245000
H	5.05362700	6.81470700	-2.58997000
H	3.79838400	5.61372400	-2.87284300
H	7.53022600	4.56367200	-0.95974000
H	7.17732000	6.23545100	-1.40921600
H	7.56148400	5.04871100	-2.65876800
H	1.19698500	3.45317600	3.12698300
H	1.63863000	2.54860400	-3.32758200
H	2.25583900	-4.61880800	-0.61338800
H	0.53444700	-0.27513800	4.15492100
H	0.34221700	4.25663100	0.16830800
H	2.00392800	-0.52158600	-4.57388300
H	3.65977200	-3.50499800	-3.27260300
H	1.88938800	-3.07868800	2.94023500

C	0.63845300	-1.38343500	0.42004000
H	0.13053500	-1.75547000	1.30831400
H	0.67199100	-2.14440500	-0.36371800
H	1.64479800	-1.05369600	0.67665000
C	0.58210200	0.31101800	-1.31434000
H	0.61700100	-0.49010600	-2.05792900
H	0.02945600	1.16571900	-1.69616900
H	1.58751500	0.61203800	-1.02194400
C	-0.18099800	0.83177800	0.92345000
H	-0.76847900	1.66834200	0.55275400
H	-0.64821000	0.40894500	1.80716700
H	0.82998500	1.15985100	1.15798800
C	-1.48931100	-0.64533600	-0.48533500
H	-1.39503700	-1.43998700	-1.22967900
H	-1.99903300	-1.00161200	0.40882900
H	-2.02394800	0.20967100	-0.89668100
N	-0.11435000	-0.21596500	-0.11203000
I	0.29187000	-3.00755300	-2.94998100

[**1**$_2$+Me$_4$N$_{endo}$+I$_{exo}$]

Energy = -5261.80193992 hartree

Number of imaginary frequencies (PBE0-D3/def2-SVP) = 0

XYZ

N	1.13547800	-0.93820700	3.94882300
C	1.69875600	-2.02239000	3.36122300
C	2.86509700	-1.88157900	2.63867800
C	3.41988600	-0.59315000	2.59697500
C	2.83945400	0.51290200	3.18231100
C	1.59574900	0.34563300	3.84337200
C	3.40711500	1.91446200	3.07669500
C	4.93287400	1.93951100	3.10203500
C	5.55267500	3.33161900	3.03622900
C	7.06864900	3.20655600	3.01047400
C	3.44357000	-3.07658300	1.90787100
C	4.97001700	-3.08989500	1.92530900
C	5.61543200	-4.29371900	1.24646700
C	7.12894800	-4.15084600	1.30556000

O	1.01940700	-3.13538700	3.54436800
O	0.85985600	1.27090400	4.29844300
C	2.81788000	2.63134600	1.87916500
C	1.65812700	3.36417200	2.01998900
N	1.10200900	3.95742500	0.93457600
C	1.57836800	3.87266300	-0.34584900
C	2.80811500	3.18635800	-0.51214200
C	3.37364700	2.58389000	0.59145200
O	0.98526300	3.55469800	3.13457300
O	0.86724900	4.36839800	-1.26895800
C	3.39965000	3.09372700	-1.90508700
C	4.92534800	3.13025900	-1.92446900
C	5.55228000	4.34501900	-1.24788000
C	7.06779900	4.22515000	-1.30631000
C	2.83590800	1.88926900	-2.63099900
C	1.64699200	2.00940400	-3.32085400
N	1.08655200	0.91441700	-3.89093000
C	1.58060000	-0.35957700	-3.81524300
C	2.83777800	-0.50695600	-3.17592100
C	3.41492500	0.61127900	-2.60625800
O	0.93946100	3.10592100	-3.48342000
O	0.85806900	-1.29439700	-4.27263600
C	3.42503000	-1.90100200	-3.07720000
C	4.95082900	-1.90615200	-3.10953900
C	5.58886300	-3.29026700	-3.05054500
C	7.10320300	-3.14549900	-3.03248800
C	2.85125900	-2.63070100	-1.87910500
C	1.71069700	-3.39213900	-2.01738900
N	1.16967600	-3.99826700	-0.93135100
C	1.63154000	-3.88071000	0.35235400
C	2.85426500	-3.18028600	0.51447400
C	3.40814600	-2.57140000	-0.59285700
O	1.03321000	-3.59378700	-3.12873400
O	0.91534800	-4.35874500	1.28041900
C	5.08970200	4.23703300	4.16778300
C	5.07654200	5.66168700	-1.84315700
C	5.13165300	-4.19893200	-4.18182800
C	5.15990800	-5.61856700	1.83938300

H	3.05692400	2.44846700	3.96476400
H	3.04507600	3.97582300	-2.44625900
H	3.07801600	-2.43642100	-3.96567700
H	3.10356900	-3.96481300	2.44817400
H	4.34399100	-0.45499200	2.03760200
H	4.31839600	-1.98567500	-0.45775500
H	4.35270000	0.48833300	-2.06657400
H	4.29376100	2.01458200	0.45419600
H	5.25763200	1.44593600	4.02567300
H	5.34422100	1.34542900	2.28055700
H	5.24470300	3.79396400	2.09013400
H	5.32135900	3.79053900	5.14057900
H	4.01394300	4.42793800	4.13407800
H	5.59397900	5.20539600	4.12007000
H	7.54274000	4.18398500	2.88985300
H	7.39269400	2.56155600	2.19015000
H	7.43644800	2.77044000	3.94494800
H	5.34038100	2.23050200	-1.46042000
H	5.24223700	3.10394800	-2.97403000
H	5.26161400	4.33064700	-0.19048700
H	5.58660700	6.50609900	-1.37297200
H	4.00238300	5.81442000	-1.70964300
H	5.29021800	5.70155200	-2.91655200
H	7.41879200	4.25821100	-2.34287800
H	7.40087500	3.28026800	-0.87019100
H	7.54900800	5.04401300	-0.76543600
H	5.26535200	-1.40573900	-4.03310100
H	5.35812100	-1.30964100	-2.28778500
H	5.29191200	-3.75866600	-2.10388700
H	5.35152600	-3.74681700	-5.15476800
H	4.05882600	-4.40468300	-4.14237400
H	5.64935600	-5.16043500	-4.13964100
H	7.59058600	-4.11696400	-2.91687200
H	7.42317500	-2.49839400	-2.21222400
H	7.46036900	-2.70231300	-3.96776100
H	5.37049200	-2.18304400	1.46226600
H	5.28715700	-3.06006300	2.97465100
H	5.32505200	-4.28199100	0.18894200

H	5.37370500	-5.65683500	2.91280000
H	5.68311800	-6.45424900	1.36804200
H	4.08828300	-5.78777800	1.70528200
H	7.44762800	-3.20035900	0.87087300
H	7.62274300	-4.96148800	0.76363200
H	7.48003700	-4.18011000	2.34220900
H	1.04970900	-2.75970000	-3.69730200
H	1.07281800	-3.72264500	2.72492300
H	0.18908500	4.40017600	1.06934500
H	0.15749100	1.04138000	-4.30566500
H	0.25976400	-4.44590000	-1.06604100
H	0.21710700	-1.07301700	4.37898500
H	1.02044500	2.72115900	3.70643700
H	1.00877100	3.70910700	-2.67026800
N	-1.99666200	0.98651500	3.97444100
C	-2.53404400	2.05629500	3.33934400
C	-3.69613000	1.90756200	2.60537500
C	-4.25123600	0.62395700	2.58038000
C	-3.70345700	-0.46967500	3.20747400
C	-2.47585900	-0.29573000	3.90471400
C	-4.26655900	-1.86914900	3.09585100
C	-5.79325200	-1.89970600	3.08237100
C	-6.40706800	-3.29708100	3.05710400
C	-7.92221400	-3.19312100	2.96996600
C	-4.27789000	3.08509900	1.85469300
C	-5.80572700	3.08741000	1.85442900
C	-6.44892400	4.31235400	1.20913900
C	-7.96224700	4.15696400	1.20586500
O	-1.85289100	3.16673600	3.49184400
O	-1.78323500	-1.21987400	4.40506500
C	-3.65194100	-2.58767700	1.91347600
C	-2.48035400	-3.30546500	2.05537000
N	-1.91910100	-3.90141400	0.97461700
C	-2.41588200	-3.85303500	-0.30211600
C	-3.65267800	-3.17232700	-0.46800800
C	-4.20093700	-2.55186200	0.62812900
O	-1.81116200	-3.47496500	3.17043200
O	-1.73625300	-4.36356300	-1.23031600

C	-4.26124900	-3.10711900	-1.85269400
C	-5.78822100	-3.13043500	-1.84078600
C	-6.40908500	-4.35899600	-1.18066600
C	-7.92422900	-4.22379700	-1.16375800
C	-3.69831400	-1.92798800	-2.61568400
C	-2.53550200	-2.06313400	-3.34966100
N	-1.98868000	-0.97842900	-3.95116300
C	-2.46389700	0.30279100	-3.85319000
C	-3.70173800	0.46191700	-3.16919600
C	-4.25348000	-0.64345600	-2.56800600
O	-1.84642600	-3.16836000	-3.51376800
O	-1.77232000	1.23781800	-4.33361600
C	-4.29042700	1.85318600	-3.08375000
C	-5.81772900	1.85488200	-3.07122800
C	-6.45682200	3.24102600	-3.06549600
C	-7.97005300	3.11064600	-2.98056000
C	-3.69468500	2.60968400	-1.91754700
C	-2.55576300	3.37463600	-2.07336100
N	-2.00676100	3.99184300	-0.99710300
C	-2.47145100	3.89694200	0.28994800
C	-3.68650900	3.17968500	0.46584200
C	-4.22964400	2.55188600	-0.62676300
O	-1.90225000	3.56053700	-3.19642500
O	-1.78494500	4.39896800	1.21659900
C	-5.98577500	-4.13442200	4.25551500
C	-5.98552200	-5.65408700	-1.85766400
C	-6.04732300	4.07071100	-4.27329600
C	-6.03582000	5.60816200	1.89117700
H	-3.94337500	-2.39761400	3.99666800
H	-3.93133500	-4.00650200	-2.37912000
H	-3.97658700	2.36845400	-3.99549000
H	-3.95768800	3.98353900	2.38906300
H	-5.17003800	0.48321100	2.02199400
H	-5.13822300	1.97582600	-0.48408300
H	-5.17853300	-0.51606800	-2.01692600
H	-5.13213900	-2.01085000	0.49359200
H	-6.14798200	-1.36707500	3.97190900
H	-6.18332400	-1.33771600	2.22654200

H	-6.05318400	-3.80696100	2.15290300
H	-6.25399200	-3.63575900	5.19239800
H	-4.91033900	-4.32336000	4.27052100
H	-6.48592200	-5.10532300	4.24195000
H	-8.38181700	-4.18129900	2.89733600
H	-8.23377900	-2.61478300	2.09564600
H	-8.33089000	-2.70096200	3.85790300
H	-6.18402200	-2.23709500	-1.34505400
H	-6.13272500	-3.06921200	-2.87942200
H	-6.06373300	-4.39666900	-0.14070700
H	-6.48977900	-6.50932700	-1.40238300
H	-4.91082000	-5.83091100	-1.77837000
H	-6.24750400	-5.64138100	-2.92048300
H	-8.32434400	-4.19230500	-2.18191700
H	-8.23838200	-3.30868300	-0.65402300
H	-8.38975200	-5.06809100	-0.65047000
H	-6.16273900	1.30265700	-3.95262800
H	-6.19739500	1.29903700	-2.20681700
H	-6.11464400	3.76826700	-2.16662800
H	-6.30373900	3.55541400	-5.20448000
H	-4.97544200	4.27906900	-4.28781100
H	-6.56517300	5.03237500	-4.27345800
H	-8.44760600	4.09122100	-2.92226100
H	-8.27326200	2.53848500	-2.09924400
H	-8.36752800	2.59938700	-3.86277100
H	-6.19266700	2.19288000	1.35398900
H	-6.14147700	3.01258000	2.89501200
H	-6.11454600	4.36167000	0.16603100
H	-6.28697500	5.58453600	2.95641800
H	-6.55581200	6.45982400	1.44704400
H	-4.96435200	5.79983400	1.80266400
H	-8.26902500	3.24173700	0.69179900
H	-8.44393000	4.99898700	0.70389300
H	-8.35203000	4.11202900	2.22751200
H	-1.91904500	2.71960900	-3.75342800
H	-1.90425500	3.74485900	2.66672800
H	-0.97974300	-4.30751400	1.10632700
H	-1.04513100	-1.10614300	-4.34618800

H	-1.07334000	4.40611100	-1.13011300
H	-1.05440000	1.11444400	4.37163800
H	-1.87924700	-2.65660800	3.76054700
H	-1.88038100	-3.74427800	-2.68441500
C	-0.63559000	-1.12383300	-0.97767000
H	-0.17539600	-0.95591400	-1.94758200
H	-0.29743400	-2.07283100	-0.57508300
H	-1.72058400	-1.11852200	-1.06137200
C	-0.80514300	-0.29910800	1.28079300
H	-0.45231300	-1.26288400	1.64068400
H	-0.47398300	0.48935500	1.95140500
H	-1.89075600	-0.30056100	1.20473100
C	-0.71864000	1.25198200	-0.55775500
H	-0.41063700	2.02614700	0.13929400
H	-0.28598900	1.44162400	-1.53721700
H	-1.80402500	1.21173300	-0.62691800
C	1.25051800	-0.01200800	0.05463100
H	1.59641400	-0.96919400	0.43496800
H	1.67453400	0.17258200	-0.92820500
H	1.53154900	0.78312600	0.73949600
N	-0.22567100	-0.04701500	-0.05455100
I	6.41189000	0.02672600	-0.00832200

[$\mathbf{1}_2$+Me_4N_{endo}+$2I_{exo}$]$^-$

Energy = -5559.697982 hartree

Number of imaginary frequencies (PBE0-D3/def2-SVP) = 0

XYZ

N	1.54477600	-0.95008100	3.93044300
C	2.10889900	-2.03228100	3.34033300
C	3.28170800	-1.89254800	2.62594900
C	3.83999400	-0.60648500	2.58827600
C	3.26500600	0.49571600	3.18336000
C	2.02074900	0.33010700	3.84623700
C	3.83338400	1.89642500	3.08430800
C	5.35922400	1.91942600	3.10450200
C	5.98152700	3.31048300	3.04243100
C	7.49730500	3.18416600	3.01089300

C	3.86139800	-3.08486800	1.89247500
C	5.38777100	-3.09634200	1.91099900
C	6.03525800	-4.29776800	1.23005300
C	7.54869100	-4.15333400	1.28788800
O	1.43047300	-3.14593600	3.51528100
O	1.30472500	1.25747400	4.32266300
C	3.24134700	2.61801300	1.89152400
C	2.07962600	3.34906700	2.03483000
N	1.52146300	3.94614100	0.95262200
C	2.00636700	3.87461100	-0.32581000
C	3.23436200	3.18322500	-0.49408900
C	3.79557300	2.56997700	0.60410200
O	1.41227300	3.53481200	3.15265200
O	1.31127800	4.38683400	-1.24941700
C	3.82323100	3.09534900	-1.88738000
C	5.34906600	3.12604000	-1.90803000
C	5.98153100	4.33603700	-1.22824000
C	7.49664500	4.21086400	-1.28653800
C	3.25568800	1.89590700	-2.61746100
C	2.06456300	2.01911800	-3.30497700
N	1.50327400	0.92855900	-3.88230800
C	2.00711100	-0.34303300	-3.82429100
C	3.26114900	-0.49348600	-3.17700900
C	3.83349600	0.61834800	-2.59378400
O	1.36188400	3.11887900	-3.46037500
O	1.30211200	-1.27700700	-4.30482000
C	3.84551900	-1.88805100	-3.08274500
C	5.37140400	-1.89460800	-3.11035900
C	6.00894500	-3.27898500	-3.05417100
C	7.52341100	-3.13618600	-3.03037900
C	3.26747400	-2.61952500	-1.88844600
C	2.11836300	-3.36982400	-2.02800500
N	1.57364800	-3.97804700	-0.94529300
C	2.05013700	-3.88347800	0.33539000
C	3.27373500	-3.18259700	0.49886800
C	3.82510200	-2.56483200	-0.60331000
O	1.44282100	-3.56036700	-3.14116400
O	1.35179900	-4.38202600	1.26360700

C	5.52319100	4.21225900	4.17879600
C	5.51078500	5.65658100	-1.81897400
C	5.55438500	-4.18366400	-4.18973100
C	5.58182400	-5.62453800	1.82037000
H	3.48496400	2.42559800	3.97591700
H	3.46987700	3.98045000	-2.42433800
H	3.49880400	-2.41912800	-3.97392500
H	3.52014000	-3.97460700	2.42944400
H	4.76451200	-0.46842300	2.03048800
H	4.73619800	-1.98174400	-0.46633800
H	4.76855300	0.49233300	-2.05094900
H	4.71343100	1.99840800	0.46447200
H	5.68715500	1.42084500	4.02459400
H	5.76839700	1.32863400	2.27947300
H	5.67005800	3.77669600	2.09966700
H	5.75704400	3.76119000	5.14920600
H	4.44749800	4.40262300	4.14832900
H	6.02817200	5.18077600	4.13314400
H	7.97240000	4.16167400	2.89106000
H	7.81694900	2.54024200	2.18784500
H	7.86762100	2.74469800	3.94316500
H	5.76241300	2.22416500	-1.44644800
H	5.66611200	3.10069000	-2.95779600
H	5.68978800	4.31930100	-0.17140100
H	6.02393300	6.49790900	-1.34575500
H	4.43720200	5.81107200	-1.68504000
H	5.72425300	5.69865100	-2.89255200
H	7.84773400	4.24696200	-2.32330300
H	7.82516000	3.26215600	-0.85500800
H	7.98117600	5.02564600	-0.74163600
H	5.68987300	-1.39065700	-4.03089600
H	5.77811200	-1.30154400	-2.28572600
H	5.70756400	-3.75013200	-2.11057300
H	5.77745800	-3.72807400	-5.16056800
H	4.48114000	-4.38645600	-4.15343600
H	6.07063900	-5.14643000	-4.14894500
H	8.00971700	-4.10871400	-2.91517500
H	7.84034600	-2.49058500	-2.20759900

H	7.88402800	-2.69074100	-3.96361700
H	5.78886400	-2.18894400	1.44941700
H	5.70542600	-3.06724800	2.96043600
H	5.74308000	-4.28430100	0.17325700
H	5.79618300	-5.66425400	2.89384800
H	6.10556200	-6.45901300	1.34661000
H	4.51026300	-5.79285100	1.68687600
H	7.86492600	-3.20047900	0.85632500
H	8.04339700	-4.96183500	0.74275600
H	7.90047100	-4.18510200	2.32456100
H	1.46286000	-2.72272000	-3.70843000
H	1.48306300	-3.73137000	2.69248300
H	0.59331700	4.36661600	1.08352800
H	0.56058300	1.05297900	-4.27764500
H	0.64631300	-4.39930300	-1.07637500
H	0.61156000	-1.08016900	4.33917900
H	1.44365400	2.69639200	3.72033900
H	1.42984300	3.71761300	-2.64268900
N	-1.57827400	0.97381600	3.99150000
C	-2.11642300	2.04490800	3.35820300
C	-3.27109500	1.89692400	2.61803400
C	-3.82951600	0.61178400	2.58048200
C	-3.27581600	-0.48129300	3.21115000
C	-2.05137700	-0.30912500	3.90849000
C	-3.83951100	-1.88122300	3.09743000
C	-5.36561300	-1.90742200	3.11486700
C	-5.98471700	-3.29930800	3.04142200
C	-7.50072800	-3.17644000	3.00850000
C	-3.84589900	3.08361800	1.87597900
C	-5.37295000	3.09910800	1.89948400
C	-6.01971200	4.29791900	1.21332900
C	-7.53330500	4.15868100	1.27911700
O	-1.43273100	3.15857200	3.51938300
O	-1.34286400	-1.23342700	4.40090500
C	-3.24169300	-2.58655800	1.89854300
C	-2.07720700	-3.31063200	2.03889800
N	-1.51300400	-3.90115600	0.95654300
C	-2.00280100	-3.83714300	-0.32075500

C	-3.23635000	-3.15541700	-0.48537300
C	-3.79337200	-2.53334400	0.61076900
O	-1.40526700	-3.49196900	3.15656500
O	-1.30631600	-4.33925100	-1.24907000
C	-3.83724000	-3.09639700	-1.87435600
C	-5.36351800	-3.12975200	-1.88312600
C	-5.98886000	-4.33039200	-1.18053300
C	-7.50458300	-4.20793600	-1.22722900
C	-3.27992800	-1.91096100	-2.63168800
C	-2.12811400	-2.04986700	-3.37652400
N	-1.57687300	-0.96523800	-3.97558700
C	-2.03853100	0.31861100	-3.85848200
C	-3.27107400	0.48059500	-3.17150900
C	-3.83196200	-0.62246600	-2.56670600
O	-1.44180300	-3.16140000	-3.55175300
O	-1.32778500	1.25162200	-4.32984600
C	-3.85719600	1.87384900	-3.08563700
C	-5.38381100	1.87368900	-3.10746500
C	-6.02547300	3.25629200	-3.05647400
C	-7.53944900	3.10931300	-3.02861700
C	-3.27941400	2.61791900	-1.90293100
C	-2.14569800	3.38597300	-2.05697200
N	-1.59440700	3.99974200	-0.97993000
C	-2.05145900	3.88965800	0.30707800
C	-3.26475800	3.17383000	0.48207700
C	-3.81899900	2.54639600	-0.61051800
O	-1.48659200	3.57826500	-3.18183000
O	-1.34594700	4.38143900	1.23286400
C	-5.52609300	-4.20870800	4.17160200
C	-5.52199800	-5.65885900	-1.75651300
C	-5.57608200	4.15708700	-4.19723800
C	-5.55933600	5.62735200	1.79223500
H	-3.49305300	-2.41872900	3.98457500
H	-3.48715000	-3.99154600	-2.39579100
H	-3.51737400	2.39686800	-3.98398300
H	-3.50258400	3.97630500	2.40652900
H	-4.73757700	0.46628800	1.99781800
H	-4.72023400	1.95057700	-0.46419000

H	-4.74359300	-0.48695800	-1.98723000
H	-4.71449100	-1.96705500	0.47167000
H	-5.69555200	-1.41684300	4.03855600
H	-5.77508700	-1.31081100	2.29415800
H	-5.67049000	-3.75758700	2.09582200
H	-5.76126400	-3.76509600	5.14514100
H	-4.45015300	-4.39756700	4.14056200
H	-6.02983400	-5.17750900	4.11839800
H	-7.97320300	-4.15407000	2.87938000
H	-7.82050600	-2.52616400	2.19052500
H	-7.87355200	-2.74600600	3.94401400
H	-5.77534000	-2.22116000	-1.43340100
H	-5.68753000	-3.12150400	-2.93104500
H	-5.68773300	-4.29850900	-0.12678700
H	-6.03062200	-6.49372900	-1.26718900
H	-4.44724900	-5.81089100	-1.62933700
H	-5.74433200	-5.71618300	-2.82759100
H	-7.86466000	-4.25982100	-2.26026300
H	-7.83053000	-3.25336300	-0.80687700
H	-7.98317300	-5.01520700	-0.66606900
H	-5.70369200	1.36316600	-4.02386800
H	-5.78547200	1.28382900	-2.27812200
H	-5.72318600	3.73234600	-2.11558600
H	-5.79978600	3.69615200	-5.16543000
H	-4.50340800	4.36352700	-4.16427500
H	-6.09546300	5.11833700	-4.15995400
H	-8.02816100	4.08105500	-2.91670000
H	-7.85260400	2.46646900	-2.20224300
H	-7.90082100	2.65872700	-3.95911300
H	-5.77836500	2.18988100	1.44537300
H	-5.68683000	3.07760600	2.95026100
H	-5.73254000	4.27599300	0.15533100
H	-5.76813100	5.67508300	2.86650000
H	-6.08307200	6.46009600	1.31539800
H	-4.48795200	5.79168300	1.65227000
H	-7.85427900	3.20366200	0.85589400
H	-8.02791400	4.96481800	0.73035400
H	-7.88022600	4.19907700	2.31714300

H	-1.49611000	2.73516400	-3.73448100
H	-1.47934200	3.72872300	2.68899200
H	-0.58497500	-4.32006800	1.09051100
H	-0.64402300	-1.09651100	-4.38247600
H	-0.67010500	4.42310400	-1.11436400
H	-0.64495100	1.10335000	4.39794300
H	-1.46332400	-2.67123800	3.74348800
H	-1.46713500	-3.72603300	-2.71539500
C	-0.27654800	-1.09508600	-1.00818500
H	0.19259300	-0.90881400	-1.97009100
H	0.04712900	-2.05605300	-0.62268200
H	-1.36041700	-1.07823800	-1.10154000
C	-0.46094000	-0.31595900	1.26468000
H	-0.11524100	-1.28687200	1.61191000
H	-0.13838300	0.46148900	1.95174200
H	-1.54537100	-0.31405500	1.17675300
C	-0.34617500	1.27047800	-0.54043400
H	-0.04066100	2.02819300	0.17534600
H	0.09365300	1.47562100	-1.51354200
H	-1.43108700	1.23867900	-0.61838500
C	1.60910700	-0.01919800	0.06225700
H	1.94701900	-0.98597200	0.42561200
H	2.04349600	0.18289000	-0.91285900
H	1.89177500	0.75976400	0.76521700
N	0.13518900	-0.04144100	-0.05937300
I	6.92045300	0.02060800	-0.00765900
I	-6.95235800	-0.01477200	0.00653900

[**1**$_2$+I$_{exo}$]$^-$

Energy = -5047.69155803 hartree

Number of imaginary frequencies (PBE0-D3/def2-SVP) = 0

XYZ

N	1.05782700	0.93671500	-3.91660800
C	1.61871000	2.02022900	-3.33115700
C	2.79035800	1.88501100	-2.61893300
C	3.34591600	0.60116000	-2.56886600
C	2.77954100	-0.50021700	-3.17313500

C	1.53902100	-0.33936900	-3.83782300
C	3.36226000	-1.89225100	-3.07465500
C	4.88843400	-1.90093400	-3.09549100
C	5.52331200	-3.28624100	-3.03204300
C	7.03811900	-3.14787700	-3.00467700
C	3.36163900	3.07965100	-1.89041400
C	4.88813200	3.09963500	-1.90304200
C	5.52662900	4.30352000	-1.21813000
C	7.04117700	4.16884700	-1.26919800
O	0.94458800	3.13776500	-3.51647400
O	0.83213500	-1.26878000	-4.33597600
C	2.77594700	-2.60895300	-1.88011800
C	1.62242400	-3.34749600	-2.02310300
N	1.06381600	-3.94028700	-0.94195500
C	1.54096800	-3.85824400	0.33560000
C	2.76555800	-3.16655700	0.50293900
C	3.32013700	-2.54234200	-0.59248100
O	0.96747900	-3.55181700	-3.14925700
O	0.84558000	-4.37746600	1.26208500
C	3.36160400	-3.07965100	1.89047800
C	4.88809600	-3.09963300	1.90313700
C	5.52661000	-4.30351600	1.21823800
C	7.04115700	-4.16884000	1.26933600
C	2.79030500	-1.88501200	2.61898400
C	1.61864400	-2.02023100	3.33118500
N	1.05774800	-0.93671800	3.91662600
C	1.53894400	0.33936600	3.83785300
C	2.77947500	0.50021700	3.17318700
C	3.34586300	-0.60115900	2.56892900
O	0.94451900	-3.13776800	3.51648900
O	0.83204700	1.26877500	4.33599400
C	3.36219400	1.89225200	3.07471900
C	4.88836700	1.90093700	3.09558700
C	5.52324500	3.28624500	3.03215300
C	7.03805200	3.14788400	3.00481700
C	2.77590500	2.60895300	1.88016900
C	1.62238000	3.34749700	2.02313100
N	1.06379100	3.94028300	0.94196900

C	1.54096700	3.85823500	-0.33557600
C	2.76556300	3.16655400	-0.50288900
C	3.32012100	2.54234200	0.59254400
O	0.96741500	3.55182500	3.14927200
O	0.84559700	4.37745500	-1.26207600
C	5.06981800	-4.19538500	-4.16464300
C	5.06761400	-5.62877600	1.80795600
C	5.06972700	4.19538700	4.16474400
C	5.06764300	5.62877900	-1.80785700
H	3.01896300	-2.42761100	-3.96460800
H	3.01737500	-3.96702700	2.42958700
H	3.01887800	2.42761100	3.96466500
H	3.01741900	3.96702600	-2.42953000
H	4.26770600	0.46475700	-2.00741600
H	4.23283700	1.96417900	0.45130100
H	4.26766400	-0.46475600	2.00749800
H	4.23284900	-1.96417800	-0.45121900
H	5.21273500	-1.39971000	-4.01573400
H	5.29434500	-1.30704700	-2.27105000
H	5.21691400	-3.75193900	-2.08756000
H	5.29613800	-3.74413800	-5.13702600
H	3.99593800	-4.39446300	-4.12933000
H	5.58440500	-5.15919900	-4.11830900
H	7.52176100	-4.12112600	-2.88125300
H	7.35387400	-2.49679500	-2.18566200
H	7.40246100	-2.70956300	-3.94014800
H	5.29426500	-2.19425700	1.44193700
H	5.21060200	-3.07335000	2.95143100
H	5.22873300	-4.28650400	0.16328200
H	5.58440000	-6.46585900	1.33037300
H	3.99430800	-5.78846200	1.67859400
H	5.28617800	-5.67142200	2.88071100
H	7.39737700	-4.20610100	2.30458000
H	7.36057300	-3.21588000	0.84012600
H	7.52877700	-4.97855700	0.71894900
H	5.21265000	1.39971500	4.01583700
H	5.29429600	1.30705000	2.27115500
H	5.21686400	3.75194400	2.08766400

H	5.29602800	3.74414100	5.13713100
H	3.99584600	4.39446400	4.12940900
H	5.58431200	5.15920300	4.11842100
H	7.52169500	4.12113400	2.88140400
H	7.35382500	2.49680400	2.18580700
H	7.40237700	2.70957000	3.94029400
H	5.29429300	2.19426000	-1.44183400
H	5.21065900	3.07335300	-2.95133000
H	5.22873100	4.28650700	-0.16318000
H	5.28622800	5.67142600	-2.88060800
H	5.58441700	6.46586300	-1.33026400
H	3.99433400	5.78846200	-1.67851700
H	7.36058700	3.21588800	-0.83998100
H	7.52878400	4.97856500	-0.71880000
H	7.39741800	4.20610900	-2.30443400
H	0.97985300	2.71069600	3.71083400
H	0.97968000	3.71361200	-2.68578400
H	0.14003800	-4.36212000	-1.07690400
H	0.12494000	-1.06896000	4.32054000
H	0.14001000	4.36211600	1.07690000
H	0.12502700	1.06895700	-4.32054200
H	0.97992700	-2.71068400	-3.71081300
H	0.97963100	-3.71361900	2.68580200
N	-1.94685400	-0.94874800	-3.89147700
C	-2.50567900	-2.03012700	-3.30156200
C	-3.69163500	-1.89941100	-2.60323700
C	-4.25134700	-0.62011800	-2.56916400
C	-3.69477800	0.48065100	-3.17421300
C	-2.45077400	0.32164500	-3.84253100
C	-4.27064900	1.87452200	-3.07686100
C	-5.79726300	1.89506800	-3.07149800
C	-6.41968100	3.28837800	-3.04742100
C	-7.93479600	3.17876400	-2.96611900
C	-4.26614700	-3.08778900	-1.86673900
C	-5.79303300	-3.10483900	-1.86533600
C	-6.42320300	-4.33400700	-1.21597300
C	-7.93829700	-4.19696800	-1.20876500
O	-1.83239700	-3.14360100	-3.47079000

O	-1.78069300	1.25419500	-4.36438000
C	-3.66790300	2.60309000	-1.89707600
C	-2.50280100	3.33153900	-2.03924800
N	-1.94442600	3.92860400	-0.96044400
C	-2.44188100	3.87542100	0.31322900
C	-3.66824800	3.17874700	0.48089400
C	-4.21086900	2.55102700	-0.61235700
O	-1.85353600	3.52112300	-3.16412500
O	-1.78242000	4.41659500	1.24207100
C	-4.26618500	3.08778800	1.86665800
C	-5.79307000	3.10483900	1.86522500
C	-6.42322800	4.33400600	1.21584800
C	-7.93832200	4.19696600	1.20860800
C	-3.69168700	1.89941100	2.60316700
C	-2.50574400	2.03012700	3.30151300
N	-1.94693100	0.94875000	3.89144200
C	-2.45085100	-0.32164400	3.84248900
C	-3.69484000	-0.48065000	3.17414400
C	-4.25139900	0.62011800	2.56908300
O	-1.83246500	3.14360200	3.47075200
O	-1.78078200	-1.25419300	4.36435400
C	-4.27070900	-1.87452200	3.07678000
C	-5.79732200	-1.89506900	3.07138500
C	-6.41973900	-3.28838000	3.04729500
C	-7.93485200	-3.17876600	2.96596000
C	-3.66793800	-2.60309100	1.89700700
C	-2.50284100	-3.33154200	2.03920200
N	-1.94444600	-3.92860800	0.96040900
C	-2.44187500	-3.87542400	-0.31327500
C	-3.66823700	-3.17874800	-0.48096400
C	-4.21087900	-2.55102800	0.61227700
O	-1.85359500	-3.52112700	3.16409100
O	-1.78239600	-4.41659900	-1.24210200
C	-5.99735700	4.13052700	-4.24202500
C	-5.99694700	5.62784900	1.89338500
C	-5.99744000	-4.13052800	4.24190800
C	-5.99690700	-5.62785000	-1.89350000
H	-3.94414300	2.39864300	-3.97927400

H	-3.93510400	3.97853700	2.40750000
H	-3.94422100	-2.39864400	3.97920000
H	-3.93505600	-3.97853800	-2.40757600
H	-5.18328000	-0.49014900	-2.03003100
H	-5.12926100	-1.98891100	0.47423000
H	-5.18332000	0.49014800	2.02993100
H	-5.12925500	1.98891200	-0.47432900
H	-6.14625800	1.35940800	-3.96206400
H	-6.18718300	1.33241000	-2.21627900
H	-6.07010300	3.79741400	-2.14147400
H	-6.25900800	3.63181900	-5.18118500
H	-4.92222300	4.31984200	-4.25005200
H	-6.50078400	5.10043600	-4.22860000
H	-8.39993000	4.16517100	-2.89550300
H	-8.24586700	2.59905900	-2.09245500
H	-8.33781600	2.68307200	-3.85523900
H	-6.18753300	2.21386900	1.36459900
H	-6.13225300	3.03427400	2.90547800
H	-6.08402200	4.37565900	0.17434200
H	-6.50532000	6.48484400	1.44453400
H	-4.92271500	5.80200400	1.80630600
H	-6.24990800	5.61040300	2.95870600
H	-8.33101400	4.15810000	2.22988000
H	-8.25312000	3.28343900	0.69629400
H	-8.40995800	5.04317300	0.70280400
H	-6.14633600	-1.35940900	3.96194400
H	-6.18722500	-1.33241100	2.21615900
H	-6.07014100	-3.79741500	2.14135500
H	-6.25911200	-3.63182100	5.18106200
H	-4.92230600	-4.31984300	4.24995800
H	-6.50086700	-5.10043800	4.22847200
H	-8.39998400	-4.16517400	2.89533300
H	-8.24590500	-2.59906000	2.09229000
H	-8.33789200	-2.68307500	3.85507200
H	-6.18750500	-2.21387000	-1.36471800
H	-6.13219500	-3.03427500	-2.90559700
H	-6.08401800	-4.37566000	-0.17446000
H	-6.24984600	-5.61040400	-2.95882700

H	-6.50528900	-6.48484500	-1.44466000
H	-4.92267700	-5.80200400	-1.80639900
H	-8.25310600	-3.28344100	-0.69645700
H	-8.40994300	-5.04317400	-0.70296900
H	-8.33096800	-4.15810200	-2.23004400
H	-1.87714800	-2.68164700	3.73087900
H	-1.87478800	-3.72944200	-2.64597800
H	-0.99206100	4.31391900	-1.08741800
H	-0.98771000	1.07312200	4.26308600
H	-0.99208600	-4.31392600	1.08740200
H	-0.98762600	-1.07312000	-4.26310500
H	-1.87708800	2.68164600	-3.73091900
H	-1.87483800	3.72943900	2.64593600
I	6.54935500	0.00000300	0.00006500

[**1**$_2$+2I$_{exo}$]$^{2-}$

Energy = -5345.51931498 hartree

Number of imaginary frequencies (PBE0-D3/def2-SVP) = 0

XYZ

N	1.50753200	-0.94408000	3.90675600
C	2.06886000	-2.02545300	3.31760700
C	3.24598100	-1.89086800	2.61149400
C	3.80443200	-0.60869400	2.56563500
C	3.24178900	0.48952900	3.17661900
C	2.00149100	0.32889800	3.84497300
C	3.82201000	1.88243500	3.07983200
C	5.34833800	1.89250400	3.09707300
C	5.98278600	3.27810800	3.03538600
C	7.49780300	3.14191900	3.00590000
C	3.81920900	-3.08289800	1.87975800
C	5.34580300	-3.10043300	1.89471000
C	5.98743800	-4.30254100	1.20955200
C	7.50189100	-4.16680900	1.26233200
O	1.39598500	-3.14368000	3.49514300
O	1.31243300	1.25904100	4.36057900
C	3.23258500	2.60137100	1.88786000
C	2.07174600	3.33117800	2.03029500

N	1.51037600	3.92473100	0.95093100
C	1.99991100	3.85941300	-0.32377700
C	3.22611100	3.16735200	-0.49107900
C	3.77900200	2.53889600	0.60146000
O	1.41828200	3.52741400	3.15737900
O	1.32048800	4.39269200	-1.25078200
C	3.81920400	3.08290100	-1.87973400
C	5.34579900	3.10043800	-1.89467900
C	5.98742800	4.30254800	-1.20951700
C	7.50188100	4.16682000	-1.26228900
C	3.24598200	1.89086900	-2.61147200
C	2.06886600	2.02545200	-3.31759400
N	1.50755000	0.94407800	-3.90675200
C	2.00151300	-0.32890000	-3.84496700
C	3.24180400	-0.48952700	-3.17660000
C	3.80443800	0.60869700	-2.56561000
O	1.39598500	3.14367600	-3.49512700
O	1.31246000	-1.25904200	-4.36058000
C	3.82203000	-1.88243200	-3.07981000
C	5.34835800	-1.89249300	-3.09704500
C	5.98281200	-3.27809500	-3.03535400
C	7.49782800	-3.14189800	-3.00586500
C	3.23260400	-2.60137000	-1.88784000
C	2.07176600	-3.33118100	-2.03028000
N	1.51039400	-3.92473400	-0.95091800
C	1.99992700	-3.85941700	0.32379100
C	3.22612300	-3.16735100	0.49109900
C	3.77901600	-2.53889400	-0.60143900
O	1.41831100	-3.52742200	-3.15736700
O	1.32050600	-4.39270400	1.25079300
C	5.52929100	4.18503300	4.16977900
C	5.52879700	5.62914900	-1.79657500
C	5.52932600	-4.18502300	-4.16974800
C	5.52880800	-5.62914300	1.79660900
H	3.47883500	2.41453400	3.97184800
H	3.47417000	3.97170100	-2.41604900
H	3.47886000	-2.41453200	-3.97182700
H	3.47417400	-3.97169900	2.41607100

H	4.72642700	-0.47218000	2.00535700
H	4.69298100	-1.96345100	-0.46005000
H	4.72642700	0.47218600	-2.00532200
H	4.69296800	1.96345600	0.46007400
H	5.67575600	1.38897800	4.01523900
H	5.75406500	1.30095300	2.27078700
H	5.67385000	3.74490900	2.09251200
H	5.75660900	3.73196700	5.14135300
H	4.45506200	4.38110300	4.13477400
H	6.04248600	5.15009400	4.12449700
H	7.98042300	4.11616800	2.88240400
H	7.81288000	2.49113500	2.18618600
H	7.86363700	2.70307000	3.94092700
H	5.75237000	2.19475700	-1.43438100
H	5.66784100	3.07382100	-2.94335800
H	5.68980100	4.28433200	-0.15468400
H	6.04684600	6.46555100	-1.31824100
H	4.45575200	5.78769600	-1.66557200
H	5.74561000	5.67269100	-2.86989400
H	7.85675900	4.20593000	-2.29827700
H	7.82047800	3.21211500	-0.83596800
H	7.99114800	4.97512400	-0.71039700
H	5.67577800	-1.38896500	-4.01520900
H	5.75407800	-1.30094200	-2.27075600
H	5.67387600	-3.74489700	-2.09248100
H	5.75664500	-3.73195800	-5.14132100
H	4.45509700	-4.38109900	-4.13474700
H	6.04252300	-5.15008300	-4.12446300
H	7.98045300	-4.11614600	-2.88236800
H	7.81290000	-2.49111400	-2.18614900
H	7.86366200	-2.70304600	-3.94089000
H	5.75237600	-2.19475000	1.43441400
H	5.66784000	-3.07381500	2.94339100
H	5.68981700	-4.28432700	0.15471700
H	5.74561700	-5.67268400	2.86992800
H	6.04686100	-6.46554400	1.31827700
H	4.45576400	-5.78769100	1.66560100
H	7.82048700	-3.21210400	0.83601000

H	7.99116300	-4.97511200	0.71044400
H	7.85676400	-4.20591600	2.29832200
H	1.43321900	-2.68422500	-3.71911600
H	1.43041700	-3.71770700	2.66140100
H	0.57018600	4.32192800	1.08060800
H	0.56034900	1.07132800	-4.28986800
H	0.57020800	-4.32193900	-1.08060000
H	0.56032500	-1.07133300	4.28986000
H	1.43318300	2.68421000	3.71911700
H	1.43040600	3.71769400	-2.66137800
N	-1.50756400	0.94461800	3.90665100
C	-2.06887600	2.02590700	3.31733400
C	-3.24598100	1.89122200	2.61121400
C	-3.80443000	0.60904100	2.56551800
C	-3.24180100	-0.48909500	3.17667000
C	-2.00151500	-0.32837200	3.84502800
C	-3.82202400	-1.88201300	3.08006800
C	-5.34835200	-1.89207900	3.09730400
C	-5.98279800	-3.27769300	3.03580100
C	-7.49781500	-3.14151100	3.00628300
C	-3.81920500	3.08315200	1.87931100
C	-5.34579900	3.10069100	1.89426000
C	-5.98743400	4.30270500	1.20893300
C	-7.50188700	4.16697700	1.26172700
O	-1.39600300	3.14416000	3.49472300
O	-1.31246400	-1.25844400	4.36076800
C	-3.23259500	-2.60111000	1.88819700
C	-2.07175300	-3.33089100	2.03073300
N	-1.51037400	-3.92458600	0.95145200
C	-1.99990800	-3.85944300	-0.32326600
C	-3.22611200	-3.16741100	-0.49066500
C	-3.77900800	-2.53880900	0.60178700
O	-1.41829300	-3.52697600	3.15784600
O	-1.32048700	-4.39284500	-1.25020000
C	-3.81920000	-3.08315500	-1.87933300
C	-5.34579400	-3.10069800	-1.89428600
C	-5.98742600	-4.30271000	-1.20895300
C	-7.50187900	-4.16698500	-1.26174700

C	-3.24597400	-1.89122800	-2.61123600
C	-2.06886200	-2.02591200	-3.31734500
N	-1.50754300	-0.94462200	-3.90665300
C	-2.00149400	0.32836800	-3.84503100
C	-3.24178500	0.48909200	-3.17668400
C	-3.80442100	-0.60904500	-2.56554200
O	-1.39598800	-3.14416600	-3.49473100
O	-1.31243900	1.25843900	-4.36076600
C	-3.82200800	1.88201200	-3.08009000
C	-5.34833500	1.89207900	-3.09733800
C	-5.98278100	3.27769200	-3.03583800
C	-7.49779900	3.14150900	-3.00633000
C	-3.23258700	2.60111200	-1.88821700
C	-2.07174300	3.33089300	-2.03074800
N	-1.51037000	3.92458800	-0.95146500
C	-1.99990900	3.85944100	0.32325100
C	-3.22611500	3.16741100	0.49064400
C	-3.77900800	2.53881400	-0.60181000
O	-1.41827900	3.52697700	-3.15785900
O	-1.32049000	4.39283900	1.25018900
C	-5.52931200	-4.18446100	4.17032300
C	-5.52879900	-5.62939500	-1.79582300
C	-5.52928800	4.18446000	-4.17035700
C	-5.52880900	5.62938800	1.79581000
H	-3.47885300	-2.41399100	3.97215800
H	-3.47416200	-3.97203100	-2.41552100
H	-3.47882900	2.41398600	-3.97218000
H	-3.47416900	3.97202600	2.41550200
H	-4.72641000	0.47244500	2.00523700
H	-4.69298000	1.96340100	-0.46035100
H	-4.72640700	-0.47245300	-2.00527000
H	-4.69297700	-1.96339300	0.46032200
H	-5.67577500	-1.38842800	4.01539900
H	-5.75407500	-1.30064100	2.27093500
H	-5.67385300	-3.74462300	2.09299400
H	-5.75664200	-3.73126300	5.14183200
H	-4.45508100	-4.38053100	4.13535700
H	-6.04250100	-5.14953100	4.12516700

H	-7.98043100	-4.11577900	2.88292300
H	-7.81288700	-2.49084600	2.18647200
H	-7.86365700	-2.70252700	3.94124300
H	-5.75237000	-2.19495000	-1.43412100
H	-5.66783000	-3.07423100	-2.94297000
H	-5.68980100	-4.28434400	-0.15412200
H	-6.04685400	-6.46572700	-1.31737200
H	-4.45575500	-5.78792900	-1.66479500
H	-5.74561100	-5.67308700	-2.86913600
H	-7.85675400	-4.20624300	-2.29773100
H	-7.82047400	-3.21221700	-0.83556500
H	-7.99114800	-4.97520800	-0.70974000
H	-5.67575200	1.38842800	-4.01543500
H	-5.75406400	1.30064000	-2.27097200
H	-5.67384300	3.74462300	-2.09302900
H	-5.75660900	3.73126100	-5.14186700
H	-4.45505800	4.38053300	-4.13538100
H	-6.04247900	5.14953000	-4.12520700
H	-7.98041600	4.11577700	-2.88297100
H	-7.81287500	2.49084200	-2.18652300
H	-7.86363500	2.70252800	-3.94129400
H	-5.75237200	2.19494500	1.43409000
H	-5.66783800	3.07421800	2.94294300
H	-5.68980900	4.28434600	0.15410200
H	-5.74562000	5.67307500	2.86912300
H	-6.04686500	6.46572200	1.31736300
H	-4.45576500	5.78792400	1.66478200
H	-7.82047900	3.21221100	0.83554000
H	-7.99115800	4.97520200	0.70972500
H	-7.85676200	4.20622900	2.29771100
H	-1.43319500	2.68370900	-3.71949500
H	-1.43041400	3.71805800	2.66089400
H	-0.57017800	-4.32175200	1.08118500
H	-0.56034400	-1.07192900	-4.28975400
H	-0.57016700	4.32174600	-1.08118600
H	-0.56036600	1.07192300	4.28975500
H	-1.43321200	-2.68371300	3.71948700
H	-1.43041300	-3.71806900	-2.66090700

I	7.09478200	0.00000200	0.00001500
I	-7.09480700	0.00000000	-0.00002400

2

Energy = -2310.59927226 hartree

Number of imaginary frequencies (PBE0-D3/def2-SVP) = 0

C	1.226051	-3.497467	-1.828006
C	1.227514	-2.887412	-0.558308
C	-0.007429	-2.604321	0.028449
C	-1.234109	-2.898958	-0.565315
C	-1.194154	-3.526153	-1.816623
C	-2.571936	-2.530404	0.040575
C	-2.575856	-2.522569	1.572220
C	-3.930294	-2.201684	2.208130
C	-3.808473	-2.199980	3.727468
C	2.546117	-2.533706	0.103374
C	2.487482	-2.510537	1.633420
C	3.823857	-2.223547	2.320982
C	3.632359	-2.154732	3.831588
O	2.340821	-3.813935	-2.506944
C	-3.092249	-1.220038	-0.527557
C	-4.131503	-1.188585	-1.475187
C	-4.161882	1.227665	-1.369075
C	-3.101344	1.235974	-0.456731
C	-2.586792	-0.001696	-0.077802
O	-4.664210	-2.308255	-2.005100
C	-2.546117	2.533706	0.103372
C	-2.487482	2.510538	1.633418
C	-3.823857	2.223550	2.320981
C	-3.632359	2.154736	3.831587
C	-1.227514	2.887411	-0.558311
C	-1.226050	3.497465	-1.828009
C	1.194154	3.526152	-1.816626
C	1.234109	2.898958	-0.565317
C	0.007429	2.604321	0.028447
O	-2.340821	3.813932	-2.506948
C	2.571936	2.530404	0.040574

C	2.575856	2.522570	1.572218
C	3.930294	2.201684	2.208129
C	3.808472	2.199981	3.727467
C	3.092249	1.220038	-0.527557
C	4.131503	1.188584	-1.475187
C	4.161882	-1.227666	-1.369075
C	3.101344	-1.235974	-0.456730
C	2.586792	0.001696	-0.077802
O	4.664211	2.308253	-2.005101
C	-5.035328	-3.142511	1.740502
C	-4.904743	3.231417	1.946028
C	5.035328	3.142510	1.740500
C	4.904744	-3.231414	1.946030
H	-3.277235	-3.317436	-0.263467
H	-3.254376	3.332409	-0.164713
H	3.277235	3.317436	-0.263469
H	3.254376	-3.332409	-0.164710
H	-0.014031	-2.132832	1.011591
H	1.754997	0.017608	0.631161
H	0.014031	2.132833	1.011589
H	-1.754997	-0.017607	0.631161
H	-2.228304	-3.510639	1.923258
H	-1.840198	-1.793961	1.952155
H	-4.207859	-1.179706	1.893336
H	-4.777071	-4.193643	1.955518
H	-5.225457	-3.053595	0.660274
H	-5.981848	-2.921603	2.257752
H	-4.757102	-1.909639	4.205061
H	-3.033470	-1.494673	4.067915
H	-3.537378	-3.200199	4.105142
H	-1.762689	1.751864	1.972718
H	-2.092597	3.481297	1.982202
H	-4.161446	1.228016	1.980598
H	-5.839071	3.024631	2.490594
H	-5.138872	3.206108	0.871225
H	-4.592080	4.258549	2.200583
H	-3.294189	3.124655	4.233374
H	-2.878937	1.399599	4.107137

H	-4.571206	1.891482	4.342952
H	2.228305	3.510640	1.923256
H	1.840198	1.793963	1.952154
H	4.207858	1.179706	1.893336
H	4.777072	4.193642	1.955516
H	5.225458	3.053594	0.660272
H	5.981848	2.921602	2.257750
H	4.757101	1.909640	4.205060
H	3.033469	1.494675	4.067914
H	3.537378	3.200200	4.105140
H	1.762689	-1.751863	1.972720
H	2.092598	-3.481295	1.982205
H	4.161445	-1.228013	1.980599
H	4.592082	-4.258546	2.200586
H	5.839072	-3.024627	2.490595
H	5.138872	-3.206106	0.871227
H	2.878937	-1.399596	4.107138
H	4.571206	-1.891477	4.342953
H	3.294190	-3.124651	4.233376
H	3.961533	2.980768	-2.071662
H	3.141311	-3.415936	-2.123837
H	-3.961531	-2.980769	-2.071662
H	-3.141311	3.415932	-2.123842
O	-4.666101	2.441354	-1.756678
H	-5.356805	2.311936	-2.415823
O	-2.387380	-3.837060	-2.411530
H	-2.225256	-4.235168	-3.273532
O	4.666099	-2.441355	-1.756679
H	5.356803	-2.311937	-2.415824
O	2.387380	3.837058	-2.411533
H	2.225256	4.235166	-3.273535
C	0.010875	-3.825309	-2.437775
H	0.039866	-4.310216	-3.417078
C	-4.664836	0.037701	-1.882558
H	-5.472448	0.032661	-2.619090
C	4.664835	-0.037702	-1.882559
H	5.472447	-0.032663	-2.619091
C	-0.010875	3.825307	-2.437778

H	-0.039866	4.310213	-3.417081

2$_2$

Energy = -4621.27735131 hartree

Number of imaginary frequencies (PBE0-D3/def2-SVP) = 0

C	-1.982953	2.624422	3.117096
C	-3.059137	2.304447	2.287625
C	-3.567664	1.014021	2.383068
C	-3.072866	0.043843	3.244180
C	-2.002286	0.412942	4.053585
C	-3.648152	-1.353962	3.322143
C	-5.178009	-1.355623	3.273700
C	-5.827685	-2.724113	3.457668
C	-7.339236	-2.596030	3.344229
C	-3.650723	3.333189	1.346339
C	-5.180514	3.292267	1.326185
C	-5.840262	4.387777	0.493533
C	-7.349057	4.193847	0.489017
O	-1.370802	3.842938	3.116712
C	-3.032798	-2.286562	2.298257
C	-1.971350	-3.130806	2.626311
C	-1.995987	-4.076273	0.417778
C	-3.050752	-3.250467	0.041536
C	-3.531041	-2.374477	1.004446
O	-1.370896	-3.136050	3.850490
C	-3.651066	-3.333020	-1.346118
C	-5.180847	-3.292223	-1.325483
C	-5.840194	-4.388045	-0.492925
C	-7.348986	-4.194114	-0.487587
C	-3.059741	-2.304136	-2.287416
C	-1.983227	-2.623782	-3.116552
C	-2.003148	-0.412461	-4.053306
C	-3.073932	-0.043545	-3.244128
C	-3.568686	-1.013874	-2.383117
O	-1.370353	-3.841951	-3.115821
C	-3.649183	1.354280	-3.322015
C	-5.179002	1.356077	-3.273180

C	-5.828574	2.724635	-3.457015
C	-7.340092	2.596779	-3.342925
C	-3.033454	2.286793	-2.298250
C	-1.971719	3.130672	-2.626411
C	-1.995595	4.075826	-0.417693
C	-3.050719	3.250503	-0.041428
C	-3.531513	2.374818	-1.004360
O	-1.371557	3.136010	-3.850706
C	-5.435122	-3.380920	4.772863
C	-5.474644	-5.783888	-0.976289
C	-5.436462	3.381244	-4.772445
C	-5.474478	5.783800	0.976192
H	-3.384970	-1.738607	4.310159
H	-3.388274	-4.314387	-1.747701
H	-3.386129	1.738913	-4.310076
H	-3.387907	4.314625	1.747718
H	-4.403643	0.749628	1.747497
H	-4.361898	1.731984	-0.735856
H	-4.404859	-0.749680	-1.747725
H	-4.361211	-1.731348	0.735987
H	-5.537116	-0.677794	4.056874
H	-5.531598	-0.930680	2.329266
H	-5.481451	-3.372003	2.644339
H	-5.682573	-2.734085	5.621131
H	-4.366994	-3.603934	4.818256
H	-5.970269	-4.323953	4.907809
H	-7.825379	-3.572198	3.412566
H	-7.630221	-2.143753	2.392236
H	-7.738825	-1.967941	4.146811
H	-5.531779	-2.323265	-0.957542
H	-5.533618	-3.363079	-2.360899
H	-5.482608	-4.286528	0.538253
H	-6.011338	-6.543537	-0.402880
H	-4.407479	-5.990292	-0.870464
H	-5.741307	-5.913242	-2.030477
H	-7.758977	-4.296818	-1.497386
H	-7.621529	-3.202085	-0.117408
H	-7.841043	-4.934403	0.147835

H	-5.538414	0.678257	-4.056224
H	-5.532384	0.931226	-2.328627
H	-5.481900	3.372558	-2.643900
H	-5.684398	2.734368	-5.620541
H	-4.368313	3.604056	-4.818319
H	-5.971493	4.324362	-4.907255
H	-7.826138	3.572995	-3.411279
H	-7.630743	2.144755	-2.390710
H	-7.740098	1.968564	-4.145203
H	-5.531490	2.323150	0.958699
H	-5.532925	3.363421	2.361705
H	-5.483190	4.285878	-0.537783
H	-5.740589	5.913538	2.030473
H	-6.011487	6.543228	0.402784
H	-4.407373	5.990183	0.869726
H	-7.621790	3.201644	0.119443
H	-7.841419	4.933840	-0.146516
H	-7.758552	4.297032	1.498968
H	-1.483722	2.264594	-4.276417
H	-1.491925	4.277667	2.251866
H	-1.483433	-2.264777	4.276376
H	-1.491879	-4.276993	-2.251177
O	-1.454233	-4.936229	-0.500042
H	-0.579521	-5.203117	-0.170461
O	-1.443180	-0.510607	4.895605
H	-0.566325	-0.177866	5.152923
O	-1.453200	4.935335	0.500175
H	-0.577762	5.200560	0.171067
O	-1.444014	0.511077	-4.895318
H	-0.567537	0.177837	-5.153228
C	-1.490601	1.694771	4.014870
H	-0.688318	1.971886	4.683445
C	-1.485866	-4.037476	1.700768
H	-0.695289	-4.717743	1.984034
C	-1.485729	4.036999	-1.700768
H	-0.694923	4.716969	-1.984072
C	-1.491069	-1.694102	-4.014327
H	-0.688498	-1.970957	-4.682661

C	2.008784	2.583789	-3.160192
C	3.077527	2.269838	-2.325524
C	3.567497	0.972897	-2.400045
C	3.055613	-0.006863	-3.243220
C	1.980461	0.355890	-4.056095
C	3.644697	-1.401063	-3.305443
C	5.174690	-1.387822	-3.266454
C	5.832659	-2.751460	-3.457472
C	7.342159	-2.618645	-3.323907
C	3.654422	3.311665	-1.391450
C	5.184079	3.274866	-1.353931
C	5.835119	4.367579	-0.510931
C	7.346234	4.192814	-0.519021
O	1.455213	3.835134	-3.105613
C	3.046617	-2.324835	-2.265330
C	1.990497	-3.173704	-2.581984
C	1.972962	-4.073473	-0.354287
C	3.035773	-3.245715	0.009396
C	3.531185	-2.390336	-0.966869
O	1.444545	-3.129308	-3.836925
C	3.654358	-3.311846	1.391237
C	5.183999	-3.275457	1.353019
C	5.834307	-4.368550	0.509954
C	7.345472	-4.194189	0.517186
C	3.078010	-2.269816	2.325393
C	2.008971	-2.583370	3.159800
C	1.981238	-0.355462	4.055757
C	3.056734	0.006907	3.243181
C	3.568509	-0.973075	2.400151
O	1.454800	-3.834428	3.105110
C	3.646088	1.401009	3.305381
C	5.176040	1.387594	3.265948
C	5.834209	2.751163	3.456801
C	7.343635	2.618244	3.322517
C	3.047852	2.324856	2.265411
C	1.991738	3.173671	2.582184
C	1.973468	4.073131	0.354411
C	3.036336	3.245535	-0.009404

C	3.532116	2.390334	0.966816
O	1.446234	3.129490	3.837349
C	5.460181	-3.394870	-4.785186
C	5.446251	-5.765666	0.971254
C	5.462405	3.394472	4.784752
C	5.447207	5.764913	-0.971682
H	3.379413	-1.810540	-4.282871
H	3.393721	-4.283507	1.817023
H	3.381017	1.810428	4.282898
H	3.393880	4.283449	-1.817008
H	4.402214	0.708299	-1.762904
H	4.363468	1.747095	0.702308
H	4.403489	-0.708769	1.763239
H	4.362517	-1.747011	-0.702494
H	5.525382	-0.704524	-4.048553
H	5.528926	-0.964797	-2.321486
H	5.478888	-3.409576	-2.655490
H	5.721683	-2.739434	-5.622541
H	4.392811	-3.616373	-4.850631
H	5.996846	-4.336868	-4.921311
H	7.833545	-3.591493	-3.401474
H	7.619600	-2.179963	-2.361614
H	7.748387	-1.976583	-4.111978
H	5.534739	-2.304992	0.988691
H	5.545363	-3.353074	2.385088
H	5.485224	-4.248790	-0.522006
H	5.981132	-6.524516	0.395100
H	4.378108	-5.957153	0.848299
H	5.697422	-5.911787	2.027079
H	7.747920	-4.320525	1.527329
H	7.633214	-3.199166	0.166950
H	7.831767	-4.929101	-0.128822
H	5.526923	0.704217	4.047893
H	5.529950	0.964605	2.320844
H	5.480116	3.409387	2.655044
H	5.724373	2.738985	5.621922
H	4.395061	3.615923	4.850780
H	5.999107	4.336480	4.920658

H	7.835154	3.591023	3.400096
H	7.620601	2.179764	2.359994
H	7.750155	1.975953	4.110252
H	5.534742	2.304206	-0.990052
H	5.544945	3.352641	-2.386163
H	5.486564	4.247664	0.521187
H	5.697947	5.911260	-2.027577
H	5.982534	6.523467	-0.395554
H	4.379167	5.956649	-0.848201
H	7.633906	3.197643	-0.169146
H	7.833079	4.927466	0.126867
H	7.748157	4.319246	-1.529360
H	0.570415	3.549037	3.789971
H	0.581982	3.787602	-3.529976
H	0.568093	-3.547641	-3.788816
H	0.581448	-3.786247	3.529260
O	1.375096	-4.945588	0.506967
H	1.487818	-4.632124	1.424575
O	1.363537	-0.505712	-4.914177
H	1.485337	-1.424831	-4.610626
O	1.375273	4.945033	-0.506827
H	1.488691	4.632162	-1.424559
O	1.364278	0.506188	4.913745
H	1.486013	1.425355	4.610242
C	1.493185	1.650168	-4.036805
H	0.692021	1.928860	-4.706218
C	1.483219	-4.056011	-1.648105
H	0.690736	-4.735440	-1.927646
C	1.483995	4.055699	1.648302
H	0.691357	4.734903	1.927977
C	1.493563	-1.649574	4.036298
H	0.692153	-1.927986	4.705513

[**2**$_2$+Me$_4$N$_{endo}$]$^+$

Energy = -4835.34106386 hartree

Number of imaginary frequencies (PBE0-D3/def2-SVP) = 0

C	1.97976	-2.67146	3.06837
C	3.06396	-2.33576	2.25043
C	3.56132	-1.04080	2.36359
C	3.05604	-0.07934	3.23025
C	1.97752	-0.46344	4.02663
C	3.64121	1.31480	3.33537
C	5.17210	1.30145	3.31668
C	5.83162	2.66007	3.53711
C	7.34361	2.51345	3.45656
C	3.68151	-3.34510	1.30162
C	5.21093	-3.28932	1.30798
C	5.89504	-4.37069	0.47591
C	7.40188	-4.16433	0.50786
O	1.36877	-3.88394	3.04822
C	3.05391	2.26699	2.31051
C	1.97972	3.10347	2.62311
C	2.07415	4.09445	0.43327
C	3.13525	3.26926	0.06530
C	3.58936	2.38076	1.03199
O	1.32696	3.07553	3.81602
C	3.77241	3.36366	-1.31093
C	5.29999	3.30789	-1.25090
C	5.94749	4.39461	-0.39672
C	7.45367	4.18513	-0.35641
C	3.18755	2.34942	-2.27788
C	2.07376	2.66494	-3.06367
C	2.09159	0.46367	-4.03266
C	3.19522	0.09453	-3.26546
C	3.71397	1.06729	-2.41741
O	1.44246	3.86647	-3.03673
C	3.75430	-1.31268	-3.35461
C	5.28252	-1.34156	-3.28614
C	5.91124	-2.71755	-3.48871
C	7.42288	-2.61445	-3.35318
C	3.10921	-2.25048	-2.34570
C	2.03034	-3.07261	-2.68462
C	2.03121	-4.04672	-0.48357
C	3.09878	-3.24139	-0.09384

C	3.60138	-2.36816	-1.04922
O	1.42697	-3.06119	-3.90218
C	5.41629	3.29945	4.85395
C	5.60713	5.79548	-0.88405
C	5.52584	-3.34056	-4.82242
C	5.52957	-5.77454	0.93556
H	3.36406	1.69006	4.32317
H	3.52867	4.35305	-1.70561
H	3.49657	-1.68259	-4.34984
H	3.42194	-4.33621	1.68041
H	4.40970	-0.76982	1.74726
H	4.44888	-1.75084	-0.77376
H	4.58092	0.80879	-1.82217
H	4.43269	1.74818	0.77937
H	5.50718	0.60810	4.09632
H	5.54116	0.88672	2.37334
H	5.51391	3.32585	2.72619
H	5.63546	2.63618	5.69684
H	4.35097	3.54021	4.88043
H	5.96127	4.23156	5.01731
H	7.83894	3.48221	3.55000
H	7.65230	2.07115	2.50548
H	7.71700	1.87071	4.25956
H	5.63378	2.33406	-0.87900
H	5.67917	3.38102	-2.27634
H	5.56544	4.29261	0.62606
H	6.13744	6.54680	-0.29507
H	4.54026	6.01610	-0.80332
H	5.90190	5.92681	-1.93010
H	7.88919	4.28981	-1.35479
H	7.70896	3.18915	0.01548
H	7.93640	4.91766	0.29396
H	5.66200	-0.65645	-4.05262
H	5.63243	-0.93943	-2.33006
H	5.54574	-3.37703	-2.69280
H	5.79488	-2.68134	-5.65395
H	4.45567	-3.54987	-4.88883
H	6.04915	-4.28769	-4.96967

H	7.89389	-3.59630	-3.43544
H	7.70958	-2.18646	-2.38877
H	7.84274	-1.97810	-4.13813
H	5.56077	-2.31288	0.95843
H	5.54484	-3.36972	2.34854
H	5.56141	-4.25962	-0.56259
H	5.77398	-5.91465	1.99340
H	6.08601	-6.52233	0.36658
H	4.46741	-5.99240	0.80213
H	7.67603	-3.16498	0.15912
H	7.91283	-4.89088	-0.12745
H	7.79035	-4.28079	1.52418
H	1.58292	-2.20610	-4.34572
H	1.53292	-4.33334	2.19869
H	1.47729	2.22456	4.26725
H	1.62898	4.33943	-2.20532
O	1.57342	4.98245	-0.47778
H	0.72412	5.31057	-0.14637
O	1.41173	0.45457	4.86534
H	0.56287	0.10302	5.17928
O	1.47205	-4.89853	0.42715
H	0.61704	-5.20247	0.08149
O	1.52739	-0.45164	-4.87683
H	0.68224	-0.09640	-5.19371
C	1.46462	-1.74492	3.96111
H	0.64286	-2.02953	4.60350
C	1.52156	4.02722	1.69905
H	0.72022	4.69941	1.97309
C	1.52420	-3.98041	-1.76636
H	0.72445	-4.64527	-2.06213
C	1.55552	1.73301	-3.94716
H	0.71723	2.00478	-4.57344
C	-2.00683	-2.49274	-3.16679
C	-3.10354	-2.20238	-2.35692
C	-3.63077	-0.92090	-2.46231
C	-3.12400	0.07185	-3.29746
C	-2.01772	-0.26706	-4.08252
C	-3.73092	1.46259	-3.35001

C	-5.25980	1.43849	-3.30581
C	-5.92613	2.80038	-3.48273
C	-7.43394	2.65556	-3.34291
C	-3.67944	-3.26500	-1.44236
C	-5.20971	-3.23370	-1.40503
C	-5.85850	-4.35541	-0.59874
C	-7.36995	-4.18338	-0.60765
O	-1.43848	-3.73266	-3.10017
C	-3.12857	2.36972	-2.29364
C	-2.04307	3.19328	-2.58353
C	-1.98335	4.01137	-0.32103
C	-3.08284	3.21626	0.01542
C	-3.61292	2.41757	-0.99219
O	-1.51216	3.17799	-3.84337
C	-3.70466	3.27848	1.39848
C	-5.23490	3.24072	1.36152
C	-5.88952	4.34264	0.53271
C	-7.40025	4.16466	0.54819
C	-3.13493	2.23886	2.34373
C	-2.09695	2.55730	3.21903
C	-2.07583	0.31977	4.10194
C	-3.12292	-0.04682	3.25187
C	-3.61665	0.93723	2.39901
O	-1.54150	3.80483	3.16687
C	-3.71533	-1.44203	3.28788
C	-5.24560	-1.42112	3.22819
C	-5.91130	-2.78410	3.39710
C	-7.41855	-2.63899	3.25129
C	-3.10996	-2.35842	2.24431
C	-2.08480	-3.24389	2.57161
C	-2.03163	-4.09441	0.32066
C	-3.07076	-3.23671	-0.05162
C	-3.56817	-2.39046	0.93324
O	-1.57018	-3.21829	3.83688
C	-5.56402	3.45515	-4.80779
C	-5.50060	5.73447	1.00909
C	-5.55487	-3.44387	4.72126
C	-5.46530	-5.73583	-1.10409

H	-3.46990	1.88705	-4.32219
H	-3.44740	4.25178	1.82291
H	-3.46888	-1.86768	4.26305
H	-3.41784	-4.22647	-1.89041
H	-4.49497	-0.67793	-1.85651
H	-4.38539	-1.73021	0.66636
H	-4.43420	0.67179	1.74017
H	-4.47481	1.80581	-0.75170
H	-5.60781	0.76111	-4.09364
H	-5.60999	1.00364	-2.36438
H	-5.57406	3.45551	-2.67696
H	-5.82759	2.80557	-5.64858
H	-4.49869	3.68593	-4.87986
H	-6.10685	4.39434	-4.93368
H	-7.93121	3.62545	-3.41117
H	-7.70528	2.20819	-2.38286
H	-7.83987	2.01812	-4.13429
H	-5.58653	2.27239	0.99145
H	-5.59234	3.30930	2.39512
H	-5.54849	4.23611	-0.50360
H	-6.04147	6.49837	0.44652
H	-4.43420	5.93390	0.88027
H	-5.74532	5.86799	2.06773
H	-7.79690	4.28094	1.56135
H	-7.68971	3.17323	0.18926
H	-7.89083	4.90535	-0.08686
H	-5.60230	-0.74500	4.01325
H	-5.58554	-0.98584	2.28333
H	-5.55572	-3.43564	2.58999
H	-5.82141	-2.79717	5.56336
H	-4.49012	-3.67635	4.79712
H	-6.09921	-4.38289	4.84156
H	-7.91547	-3.60961	3.31060
H	-7.68567	-2.18527	2.29310
H	-7.82843	-2.00714	4.04509
H	-5.56457	-2.27605	-1.01120
H	-5.56805	-3.28043	-2.43956
H	-5.51561	-4.26809	0.43897

H	-5.71070	-5.84811	-2.16502
H	-6.00324	-6.51298	-0.55707
H	-4.39823	-5.93481	-0.98049
H	-7.66270	-3.20061	-0.22830
H	-7.85632	-4.93880	0.01316
H	-7.76806	-4.28093	-1.62221
H	-0.73396	-3.70753	3.83615
H	-0.59408	-3.70867	-3.57855
H	-0.66534	3.64872	-3.82411
H	-0.68276	3.77578	3.61900
O	-1.35915	4.83853	0.55561
H	-1.54450	4.56909	1.47436
O	-1.39231	0.59583	-4.92495
H	-1.56811	1.51797	-4.66288
O	-1.40708	-4.93467	-0.54733
H	-1.54022	-4.62762	-1.46326
O	-1.45361	-0.54737	4.94524
H	-1.62946	-1.46626	4.67766
C	-1.49096	-1.54773	-4.03220
H	-0.66402	-1.80733	-4.67782
C	-1.48788	4.00857	-1.61522
H	-0.65990	4.65579	-1.86838
C	-1.57455	-4.11822	1.62952
H	-0.79238	-4.81274	1.90569
C	-1.60341	1.62259	4.11002
H	-0.81920	1.89973	4.80174
C	-1.00591	0.33561	-0.85391
H	-1.97493	0.13739	-0.40483
H	-0.91153	1.39450	-1.08202
H	-0.88458	-0.26248	-1.75378
C	-0.08067	-1.47484	0.43544
H	0.04837	-2.05034	-0.47847
H	0.69212	-1.73656	1.15352
H	-1.06720	-1.64313	0.85649
C	-0.12104	0.75936	1.34387
H	0.65111	0.46824	2.05137
H	-0.01947	1.81299	1.09343
H	-1.10769	0.55712	1.74942

C	1.37250	0.22392	-0.47877
H	1.45001	1.28211	-0.70744
H	2.13867	-0.06008	0.23892
H	1.47436	-0.36592	-1.38424
N	0.04703	-0.03884	0.11209

[**2**$_2$+I$_{exo}$]$^-$

Energy = -4919.14337327hartree

Number of imaginary frequencies (PBE0-D3/def2-SVP) = 0

C	-2.43684	-2.64591	-3.10016
C	-3.51602	-2.31976	-2.27615
C	-4.02602	-1.03088	-2.38128
C	-3.52376	-0.06365	-3.24186
C	-2.43693	-0.43084	-4.03193
C	-4.10175	1.33275	-3.33194
C	-5.63136	1.33207	-3.28859
C	-6.28137	2.69921	-3.48082
C	-7.79338	2.57283	-3.37147
C	-4.09916	-3.34301	-1.32473
C	-5.62885	-3.31220	-1.30114
C	-6.27957	-4.40122	-0.45310
C	-7.78967	-4.21767	-0.44457
O	-1.84129	-3.87045	-3.10180
C	-3.49106	2.27462	-2.31468
C	-2.42832	3.11448	-2.65181
C	-2.42630	4.05301	-0.43846
C	-3.49689	3.24628	-0.06176
C	-3.98698	2.37100	-1.02075
O	-1.84925	3.12315	-3.88428
C	-4.09929	3.34296	1.32467
C	-5.62898	3.31215	1.30086
C	-6.27956	4.40121	0.45278
C	-7.78966	4.21767	0.44402
C	-3.51627	2.31968	2.27613
C	-2.43712	2.64577	3.10020

C	-2.43743	0.43071	4.03202
C	-3.52423	0.06359	3.24189
C	-4.02637	1.03085	2.38127
O	-1.84143	3.87025	3.10181
C	-4.10229	-1.33279	3.33190
C	-5.63190	-1.33207	3.28834
C	-6.28196	-2.69919	3.48049
C	-7.79396	-2.57278	3.37089
C	-3.49146	-2.27464	2.31471
C	-2.42860	-3.11432	2.65193
C	-2.42623	-4.05280	0.43855
C	-3.49695	-3.24628	0.06178
C	-3.98726	-2.37110	1.02073
O	-1.84965	-3.12298	3.88445
C	-5.88413	3.35111	-4.79703
C	-5.90610	5.80066	0.91917
C	-5.88494	-3.35107	4.79676
C	-5.90604	-5.80068	-0.91938
H	-3.83304	1.70993	-4.32135
H	-3.82812	4.32483	1.71934
H	-3.83372	-1.70998	4.32134
H	-3.82793	-4.32489	-1.71934
H	-4.86560	-0.76351	-1.75128
H	-4.82014	-1.73312	0.74716
H	-4.86594	0.76352	1.75122
H	-4.81977	1.73288	-0.74723
H	-5.98840	0.64940	-4.06889
H	-5.98718	0.91286	-2.34240
H	-5.93657	3.34988	-2.66930
H	-6.12881	2.70065	-5.64364
H	-4.81544	3.57117	-4.83824
H	-6.41785	4.29463	-4.93734
H	-8.27929	3.54911	-3.44585
H	-8.08689	2.12495	-2.41807
H	-8.19058	1.94039	-4.17224
H	-5.98526	2.34083	0.94427
H	-5.98391	3.39827	2.33467
H	-5.91834	4.28427	-0.57529

H	-6.43459	6.55722	0.33347
H	-4.83683	5.99563	0.81519
H	-6.17517	5.94516	1.97100
H	-8.20223	4.33386	1.45164
H	-8.06684	3.22310	0.08404
H	-8.27503	4.95409	-0.20163
H	-5.98902	-0.64938	4.06858
H	-5.98757	-0.91285	2.34209
H	-5.93705	-3.34988	2.66903
H	-6.12971	-2.70058	5.64332
H	-4.81627	-3.57118	4.83813
H	-6.41871	-4.29456	4.93703
H	-8.27990	-3.54905	3.44523
H	-8.08730	-2.12493	2.41744
H	-8.19127	-1.94032	4.17159
H	-5.98519	-2.34086	-0.94466
H	-5.98364	-3.39836	-2.33500
H	-5.91849	-4.28423	0.57501
H	-6.17494	-5.94523	-1.97125
H	-6.43463	-6.55722	-0.33373
H	-4.83678	-5.99566	-0.81522
H	-8.06689	-3.22308	-0.08470
H	-8.27512	-4.95405	0.20107
H	-8.20209	-4.33394	-1.45223
H	-1.91684	-2.23308	4.28330
H	-1.91821	-4.27866	-2.21793
H	-1.91633	2.23323	-4.28310
H	-1.91827	4.27841	2.21792
O	-1.85586	4.89399	0.47744
H	-0.95280	5.09825	0.16501
O	-1.85335	0.49166	-4.85614
H	-0.95110	0.17499	-5.05820
O	-1.85546	-4.89360	-0.47732
H	-0.95233	-5.09744	-0.16478
O	-1.85392	-0.49183	4.85622
H	-0.95159	-0.17527	5.05816
C	-1.93084	-1.71488	-3.98851
H	-1.11146	-1.98786	-4.63713

C	-1.92634	4.01106	-1.72561
H	-1.12018	4.67296	-2.00681
C	-1.92637	-4.01077	1.72573
H	-1.12001	-4.67242	2.00696
C	-1.93126	1.71473	3.98861
H	-1.11192	1.98766	4.63730
C	1.58035	-2.63085	3.14706
C	2.63661	-2.30326	2.30427
C	3.12804	-1.00437	2.38487
C	2.61006	-0.03721	3.24285
C	1.53503	-0.41129	4.04896
C	3.19972	1.35670	3.33186
C	4.72944	1.33265	3.32464
C	5.39368	2.69499	3.49433
C	6.90489	2.53141	3.43155
C	3.20541	-3.33766	1.35652
C	4.73523	-3.32297	1.33993
C	5.38170	-4.38215	0.45346
C	6.89475	-4.23645	0.51417
O	1.03755	-3.89397	3.09788
C	2.61809	2.29180	2.29240
C	1.56917	3.14274	2.62196
C	1.52988	4.04910	0.40454
C	2.59833	3.23539	0.02918
C	3.10786	2.36880	0.99286
O	1.03300	3.10098	3.88805
C	3.20529	3.33764	-1.35641
C	4.73510	3.32300	-1.33982
C	5.38152	4.38221	-0.45335
C	6.89458	4.23655	-0.51402
C	2.63650	2.30325	-2.30416
C	1.58024	2.63084	-3.14698
C	1.53499	0.41127	-4.04888
C	2.61000	0.03719	-3.24275
C	3.12795	1.00437	-2.38476
O	1.03743	3.89392	-3.09776
C	3.19973	-1.35670	-3.33175
C	4.72945	-1.33254	-3.32455

C	5.39378	-2.69483	-3.49428
C	6.90498	-2.53117	-3.43150
C	2.61818	-2.29186	-2.29230
C	1.56941	-3.14296	-2.62191
C	1.53011	-4.04932	-0.40449
C	2.59844	-3.23546	-0.02908
C	3.10790	-2.36881	-0.99273
O	1.03333	-3.10131	-3.88805
C	4.96850	3.40626	4.77086
C	4.94717	5.79667	-0.80912
C	4.96866	-3.40609	-4.77084
C	4.94736	-5.79661	0.80921
H	2.91254	1.75739	4.30743
H	2.92054	4.31300	-1.75930
H	2.91258	-1.75742	-4.30732
H	2.92072	-4.31302	1.75945
H	3.96216	-0.72987	1.74466
H	3.94623	-1.73113	-0.71964
H	3.96207	0.72988	-1.74454
H	3.94627	1.73123	0.71981
H	5.05452	0.67060	4.13677
H	5.10804	0.88376	2.40212
H	5.09111	3.32008	2.64617
H	5.18361	2.78701	5.64905
H	3.90111	3.64105	4.77813
H	5.51180	4.34817	4.88861
H	7.40901	3.50165	3.46795
H	7.20164	2.01906	2.51300
H	7.26401	1.93562	4.27797
H	5.11252	2.34423	-1.03082
H	5.07635	3.46334	-2.37306
H	5.07011	4.18725	0.57927
H	5.48008	6.53081	-0.19820
H	3.87737	5.95266	-0.64826
H	5.16633	6.01715	-1.85997
H	7.26316	4.45050	-1.52335
H	7.19592	3.21788	-0.25729
H	7.38607	4.92942	0.17531

H	5.05448	-0.67045	-4.13667
H	5.10803	-0.88364	-2.40202
H	5.09124	-3.31997	-2.64615
H	5.18374	-2.78680	-5.64901
H	3.90129	-3.64094	-4.77813
H	5.51201	-4.34796	-4.88861
H	7.40917	-3.50136	-3.46794
H	7.20170	-2.01883	-2.51293
H	7.26407	-1.93531	-4.27789
H	5.11260	-2.34417	1.03093
H	5.07648	-3.46329	2.37317
H	5.07030	-4.18718	-0.57916
H	5.16649	-6.01710	1.86007
H	5.48029	-6.53074	0.19829
H	3.87756	-5.95261	0.64832
H	7.19607	-3.21778	0.25745
H	7.38628	-4.92931	-0.17516
H	7.26331	-4.45040	1.52351
H	0.17469	-3.54898	-3.84827
H	0.17907	-3.85267	3.54539
H	0.17435	3.54865	3.84820
H	0.17887	3.85266	-3.54514
O	0.90485	4.91651	-0.45321
H	1.04769	4.62564	-1.37383
O	0.89727	0.44836	4.90476
H	1.04715	1.36867	4.61785
O	0.90511	-4.91678	0.45322
H	1.04800	-4.62597	1.37384
O	0.89719	-0.44837	-4.90466
H	1.04706	-1.36868	-4.61774
C	1.05611	-1.70793	4.02967
H	0.26082	-1.99685	4.70273
C	1.04892	4.02907	1.70020
H	0.25910	4.70777	1.99051
C	1.04924	-4.02937	-1.70018
H	0.25952	-4.70817	-1.99054
C	1.05608	1.70792	-4.02963
H	0.26087	1.99685	-4.70279

I	6.45500	0.00006	0.00004

[**2**$_2$+2I$_{exo}$]$^{2-}$

Energy = -5216.96082423 hartree

Number of imaginary frequencies (PBE0-D3/def2-SVP) = 0

C	1.99649	2.61468	-3.12519
C	3.07558	2.29805	-2.29870
C	3.59132	1.00782	-2.38991
C	3.08829	0.03720	-3.25023
C	2.01386	0.40386	-4.05452
C	3.66138	-1.36256	-3.32970
C	5.19114	-1.35742	-3.31139
C	5.84380	-2.72802	-3.45815
C	7.35672	-2.57644	-3.40750
C	3.66197	3.33494	-1.36171
C	5.19162	3.31372	-1.36559
C	5.85146	4.38554	-0.50414
C	7.36316	4.22642	-0.56627
O	1.37772	3.83485	-3.12311
C	3.06028	-2.28733	-2.28986
C	1.98972	-3.12249	-2.61162
C	2.00883	-4.05668	-0.40340
C	3.07398	-3.24295	-0.03156
C	3.57029	-2.37349	-0.99731
O	1.38131	-3.12648	-3.83676
C	3.66121	-3.33505	1.36177
C	5.19086	-3.31412	1.36575
C	5.85053	-4.38610	0.50438
C	7.36225	-4.22702	0.56617
C	3.07497	-2.29806	2.29873
C	1.99577	-2.61451	3.12515
C	2.01364	-0.40376	4.05462
C	3.08809	-0.03724	3.25031
C	3.59094	-1.00793	2.38995
O	1.37679	-3.83455	3.12301
C	3.66140	1.36241	3.32977

C	5.19117	1.35702	3.31160
C	5.84403	2.72751	3.45854
C	7.35692	2.57579	3.40764
C	3.06051	2.28724	2.28985
C	1.98992	3.12241	2.61145
C	2.00951	4.05677	0.40331
C	3.07462	3.24293	0.03157
C	3.57072	2.37340	0.99739
O	1.38115	3.12626	3.83642
C	5.40340	-3.46121	-4.71691
C	5.42781	-5.79676	0.88870
C	5.40388	3.46047	4.71752
C	5.42869	5.79629	-0.88809
H	3.37294	-1.75467	-4.30839
H	3.37203	-4.31188	1.75795
H	3.37296	1.75460	4.30843
H	3.37302	4.31183	-1.75790
H	4.43088	0.74355	-1.75334
H	4.41258	1.73749	0.73278
H	4.43054	-0.74381	1.75338
H	4.41216	-1.73763	-0.73260
H	5.52855	-0.71017	-4.13081
H	5.57052	-0.89861	-2.39405
H	5.54066	-3.33353	-2.59648
H	5.61313	-2.85992	-5.60908
H	4.33460	-3.68849	-4.70789
H	5.94009	-4.40894	-4.82126
H	7.85272	-3.55188	-3.42479
H	7.66256	-2.04597	-2.50206
H	7.71592	-2.00241	-4.26924
H	5.56946	-2.34059	1.04214
H	5.52211	-3.43402	2.40494
H	5.53948	-4.21548	-0.53265
H	5.96649	-6.53899	0.29202
H	4.35912	-5.96352	0.73330
H	5.64970	-5.99328	1.94396
H	7.72887	-4.41682	1.58138
H	7.65645	-3.21121	0.28993

H	7.86176	-4.93062	-0.10710
H	5.52838	0.70966	4.13102
H	5.57056	0.89818	2.39428
H	5.54080	3.33324	2.59705
H	5.61366	2.85895	5.60952
H	4.33511	3.68789	4.70870
H	5.94069	4.40812	4.82202
H	7.85302	3.55119	3.42512
H	7.66261	2.04550	2.50205
H	7.71617	2.00151	4.26920
H	5.57003	2.34012	-1.04198
H	5.52295	3.43362	-2.40475
H	5.54063	4.21472	0.53292
H	5.65032	5.99302	-1.94336
H	5.96754	6.53837	-0.29138
H	4.36004	5.96304	-0.73240
H	7.65736	3.21054	-0.29028
H	7.86283	4.92985	0.10706
H	7.72959	4.41642	-1.58151
H	1.48128	2.24755	4.25201
H	1.48566	4.25439	-2.24798
H	1.48131	-2.24773	-4.25230
H	1.48449	-4.25401	2.24780
O	1.44513	-4.90937	0.51560
H	0.55566	-5.13081	0.18978
O	1.43791	-0.51980	-4.89351
H	0.54589	-0.19336	-5.10531
O	1.44607	4.90959	-0.51573
H	0.55660	5.13115	-0.19003
O	1.43802	0.51994	4.89382
H	0.54605	0.19356	5.10587
C	1.50030	1.68478	-4.01917
H	0.68844	1.95525	-4.67924
C	1.49588	-4.02077	-1.68450
H	0.69110	-4.68794	-1.95901
C	1.49634	4.02082	1.68432
H	0.69157	4.68804	1.95874
C	1.49975	-1.68454	4.01915

H	0.68780	-1.95481	4.67919
C	-2.01752	2.58731	3.15346
C	-3.09131	2.27698	2.32499
C	-3.59180	0.98116	2.40108
C	-3.07394	0.00399	3.24724
C	-1.99519	0.36543	4.05549
C	-3.65945	-1.39226	3.31799
C	-5.18911	-1.37374	3.30794
C	-5.85040	-2.73976	3.45959
C	-7.36221	-2.58011	3.40063
C	-3.66450	3.32250	1.39113
C	-5.19419	3.30543	1.37918
C	-5.84464	4.37720	0.51070
C	-7.35756	4.23014	0.57228
O	-1.44463	3.83548	3.09587
C	-3.07323	-2.31347	2.26790
C	-2.00717	-3.15089	2.58031
C	-1.99054	-4.05607	0.36001
C	-3.06197	-3.23930	-0.00288
C	-3.57121	-2.38456	0.97106
O	-1.44153	-3.10164	3.83198
C	-3.66475	-3.32216	-1.39117
C	-5.19446	-3.30493	-1.37943
C	-5.84516	-4.37662	-0.51106
C	-7.35807	-4.22983	-0.57344
C	-3.09134	-2.27676	-2.32503
C	-2.01763	-2.58732	-3.15351
C	-1.99477	-0.36540	-4.05543
C	-3.07349	-0.00377	-3.24725
C	-3.59156	-0.98085	-2.40111
O	-1.44515	-3.83568	-3.09599
C	-3.65885	1.39255	-3.31801
C	-5.18851	1.37411	-3.30808
C	-5.84977	2.74018	-3.45948
C	-7.36158	2.58049	-3.40087
C	-3.07266	2.31367	-2.26784
C	-2.00638	3.15088	-2.58010
C	-1.98996	4.05616	-0.35984

C	-3.06158	3.23957	0.00291
C	-3.57082	2.38489	-0.97109
O	-1.44050	3.10149	-3.83166
C	-5.42143	-3.46769	4.72543
C	-5.41007	-5.78600	-0.88580
C	-5.42054	3.46848	-4.72502
C	-5.40998	5.78656	0.88602
H	-3.36944	-1.80237	4.28880
H	-3.37737	-4.29197	-1.80545
H	-3.36873	1.80268	-4.28879
H	-3.37700	4.29225	1.80546
H	-4.43102	0.71685	1.76408
H	-4.41350	1.74937	-0.70816
H	-4.43076	-0.71640	-1.76414
H	-4.41374	-1.74890	0.70800
H	-5.51843	-0.72243	4.12733
H	-5.56889	-0.91487	2.39084
H	-5.54612	-3.35209	2.60301
H	-5.63596	-2.86063	5.61251
H	-4.35335	-3.69837	4.72639
H	-5.96205	-4.41304	4.83104
H	-7.86376	-3.55263	3.42230
H	-7.66105	-2.05383	2.49045
H	-7.72211	-1.99824	4.25678
H	-5.57266	-2.33124	-1.05570
H	-5.53408	-3.42796	-2.41565
H	-5.53560	-4.19692	0.52475
H	-5.94492	-6.52901	-0.28666
H	-4.34066	-5.94270	-0.72477
H	-5.62619	-5.99079	-1.94071
H	-7.72377	-4.42982	-1.58705
H	-7.65977	-3.21436	-0.30403
H	-7.85183	-4.93226	0.10526
H	-5.51779	0.72298	-4.12763
H	-5.56837	0.91507	-2.39110
H	-5.54568	3.35226	-2.60265
H	-5.63485	2.86168	-5.61232
H	-4.35247	3.69921	-4.72567

H	-5.96118	4.41384	-4.83047
H	-7.86314	3.55302	-3.42233
H	-7.66059	2.05393	-2.49091
H	-7.72131	1.99889	-4.25729
H	-5.57239	2.33178	1.05537
H	-5.53399	3.42847	2.41533
H	-5.53449	4.19780	-0.52499
H	-5.62685	5.99111	1.94081
H	-5.94450	6.52963	0.28666
H	-4.34048	5.94343	0.72575
H	-7.65897	3.21470	0.30244
H	-7.85110	4.93271	-0.10643
H	-7.72378	4.42974	1.58577
H	-0.55016	3.48666	-3.75589
H	-0.55437	3.75741	3.48016
H	-0.55144	-3.48747	3.75660
H	-0.55504	-3.75806	-3.48064
O	-1.38253	-4.92658	-0.50261
H	-1.48437	-4.60138	-1.41831
O	-1.37399	-0.49898	4.91471
H	-1.48111	-1.41448	4.59192
O	-1.38201	4.92666	0.50281
H	-1.48381	4.60138	1.41848
O	-1.37331	0.49891	-4.91456
H	-1.48018	1.41442	-4.59166
C	-1.50151	1.65619	4.03215
H	-0.68974	1.93229	4.69009
C	-1.49497	-4.03278	1.64997
H	-0.68885	-4.69704	1.92734
C	-1.49416	4.03269	-1.64970
H	-0.68780	4.69670	-1.92696
C	-1.50136	-1.65629	-4.03214
H	-0.68964	-1.93251	-4.69009
I	7.00615	-0.00028	-0.00002
I	-7.00520	0.00010	-0.00023
